# New Wavenumber Calibration Tables From Heterodyne Frequency Measurements

**DOI:** 10.6028/jres.097.019

**Published:** 1992

**Authors:** Arthur G. Maki, Joseph S. Wells

**Affiliations:** National Institute of Standards and Technology, Gaithersburg, MD 20899; National Institute of Standards and Technology, Boulder, CO 80303

**Keywords:** calibration atlas, carbon disulfide, carbon monoxide, carbonyl sulfide, IR frequency calibrations, IR wavenumber calibrations, nitric oxide, nitrous oxide, wavenumber tables

## Abstract

This new calibration atlas is based on frequency rather than wavelength calibration techniques for absolute references. Since a limited number of absolute frequency measurements is possible, additional data from alternate methodology are used for difference frequency measurements within each band investigated by the frequency measurements techniques. Data from these complementary techniques include the best Fourier transform measurements available. Included in the text relating to the atlas are a description of the heterodyne frequency measurement techniques and details of the analysis, including the Hamiltonians and least-squares-fitting and calculation. Also included are other relevant considerations such as intensities and lincshape parameters. A 390-entry bibliography which contains all data sources used and a subsequent section on errors conclude the text portion.

The primary calibration molecules are the linear triatomics, carbonyl sulfide and nitrous oxide, which cover portions of the infrared spectrum ranging from 488 to 3120 cm^−1^. Some gaps in the coverage afforded by OCS and N_2_O are partially covered by NO, CO, and CS_2_. An additional region from 4000 to 4400 cm^−1^ is also included.

The tabular portion of the atlas is too lengthy to include in an archival journal. Furthermore, different users have different requirements for such an atlas. In an effort to satisfy most users, we have made two different options available. The first is NIST Special Publication 821, which has a spectral map/facing table format. The spectral maps (as well as the facing tables) are calculated from molecular constants derived for the work. A complete list of all of the molecular transitions that went into making the maps is too long (perhaps by a factor of 4 or 5) to include in the facing tables. The second option for those not interested in maps (or perhaps to supplement Special Publication 821) is the complete list (tables-only) which is available in computerized format as NIST Standard Reference Database *#*39, Wavelength Calibration Tables.

## 1. Introduction

The primary purpose of this work is to provide an atlas of molecular spectra and associated tables of wavenumbers to be used for the calibration of infrared spectrometers. A secondary purpose is to furnish a detailed description of the infrared heterodyne frequency measurement techniques developed for this work. Additionally, we provide a bibliography of all the measurements used in producing these tables, as well as to provide a description of how those measurements were combined to calculate energy levels, transitions, and uncertainties. We also provide useful related information such as line intensities, pressure broadening coefficients, and estimates of pressure shifts of spectral lines. This book does not include an exhaustive list of all the weaker transition frequencies currently available, especially for the less abundant molecular species. Such a list, containing over 10 000 transitions for OCS, is available, however, in computerized format as NIST Standard Reference Database 39. To put this work in proper perspective, some background, philosophy, and the status of existing atlases are discussed in the following sections.

Over the last 35 years (since the work of Downie et al.^3^ [1.1]) several compilations of infrared absorption spectra intended for the calibration of infrared spectrometers have appeared. Two compilations have been published by the International Union of Pure and Applied Chemistry (IUPAC) [1.2,1.3] and contain sections pertaining to the calibration of fairly low resolution (0.5 cm^−1^) instruments. Other compilations [1.4–1.7] and other sections of the IUPAC compilations [1.2,1.3] were devoted to data intended for the calibration of high resolution instruments (resolution better than 0.5 cm^−1^). This book falls into the latter category. Of the earlier compilations, only the work of Guelachvili and Rao [1.7] provides calibration data that are consistently more accurate than ±0.01 cm^−1^.

A number of commercially available infrared spectrometers are capable of recording spectra with a resolution of 0.06 cm^−1^ or better. For the calibration of such spectrometers, one needs calibration data with an uncertainty smaller than 1/30th of the spectral linewidth. For a resolution of 0.06 cm^−1^ this means an accuracy of 0.002 cm^−1^. For Doppler-limited resolution at room temperatures, a calibration accuracy of 0.0002 cm^−1^ or better would be desirable. The present work responds to these needs, even though it was recognized that state-of-the-art instrumentation (for sub-Doppler measurements, for example) could use even more accurate calibration data.

Most high resolution spectrometers are not capable of broad frequency scans. For the most accurate measurements the calibration should be applied to each spectral scan. This requires that at least one and preferably several calibration points be available within the scanning range of the spectrometer. For tunable diode laser spectrometers, a single scan may cover only 0.5 cm^−1^, while for a Fourier transform spectrometer (FTS) a high resolution scan may cover hundreds of wavenumbers. This means that for many purposes calibration standards should be no more than 100 cm^−1^ apart, while for other purposes standards no more than 0.5 cm^−1^ apart are required throughout the infrared region of interest. Our work here presents a compromise between these two requirements.

In our opinion, all previous compilations of infrared wavenumber standards have suffered from the lack of a consistent effort to draw together a number of experimental measurements to arrive at a well determined set of molecular energy levels which could be used to determine frequency (or wavenumber) standards for a number of bands throughout the infrared spectral region. The present compilation provides a model for producing such frequency or wavenumber calibration data.

It is appropriate at this point to define some of the terminology used in this book clarifying an important distinction: in some places emphasis is placed on the difference between frequency measurements and wavelength measurements. All measurements that can be reduced to counting frequencies or frequency differences are frequency measurements, while all measurements that are really comparisons of wavelengths (or wavenumbers), including counting fringes, are wavelength measurements. Fourier transform measurements, for instance are truly wavelength measurements even though counting (of fringes of a laser beam passed through the spectrometer, for example) may be involved.

Most measurements of infrared absorption spectra are made using grating or Fourier transform spectrometers. These measurements are truly wavelength measurements. They rely for calibration on measuring the difference in wavelength of some calibration feature and the feature of the spectrum to be determined. For FTS instruments, the position of the moveable mirror must be determined, while for grating instruments one is essentially calibrating the grating angle and the spacing of the grooves of the grating. Very often these instruments use more than one beam and compare the wavelength of a calibration beam with the beam carrying the spectra of interest. With FTS instruments a helium-neon laser beam is often used to monitor the mirror position. In other cases a single beam is used, but one cannot be certain that the beam properties are independent of wavelength. With all wavelength measuring instruments, it is extremely important that the calibration light beam follow precisely the same path through the spectrometer as the beam of light being measured. Problems of wavelength-dependent diffraction effects become very important for measurements intended to approach or exceed one part in 10^7^. One of the most insidious problems with wavelength measurements is the risk (or ease) of incurring systematic errors and the virtual impossibility of detecting them.

The great advantage of frequency measurements is that they simply depend on frequency counting techniques. Over the years considerable attention has been given to electronic techniques of frequency counting, and simple, accurate, well calibrated devices are readily available. The accuracy of frequency techniques does not depend on whether or not the beams are perfectly collinear or have parallel wavefronts. If a countable signal (25 dB signal-to-noise ratio) is obtained, it will be correct. The frequency of light does not depend on the medium, nor does it depend on the angle from which it is viewed.

Over the past decade we have striven to provide infrared heterodyne frequency measurements on the transitions and energy levels of several simple molecules, which are good candidates for frequency calibration standards. OCS, CO, and N_2_O were chosen because they are stable, safe to handle, easily obtainable, and well documented in terms of good measurements reported in existing literature. Furthermore, they are particularly amenable to the accurate calculation of energy levels from a relatively simple Hamiltonian.

For these molecules a least-squares fit of many transitions (over 3000 OCS lines for example) has permitted the determination of all of the lower energy separations, using frequency differences referred to the primary cesium frequency standard. Due to statistical improvements from a large data base, transition frequencies between these energy levels can be calculated with greater accuracy than any single measurement with its attendant random errors.

Although we have had to use some FTS (wavelength) measurements to help define certain higher order rotational constants, for the most part the energy levels were determined from *frequency* measurements because they are less susceptible to unknown systematic errors than are wavelength measurements. Particular importance was attached to estimating the uncertainties in the transition wavenumbers; see the discussion in the chapter on errors (Sec. 4).

As with any good calibration standard, a number of different measurements were used in determining the energy levels and transition frequencies. However, only a few laboratories have used frequency measurement techniques in the infrared region and very few of the more accurate sub-Doppler frequency measurements have been made, so it is somewhat premature to claim the level of accuracy that we desire for infrared standards. Nevertheless we are encouraged by the convergence of different FTS measurements on the same values for the band centers as frequency measurements. Our publication of this atlas at this time is dictated by the need to provide good calibration data now, rather than await the arrival of a perfect atlas.

Of course the combination of OCS, CO, and N_2_O gases is insufficient to provide calibration data everywhere within the infrared, so we have had to provide heterodyne measurements on other molecular species, such as CS_2_ and NO in order to fill some of the gaps. The user will still note that many gaps remain in the coverage of these tables. To some extent these gaps may be filled by using the data provided by the compilation of Guelachvili and Rao [1.7]. We also expect that future measurements will provide calibration data where none are currently available.

Since most workers are more comfortable with calibration data given in wavenumber units, the tables in this book are given in wavenumbers (cm^−1^) even though the values were primarily determined from frequency measurements. The conversion from frequency units to wavenumber units was made by using the defined value of the velocity of light, *c* = 299 792 458 m/s. Since the tables are given in wavenumbers, we often use the terms wavenumber and frequency interchangeably in the text, but the term wavelength is reserved for quantities determined by wavelength measurements and must not be confused with frequency measurements.

### 1.1 References

[1.1] A. R. Downie, M. C. Magoon, T. Purcell, and B. Crawford, Jr., The calibration of infrared prism spectrometers, J. Opt. Soc. Am. **43**, 941–951 (1953).
[1.2] Tables of Wavenumbers for the Calibration of Infrared Spectrometers, prepared by the Commission on Molecular Structure and Spectroscopy of IUPAC, Butterworths, Washington (1961).
[1.3] A. R. H Cole, Tables of Wavenumbers for the Calibration of Infrared Spectrometers, 2nd edition, prepared by the Commission on Molecular Structure and Spectroscopy of IUPAC, Pergamon Press, New York (1977).
[1.4] E. K. Plyler, A. Danti, L. R. Blaine, and E. D. Tidwell, Vibration-rotation structure in absorption bands for the calibration of spectrometers from 2 to 16 microns, J. Res. Natl. Bur. Stand. (U.S.) **64A**, 29–48 (1960).
[1.5] K. Narahari Rao, R. V. deVore, and Earle K. Plyler, Wavelength Calculations in the Far infrared (30 to 1000 microns), J. Res. Natl. Bur. Stand. (U.S.) **67A**, 351–358 (1963).
[1.6] K. Narahari Rao, C. J. Humphreys, and D. H. Rank, Wavelength Standards in the Infrared, Academic Press, New York (1966).
[1.7] G. Guelachvili, and K. Narahari Rao, Handbook of Infrared Standards, Academic Press, Inc., San Diego (1986).

## 2. Techniques Used for Infrared Heterodyne Frequency Measurements

### 2.1 Preliminary Considerations

At the present time the only frequency measurements that have been made on infrared absorption spectra of molecules treated in this book are those made in the NIST Boulder Laboratories [5.73],^4^ the Harry Diamond Laboratories [5.88], the University of Lille [5.126], and University of Bonn [5.321]. The measurements of the N_2_O laser transition frequencies made at the National Research Council of Canada [5.183] were also used here. This chapter describes the techniques that have been used in the NIST Boulder frequency measurements in order to familiarize the reader with the techniques that have evolved. We hope that such familiarity will give greater confidence in the accuracy of the results.

The first heterodyne frequency measurements on carbonyl sulfide (OCS) were made in the NIST Boulder Labs. A frequency-stabilized CO_2_ laser served as the local oscillator for these heterodyne measurements. For this chapter, the term local oscillator is reserved for the fixed-frequency oscillator which carries the reference frequency information (near the frequency of the molecular transition to be measured) to the mixer or heterodyne detector. For each OCS measurement, the frequency of a tunable diode laser (TDL) was locked to the peak of a selected (OCS) absorption line with an assigned uncertainty, *δv*_lock_, given by ± 1/2*Δv*_Dopp_/SNR, where Δ*v*_Dopp_ is the full Doppler width at half maximum and SNR is the signal-to-noise ratio of the first derivative lock signal. The frequency of the locked TDL was compared with the frequency of the CO_2_ laser frequency standard by mixing the TDL and CO_2_ laser radiation in a fast (1 GHz, 3 dB bandwidth) HgCdTe detector. The resulting difference-frequency beatnote was measured with the aid of a spectrum analyzer and a marker oscillator (a conventional oscillator whose frequency was tuned to the center of the beatnote). The frequency of the OCS transition was the sum of the CO_2_ laser frequency and the appropriately signed beatnote frequency.

Similar sets of measurements have also been made in which the TDL was heterodyned against a CO laser local oscillator (or transfer oscillator). In these cases the frequency of the CO laser was simultaneously measured relative to a frequency synthesized from combinations of CO_2_ laser standard frequencies [5.94]. In a third type of measurement, a tunable color center laser (CCL) was the local oscillator. In this case, the CCL (which was locked to the transition of interest) was heterodyned directly with a frequency synthesized from CO_2_ laser standards [5.304]. All three techniques are discussed in detail later in this chapter.

The determination of the spectroscopic constants for a particular band of a selected molecule was based on a number of measurements such as those described above. Some preliminary considerations for selection of the particular transitions to be measured were as follows. A review of the set of transitions of a particular band of interest served as a starting point. This set was reduced immediately to those transitions whose frequencies were within 10 GHz (the approximate combined bandpass limit of the HgCdTe detector-rf-amplifier that was used) of the frequency of a CO_2_ laser or a CO laser transition frequency. Those transitions which were blended with nearby transitions from other bands or isotopic species of the molecule of interest were deleted from this subset. An attempt was then made to select (from the candidates available at this point) and measure those transitions which permitted the best determination of the constants. As a minimal set, these included low-*J* transitions in both the P- and R-branches in order to determine the band center, intermediate-*J* transitions for determining the *B* -value, and high-*J* (60 to 100 depending on the particular band strength) transitions for the centrifugal distortion constants. If possible, additional measurements were made in order to cover the entire band with the smallest gaps possible. This served to increase the redundancy and to minimize extrapolated values in the calculated frequencies.

In some instances, it was not possible to realize the above goals. The region of interest often covered 100 cm^−1^ or so and typical TDL frequency coverage (guaranteed by the vendor) was 15 cm^−1^, although many times the coverage turned out to be larger. Within the 15 cm^−1^ region, there were usually holes in coverage and it was necessary to buy the TDLs in pairs. An additional factor which was not within the experimenter’s control concerned frequency holes (regions where the beatnote was not discernible) in the bandpass of the combination of the HgCdTe detector and the rf amplifiers which follow it. These two factors were the final restrictions on the set of measurements which were used to determine the spectroscopic constants for the band.

### 2.2 CO_2_ Laser Standard Frequencies

The 1.25 m long CO_2_ lasers used in these measurements were constructed by the late F. R. Petersen [2.1]. These had gratings for line selection and output mirrors typically coated for 85% transmission. In order to render the output beams nearly parallel, compensated output coupling mirrors were utilized. By compensated, we mean that the anti-reflection coated surface of the mirror was formed with a smaller radius of curvature than the reflecting concave surface. Irises at both ends were available for mode discrimination and control of the power output, which was 1 to 3 W. These lasers were equipped with internal absorption cells (filled to a pressure of 5.3 Pa (40 mTorr) of carbon dioxide) to provide frequency stabilization by the Freed-Javan technique [2.2]. Although some seven isotopic combinations of CO_2_ have been the object of extensive frequency measurements, only three isotopes have been used for the NIST heterodyne frequency measurements. These were ^12^C^16^O_2_ (626), ^13^C^16^O_2_ (636) and ^12^C^18^O_2_ (828). (The shorthand notation used here involves the last digits in the rounded nuclear masses for the three atoms, O, C, and O. For example, the ^16^O^12^C^16^O laser is designated (626).) In addition to the 1.25 m lasers, a 2 m laser with a similar grating and compensated output mirror (but with an external absorption cell for stabilization) was used for high-*J* and hot band transitions. This longer laser has been operated with the three different isotopic gases.

The frequencies of the CO_2_ transitions were initially related to the cesium standard in an NBS experiment published in 1973 (Evenson et al. [2.1]). This was an experiment in which the frequency of the methane stabilized HeNe laser was measured. A new wavelength measurement (Barger and Hall [2.3]) of the same methane transition was concurrently completed. The combination of these two quantities led to a new (at that time) value for the speed of light, 299 792 456.2(1.1) m/s [2.4].

Since 1973 several different international laboratories have repeated this frequency measurement experiment with minor variations and confirmative measurements have led to a new value for the speed of light which is now defined to be 299 792 458 m/s [2.6]. The proposal and expected acceptance of this new definition provided impetus to extend frequency measurements to the visible portion of the spectrum. Major efforts at NIST-Boulder accomplished this objective and experiments were published in 1983 by Pollock et al. [2.7], and by Jennings et al. [2.8].

**Fig. 1:**
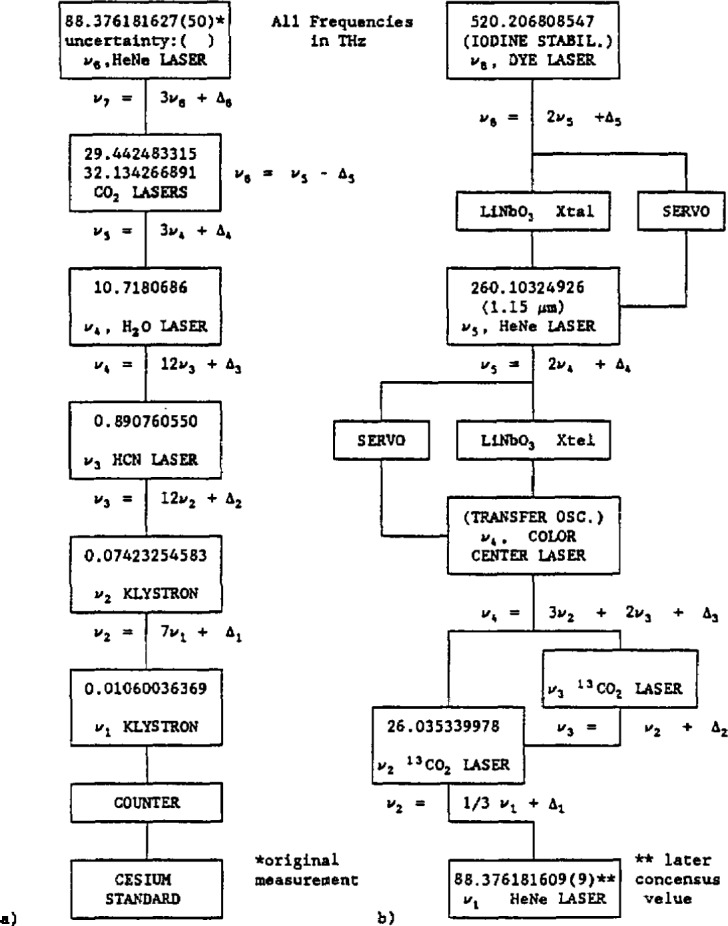
Diagrams of schemes relating CO_2_ frequencies to the cesium standard.

Figure 1b shows a diagram of the chain used to measure the iodine transition at 520 THz (576 nm) [2.7]. The details of this chain are of secondary importance for purposes here, but a sketch will be provided. The starting point was the stabilized HeNe laser at 3.39 *μ*m. A best value for this transition was determined from the most accurate results of the international measurements. The P_1_(50) transition of a stabilized ^13^CO_2_ laser was measured relative to the stabilized HeNe laser, which was assumed to oscillate at the frequency 88 376 181.609±0.009 MHz. The frequency resettability of the CO_2_ laser was reported to be within 5 parts in 10^11^, which is better than the 1 part in 10^10^ uncertainty in the frequency of the HeNe laser. (Petersen et al. [2.9] later used this accurately measured P_1_(50) transition to measure other CO_2_ transitions and to generate improved calibration tables.)

To continue with the sketch, we note that a second ^13^CO_2_ transition P_1_(52) was phase-locked to P_1_(50) by means of a stabilized 62 GHz klystron. The synthesis scheme used to arrive at the CCL frequency is indicated in Fig. 1b. The remainder of the chain was locked from the top down. That is, the second harmonic of the 1.15 *μ*m HeNe laser frequency was locked to the frequency of the dye laser, which was in turn locked to the frequency of the o hyperfine component of the visible (576 nm) ^127^I_2_ 17-1 P(62) transition. The CCL was in turn locked to the frequency of the 1.15 *μ*m HeNe laser. Both the HeNe and the CCL served as transfer oscillators in this scheme. The comparison was made at the CCL-CO_2_ laser point of the chain. In a separate experiment, the HeNe laser was locked to its Lamb dip and that frequency was determined. The ^13^CO_2_ frequency listed in Fig. 1b is the frequency used in the 520 THz determination (it was frequency-offset-locked from a stabilized CO_2_ laser to remove the dither) and is not the frequency of the center of the transition. The values for the two higher frequencies are given in Fig. 1b. Additional details may be found in Ref. [2.7].

The frequencies currently used for the ^12^C^16^O_2_ isotope are based on the most recent values (which made use of the above results) which were published in 1983 by Petersen et al. [2.9]. The stated 1 *σ* uncertainties for the calculated tables based on these measurements are smaller than 5 kHz for the CO_2_ transitions (*J* < 40) which were used in the infrared heterodyne measurements. Subsequent CO_2_ measurements relative to the values of Petersen were made by Freed and coworkers at MIT [2.10]. The uncertainties in the MIT values are also less than 5 kHz for the ^13^C^16^O_2_ transitions (and less than 10 kHz for the ^12^C^18^O_2_ transitions) used here. Note that the 1.25 m CO_2_ lasers used in the TDL measurements were first generation lasers and that the numbers given in Tables 1, 2, and 3 were obtained using second generation lasers. Even if the reproducibility in the laser lock for the older lasers were somewhat poorer (an uncertainty of about 50 kHz was allowed for the realization of the frequencies) it would be of negligible consequence for the results presented here. The transition frequency values (for the three carbon dioxide isotopes) which were used are given in Tables 1, 2, and 3.

Listed in Table 4 are the frequencies and wavenumbers for some transitions of the ^13^CO_2_ hot band (O1^1^1-[11^1^0,03^1^0]_1_) which extend the useful range of standard reference frequencies. These frequencies were determined from NIST measurements in which the 2 m CO_2_ laser and an external reference cell were used. While measurements were made on both the 636 and 626 isotopes [2.11, 2.12], only the 636 frequencies were used for subsequent TDL measurements. The 2 *σ* uncertainties for the transitions used for OCS measurements were less than 80 kHz.

For the measurements in the CO_2_ laser region, the CO_2_ frequencies given in some of our papers were rounded to the nearest 0.1 MHz. However, the full accuracy of the CO_2_ frequencies was retained for calculating or synthesizing the CO frequencies and the CO frequency was then rounded to the nearest 0.1 MHz.

### 2.3 Heterodyne Frequency Measurements with the TDL and CO_2_ Laser (860 to 1120 cm^−1^)

**Fig. 2:**
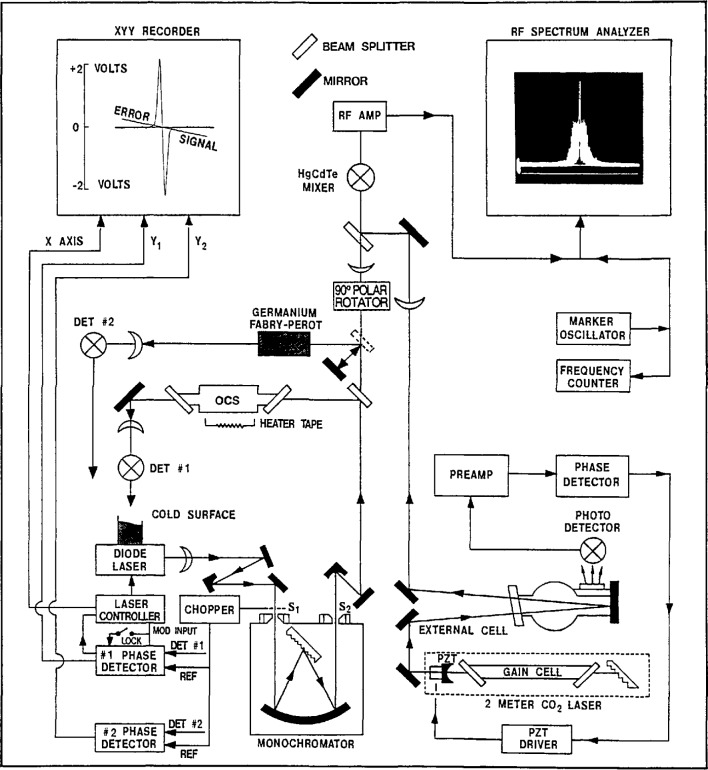
Block diagram of scheme used for heterodyne measurements with a CO_2_ laser.

The measurement procedure has evolved over the course of this work [5.73, 5.83, 5.87, 5.221, 5.230, 5.243]. A brief history of the TDL refrigeration evolution and the details of the apparatus and procedure currently in use will be described here. For considerations involving the TDL, refer to Fig. 2, which is a block diagram of the measurement scheme recently used for some N_2_O measurements [5.243].

The first commercially available TDL spectrometers featured liquid helium Dewars as the refrigeration system. Our initial system used a 4 L helium Dewar. It was necessary to use an assortment of stainless steel shims between the Dewar’s OFHC (oxygen free high conductivity) copper cold surface and the TDL in order to vary the temperature (and operating wavenumber) of the TDL. The use of liquid helium was inconvenient, changing shims was cumbersome, and the resulting temperature cycling of the TDLs was reputed to shorten their lifetime; nevertheless a narrow TDL linewidth was generally observed.

As the demand for TDLs increased, vendors began experiencing difficulties in growing semiconductors that would meet the customers’ specified frequency region while operating in the 4 to 10 K range. The materials problem became more tractable as the temperature constraint was removed by selling to the customers closed-cycle coolers with 10 to 70 K operating capabilities. Soon it became nearly impossible to obtain TDLs operable at helium temperatures. Most of our measurements were made using a closed-cycle cooler. This was an improvement in many areas, however in spite of an isolation scheme, some residual vibrations from the cooler’s piston were transmitted to the TDL, resulting in the famous jitter linewidth which is familiar to all TDL users. The vendor has made two additional isolation improvement schemes available and while these appear worthwhile, they are still not the ultimate solution.

Currently, the NIST liquid helium Dewar has been modified to accommodate a four-laser mounting platform (including the heater coils and temperature sensing diodes) from a closed-cycle cooler. While helium consumption is too high for most of the TDLs available to us, this does offer promise with the recent advent of the higher temperature MBE (molecular beam epitaxy) TDLs. A few of these are now available and operate in the temperature range accessible with liquid nitrogen (70 to 120 K if a heater is available).

Both the Dewar and closed-cycle refrigerator are interchangeable in that they were compatible with the laser control module (current controller) and temperature control system. Both the control module and temperature controller have also been upgraded to reduce current noise and temperature instabilities; these upgrades have proved worthwhile. The particular type of refrigeration is not specified in Fig. 2.

After passing through the AR-coated ZnSe window of the refrigeration stage, the TDL radiation was collimated with an AR-coated *f*/1 lens and directed with a flat mirror and off-axis parabolic mirror into a 0.8 m Ebert-Fastie monochromator. A second off-axis parabolic mirror recollimated the TDL beam after it emerged from the monochromator. A portion of the beam was split off and passed through an absorption cell (containing the molecule of interest, N_2_O in this case) to a detector which was used initially in recording the spectra and later for the TDL locking procedure. The monochromator and a solid 3 in germanium etalon were used to help identify the particular molecular transition of interest. Once the transition had been identified, the kinematic mirror mount directing the TDL beam through the etalon was removed and the TDL beam was then focused on the HgCdTe mixer element. At this time, both the entrance and exit slits were removed from the monochromator, in order to eliminate the fringes or channel spectra which the slits may cause via feedback to the laser.

At this point in the procedure, it was useful to measure the TDL linewidth by heterodyning its output with that from a CO_2_ laser (or CO laser as described in the next section). TDL linewidths of several hundred megahertz are not uncommon at higher currents (higher gains) when viewed for integration times of several seconds. Next, the current was reduced (while increasing the temperature to maintain frequency) until an acceptable (10 to 30 MHz), or at least the narrowest attainable, linewidth was achieved for a measurement.

### 2.4 TDL Locking Procedure and Minimization of Error

Assuming the best conditions, that is, a strong TDL (0.5 mW for example) with a single mode that has a flat power versus wavelength curve, a narrow TDL linewidth, and a well isolated and intense line for a locking reference, we could likely make a measurement with an uncertainty of less than 1 MHz. While these conditions sometimes prevail, more often, they do not.

It is germane to discuss here the TDL locking procedure and some of the ways we have minimized errors that can creep into a measurement. After the chopper was removed from the TDL beam path, the TDL was frequency-modulated at 4.5 kHz and a first derivative scheme was used to lock the TDL frequency. In this procedure, the frequency of the radiation output of the TDL was tuned from well below the molecular transition to well above it, and the resulting derivative signal (including the baseline and the absorption line) was traced by the recorder. (See derivative trace in the xyy recorder box in Fig. 2.) The frequency was returned to the low value and then the recorder signal monitored as the trace was followed to the derivative midpoint for locking. (Note: The derivative trace in Fig. 2 has a line labeled error signal superimposed on it. This error signal was recorded while scanning the TDL current both up and down while the laser was locked with moderate gain. For infinite gain the line becomes horizontal, or zero volts everywhere; for nearly zero gain, the line has a slope near that of the derivative signal. The slope of the error signal may be chosen (by adjusting the loop gain) to any value between these limits. By proper adjustments of the gain value and the TDL current the TDL may be locked (or stabilized) to nonzero values.) A low gain was used in the lock loop and the recorder pen position was monitored during the measurement. One of the reasons for this was that the TDL mode generally was not flat over the region of the line of interest. Rather than locking the TDL to the zero-voltage point, the TDL frequency was locked to a point where the derivative signal crossed the existing baseline derivative. The sources of error to be avoided are not only sloping background (with resultant zero offset in the derivative signal), but also possible instrumental zero offsets from the lock-in amplifier. Both sources become magnified when dealing with weaker TDL modes or low level absorption and a very high sensitivity on the lock-in amplifier, and the procedure outlined above was essential.

Since the background slopes may have either sign and compensation for zero offsets may be either too large or too small, these errors are random when spread over measurements of several different lines. In the case of strong absorption lines and a powerful TDL mode, the difference between the lock point and 0 V was generally negligible compared to other sources of error.

Another obvious source of error in determining the location of the center of the molecular absorption line was noise. In addition to TDL amplitude fluctuations and detector noise, feedback fringes or channel spectra are also included, although some of amplitude noise can be attributable to feedback. Several techniques were used to minimize sources of error. The frequency of the TDL modulation was chosen to be higher than that of the TDL amplitude fluctuations and higher than the upper frequency of typical detector noise. The usual techniques for fringe reduction, including the monochromator slit removal alluded to earlier, tilting of various optical elements in the TDL beam path (particularly the detectors) were all employed. Cells of absorbing gases (for isolation) were placed in the TDL beam path in a few instances although it was not possible for most spectral regions.

### 2.5 Measurement of the Difference Frequency Between the TDL and Gas (CO_2_ or CO) Laser

The portion of the TDL beam passing through the first beam splitter in Fig. 2 has its polarization rotated by 90° to be parallel to the gas laser beam polarization. (The TDL polarization was assumed to be in the plane of the junction; however, a sizeable perpendicular component may also exist.) The TDL beam was then focused (typically, FL = 12.5 cm) through a second beam splitter onto the HgCdTe fast detector or mixer which had an element with an area of 0.1 mm^2^. The 3 dB bandwidth of this detector was 1 GHz. Power from the gas laser was focused with a 40 cm focal length lens and then reflected off the beam splitter (NaCl was chosen to keep the local oscillator power below 10 mW) in such manner as to make the gas laser beam collinear with the TDL beam and to make the beam waists coincide.

After initial observation of the beatnote, the amplitude was maximized by fine adjustment of the focusing lenses. It was also necessary to ascertain that the beatnote observed on the spectrum analyzer was the one of interest. This was particularly relevant when using the CO laser. On some occasions, the first beatnote observed was due to the TDL radiation mixing with that from a nearby unintended CO transition which could not be prevented from lasing along with the CO transition of interest. Mul timode TDLs are also the source of extraneous beatnotes.

Once we determined that the observed beatnote was the one of interest, the TDL frequency was scanned by changing the current and the beatnote was followed on the spectrum analyzer from zero frequency up (or down) to the molecular feature to be measured. The scan rate was reduced to a lower value and the progress of the beatnote carefully monitored relative to the derivative signal. The TDL frequency was then locked to the desired point on the derivative signal which was displayed on the recorder. The beatnote was then averaged with the persistent screen averaging feature of the spectrum analyzer, and a marker oscillator was adjusted to the center of this averaged display. (A representative beat note is shown in the right hand portion of Fig. 2. Here the frequency span of the spectrum analyzer display was 100 MHz.) The marker oscillator frequency was counted and the measurement repeated a number of times (10 to 20, depending on the reproducibility of the measurements).

### 2.6 Minimization of the Difference Frequency Uncertainties

Ideally, the best method to determine the difference frequency between the TDL and the gas laser would be to use an electronic counter. However, the signal-to-noise ratio (S/N) of the beatnote and the frequency modulation associated with the cold head and compressor generally precluded this approach. The next best approach is that described in the preceding paragraph.

The best measurements were those made with a liquid helium Dewar; the beatnote was essentially stationary on the spectrum analyzer. However, the rapid He consumption rates for higher temperature TDLs made this choice impractical as well as inconvenient. When the compressor was used, the beatnote had a jitter linewidth associated with it and its frequency fluctuations made determination of the beatnote center more difficult. A wide variation of jitter linewidths from many different TDLs has been observed over an extended period. The current tuning rates (60 to 1600 MHz/ma) and linewidths due to current noise vary widely from one TDL to another. In a similar fashion the jitter linewidth varies greatly from one laser to the next, due to varying sensitivity to vibrations associated with the compressor/coldhead. Sometimes an apparent jitter linewidth was due to feedback, however this was generally recognized and steps were taken to minimize it. Beatnotes ranging in width from a few megahertz (10 *μ*m TDLs in a liquid helium Dewar) to 60 to 100 MHz (6 *μ*m TDLs in a closed-cycle cooler) were observed during the measurements. However the larger values (in the 5 to 6 *μ*m region) were observed prior to the currently implemented improvements in the vibration isolation system. The most recent approach was to adjust the current modulation for the derivative lock such that the beatnote linewidth was not broadened beyond the jitter linewidth. (This was subject to retention of a suitable S/N for the lock signal.)

The frequency-modulated beatnote was observed at a repetitive rate on the spectrum analyzer. The pulse rate of the closed-cycle cooler was asynchronous with both the modulation rate and the spectrum analyzer sweep rate. As a result, the beatnote observed on the spectrum analyzer made small and slowly varying excursions about an average value. This led to some scatter in the measured value for the beatnote center frequency. Some experiments were conducted to check the compressor-induced fluctuations as a source of systematic error. Typically, 20 measurements were made with the compressor on, and the marker oscillator frequency was adjusted to the resultant average value. The TDL lock point was then rechecked and the compressor was turned off (eliminating the jitter from the beatnote) momentarily. The jitter-free beatnote was observed for a few seconds in this configuration. To date no appreciable deviation of the jitter-free beatnote from the marker oscillator has been observed.

On some occasions, the beatnote envelope was slightly asymmetric. Generally, two different operators have determined the center value and some subjective disagreement was apparent. In a recent set of measurements of 20 transitions the average value of 10 measurements each from two operators varied by 2.5 MHz. This was well within the assigned uncertainty of 7 MHz for the measurement. More often, the average values from different operators agree within a fraction of 1 MHz.

Another difficulty in these measurements was the presence of holes in the frequency coverage of the detector-rf-amplifier combination. In some instances, these frequency holes were associated with connector lengths and their effect could be minimized (by moving the hole to another frequency) by using line stretchers or by changing cables. Some holes were associated with lengths of connecting elements in the detector, and for practical purposes could not be eliminated. In other cases, holes were associated with amplifiers themselves; sometimes they precluded making measurements. In a few instances shallow holes have led to systematic errors. This occurred when only very weak TDL modes were available and the S/N for the beatnote was small (3 to 4 dB for example). Often the beatnote envelope was fairly wide (50 MHz or greater). In cases like these, one side of the beat-note envelope can overlap a hole and the apparent line center will be shifted. This has happened in a few instances but the error was apparent in the fitting process. In these cases, a repetition of the measurement with a different TDL and a much stronger beatnote gave a different and better fitting result. Such holes generally remain at the same frequency and experience has shown which frequency regions to avoid.

### 2.7 Measurements with a CO Laser Transfer Oscillator and CO_2_ Laser Synthesizer

Near the inception of this program, a CO laser stabilization scheme on low pressure CO laser discharges had been demonstrated by Freed [2.13]. More recently, a stabilization scheme using opto-galvanic detection has been reported by Schneider et al. [2.14]; neither of these schemes was operable over the entire range required for our measurements. Some values of CO frequencies in the literature (available when the measurements in this region began) were in error by over 50 MHz. Since the goal of this measurement program was to be able to make measurements with a 3 MHz uncertainty, it was necessary to measure the frequency of the CO laser at the same time that the CO laser-TDL difference frequency measurements were made on N_2_O and OCS transitions. This process required the use of the CO laser as a transfer oscillator, and the CO laser frequency was measured relative to a frequency generated by a CO_2_ laser synthesizer [5.94, 5.221, 2.15].

Two different CO lasers were used in this manner. One was a sealed-off laser which was cooled by flowing alcohol through dry ice and then through a jacket around the discharge tube. This operated over the frequency range from 1600 to 1900 cm^−1^ (corresponding to lower vibrational quantum numbers ranging from *v*″ = 20 to *v*″ = 6). The second CO laser was a flowing gas laser which was cooled by liquid-nitrogen and operated from 1220 to 1600 cm^−1^ (*v* = 36 to *v* = 20). After installation of a shorter wavelength grating and an appropriate output mirror, operation was extended to the 1900 to 2080 cm^−1^ region (*v* = 6 to *v* = 1). Additional details regarding the liquid-nitrogen cooled CO laser may be found in the literature [5.125, 5.224, 5.231].

**Fig. 3:**
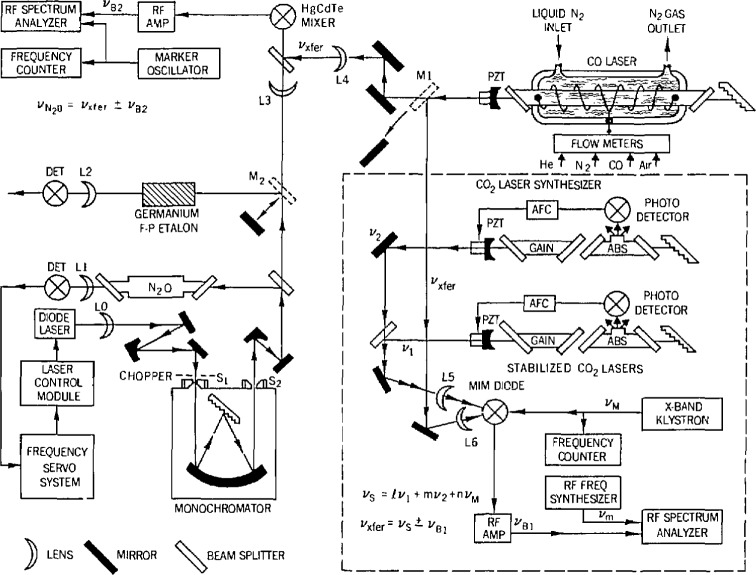
Block diagram of scheme used to make heterodyne frequency measurements with a CO laser. The CO_2_ laser synthesizer is shown in the dashed box.

Figure 3 shows a block diagram for making frequency measurements with the CO laser. The dashed outline shows the kinematically mounted mirror, M_1_, in position for measuring the CO laser frequency relative to the CO_2_ synthesizer, which is shown enclosed in the large dashed box. The synthesizer consists of two stabilized CO_2_ lasers, a phase-locked microwave oscillator and frequency counter, a metal-insulator-metal (MIM) diode, and a combination of an rf amplifier, rf spectrum analyzer, and a 0 to 1.0 GHz rf frequency synthesizer. When radiation from the two CO_2_ lasers and the microwave oscillator were coupled to the MIM diode, currents were generated at a synthesized frequency, *v*_s_, given by

$$v_{s}=lv_{1}+mv_{2}+nv_{M},$$

where *v*_1_ and *v*_2_ were the frequencies of the CO_2_ laser frequency standards, and *v*_M_ was a microwave frequency. The quantities *l*, *m*, and *n* are integers which are allowed both positive and negative values. The quantity (l + |*l*| + |*m*| + |*n*|) is called the mixing order; the synthesized currents generally become weaker as the mixing order is increased. Mixing orders vary from 3 or 4 near 50 THz to 7 or 8 near 38 THz, the frequency at the longest wavelength operation of the CO laser used in these measurements. Typical values might be *l* = 3 or 4, *m* = − 2 or −3, and (with the use of an X-band klystron) n was restricted to 0, ±1, or ±2.

When the CO laser radiation was focused on the MIM diode, an additional current at the CO laser frequency, *v*_CO_, was generated in the diode and it combined with the synthesized frequency, *v*_s_, to produce a difference frequency beatnote at a frequency, *v*_B1_. (The microwave frequency was chosen so that this beatnote was within the 1.2 GHz range of the spectrum analyzer in use.) The beatnote was amplified, displayed on the spectrum analyzer, and its excursion was noted as the CO laser was tuned through its gain bandwidth. The beatnote was positioned at the center of this excursion (a determination of the frequency of the CO transition was a secondary objective) and a marker signal from the rf synthesizer was used to mark this frequency point on the spectrum analyzer. The rf synthesizer reading was then used as the value for *v*_B1_ and the CO laser (which was not locked) was periodically readjusted to return the beatnote to the assigned frequency.

The frequency of the CO laser (transfer oscillator) was then

$$v_{\mathrm{CO}}=v_{\text{transfer}}=v_{s}\pm v_{B1}.$$

The full accuracy of CO_2_ frequencies was used for the *v*_s_ calculation. An uncertainty of 0.3 MHz (which includes allowance for drift between readjustments) in the transfer oscillator was included in the measurement uncertainty of the molecular transition, which was given by

$$v_{\mathrm{mol}}=v_{\text{transfer}}\pm v_{B2},$$

where *v*_B2_ was the beatnote between the TDL and the transfer oscillator. The transfer oscillator frequency and beatnote frequency are both rounded to the nearest 0.1 MHz. The main uncertainty was again due to the TDL linewidth which was discussed in the early part of the chapter.

The most recent advance in making heterodyne frequency measurements with TDLs involves a computer-controlled, frequency offset-locking (CC-FOL) scheme. Freed et al. [2.16] demonstrated the use of a frequency offset lock combined with a frequency synthesizer to control the output frequency of the TDL. We have combined that technique with the scanning and data-logging technology used in this laboratory for other measurements [2.17–2.19] to obtain accurate data on d*v*/d*P*, the pressure-induced frequency shifts, in the rovibrational spectrum of OCS. The potential for better absolute frequency measurements was also demonstrated.

**Fig. 4:**
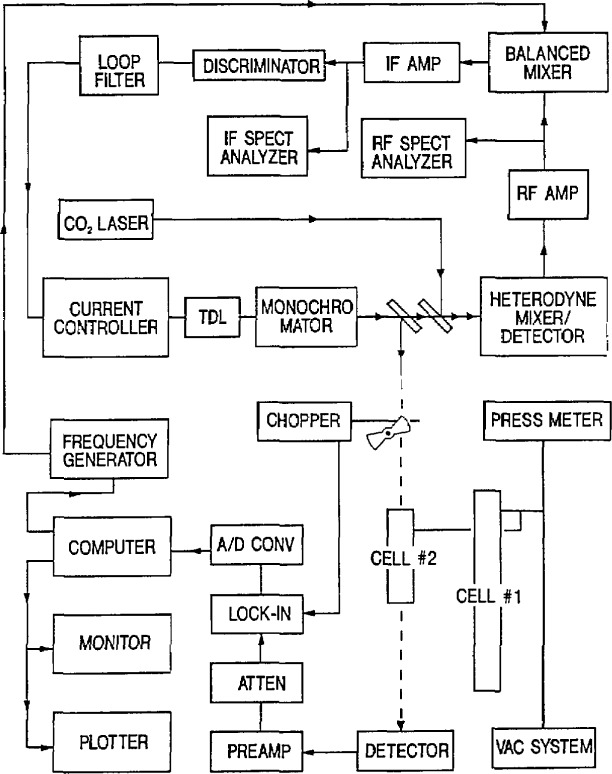
Block diagram of a computer controlled frequency offset-locked spectrometer.

Figure 4 shows a block diagram of the apparatus used for this type of measurement. The output beams from a TDL and a CO_2_ laser frequency standard were focused with separate lenses and then combined with a ZnSe beam splitter and directed to a HgCdTe heterodyne mixer/detector which produced a beatnote at the difference frequency, *v*_B_, between the two lasers. The beatnote was amplified in an rf amplifier and displayed on an rf spectrum analyzer. A balanced mixer was used to down-convert the beatnote at frequency *v*_B_ to a nominal 160 MHz, the region of operation of the IF amplifier and discriminator. The beatnote was fed to one input arm of the balanced mixer, and the output of the sweepable frequency generator, at frequency *v*_sw_, was fed to the other input arm (the local oscillator arm). The frequency *v*_sw_ was adjusted such that |*v*_sw_−*v*_B_| was nominally 160 MHz, and this resulting output signal was fed to the discriminator which had a sensitivity of 0.1 V/MHz and an 80 MHz bandwidth.

After the switch in the loop filter was closed, the discriminator-based locking loop adjusted the TDL frequency to insure that the beatnote *v*_B_ was locked at a frequency *v*_DO_ away from *v*_sw_ That is,

$$v_{\mathrm{DO}}=\left|v_{\mathrm{sw}}-v_{B}\right|,$$

where *v*_DO_ is close but (due to the presence of various zero offsets in the locking loop) not necessarily equal to 160 MHz. For frequency shift and lineshape measurements, the important point is that the frequency *v*_DO_ must remain fixed, whatever value it assumes. If a frequency measurement is the objective, it becomes necessary to measure *v*_DO_. Frequency of the CCFOL TDL is then given by

$$v_{\mathrm{TDL}}\pm v_{{\mathrm{CO}}_{2}}\pm v_{\mathrm{sw}}\pm v_{\mathrm{DO}}.$$

Two frequency measurements were made with an uncertainty of ± 2 MHz, which was almost entirely due to the TDL linewidth. By narrowing the TDL linewidth with a faster loop filter, one should be able to use an electronic counter to measure *v*_DO_ and make measurements with uncertainties the order of 0.2 MHz.

We have restricted our initial experiments to those OCS transitions which lie within 2000 MHz of a CO_2_ laser transition because the lock loop requires a beatnote with a good *S*/*N* and the beatnote signal decreases with increasing frequency. This is also the band limit of our most convenient rf amplifier. We chose from the available TDLs those with sufficient power to give a beatnote with a *S*/*N* of about 30 dB. For the present measurements a 400 ms integration time was used and 640 points were recorded in each direction. Recording in both directions is a good way to cancel certain types of systematic errors. Generally only one round trip pass was made per measurement but as many passes could be made as required to give a good *S*/*N.*

A large number of measurements have been made with the older technique and these have been combined with FTS measurements, particularly the high quality measurements made recently. As the situation stands now good molecular constants exist, with the band centers currently having the largest uncertainties. The number of measurements left to be made is a relatively small number of high quality. The best approach is to make sub-Doppler or saturated measurements, however, the accidental overlaps required are rather infrequent. We believe the CCFOL approach is the next best option and several strategic overlaps occur in the 2 GHz range.

### 2.8 Measurements with the Color Center Laser

Pollock et al. [5.219] used a color center laser (CCL) to perform a set of experiments on N_2_O. A brief description of their work and some related work concludes the summary of heterodyne techniques. It is of interest to compare and contrast some of the salient features of the TDL and the CCL. The tuning range of a TDL mode was 15 to 30 GHz; that of the CCL was less than 1 GHz. The linewidth of the TDL in the best instances was a few MHz, that of the CCL was 10 kHz. Perhaps the most important feature of the CCL was a large power output (in excess of 10 mW), which along with its beam quality permitted a direct coupling of the CCL output to the MIM diode, and subsequent synthesis measurements without a transfer oscillator. This relatively large power and narrow linewidth made it an ideal tool to use for some saturated absorption measurements by Pollock et al. on CO [5.304]. Sub-Doppler measurements could not be made on the N_2_O band studied, and uncertainties of 4 to 8 MHz (at 130 to 140 THz) were reported. This uncertainty was due to the uncertainty in locating the center of the transition and was due in part to the small free spectral range of the tuning element of the CCL, which frequently prevented us from sweeping over the entire line and was also insufficient to sweep far enough on either side of an absorption line to determine a background slope.

**Fig. 5:**
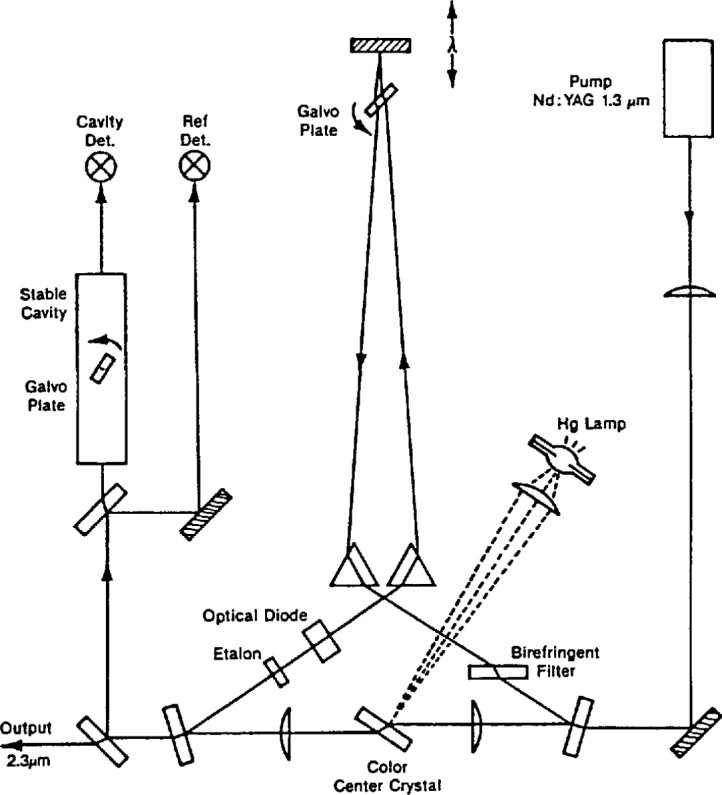
Block diagram of color center laser in ring configuration.

Shown in Fig. 5 is a block diagram of the ring configuration color center laser developed by Pollock and Jennings [2.20]. The lasing entity in the lithium-doped potassium chloride crystal was an (F^+^_2_)_A_ center. The centers were optically pumped with the 3 W power output from an Nd:YAG laser operating in a TEM_00_ mode at 1.3 μm. These color centers were continuously replenished by uv radiation from an Hg lamp.

Two Brewster’s angle sapphire prisms, a single plate birefringent filter and one etalon comprised the tuning elements. The ring was constrained to operate in a unidirectional manner by an optical diode consisting of an AR-coated YIG plate in a 0.1 T magnetic field and a Brewster-cut quartz plate reciprocal rotator. One portion of the output radiation was used for stabilization to the side of a fringe in a passively stabilized optical cavity. The cavity was scanned by tuning a Galvo plate inside this reference cavity. Corrections were applied to the Galvo plate (slow) and to the PZT driving the tuning mirror (fast) in the laser resonator; this narrowed the CCL linewidth to 10 kHz. A second portion of the beam was split off and sent through a cell containing CO. The Galvo plate in the reference cavity was modulated at 7 kHz and slowly scanned to observe the first derivative signal, which was used to lock the CCL to the CO lines of interest.

A third portion of the CCL radiation was directed to the CO_2_ synthesizer (more specifically the MIM diode portion) for a simultaneous measurement of the CO frequency. Typical synthesis schemes used 5*v*_1_, 4*v*_1_ + *v*_2_, or 3*v*_1_ *+ v*_2_, where *v*_1_ and *v*_2_ are different CO_2_ laser frequencies. No microwave oscillators were required, and the *v*_B1_ type beatnotes fell within 2 GHz. In contrast to the TDL measurements, the measurement uncertainty was entirely the uncertainty in locating the center of the absorption line.

### 2.9 References

1. [2.1] K. M. Evenson, J. S. Wells, F. R. Petersen, B. L. Danielson, and G. W. Day, Accurate frequencies of molecular transitions used in laser stabilization: the 3.39-μm transition in CH_4_ and the 9.33- and 10.18-μm transitions in CO_2_, Appl. Phys. Lett. **22**, 192–196 (1973).
2. [2.2] C. Freed and A. Javan, Standing-wave saturation resonances in the CO_2_ 10.6-μ transitions observed in a low pressure room temperature absorber gas, Appl. Phys. Lett. **17**, 53–56 (1970).
3. [2.3] R. L. Barger and J. L. Hall, Wavelength of the 3.39-μm laser saturated absorption line of methane, Appl. Phys. Lett. **22**, 196–199 (1973).
4. [2.4] K. M. Evenson, J. S. Wells, F. R. Petersen, B. L. Danielson, G. W. Day, R. L. Barger, and J. L. Hall, Speed of light from direct frequency and wavelength measurements of the methane-stabilized laser, Phys. Rev. Lett. **29**, 1346–1349 (1972).
5. [2.5] F. R. Petersen, D. G. McDonald, J. D. Cupp, and B. L. Danielson, Accurate rotational constants, frequencies and wavelengths from ^12^C^16^O_2_ lasers stabilized by saturated absorption, Laser Spectroscopy, (ed. Brewer and Mooradian) Plenum Press, pp. 555–569 (1974).
6. [2.6] D. A. Jennings, R. E. Drullinger, K. M. Evenson, C. R. Pollock, and J. S. Wells, The continuity of the meter: the redefinition of the meter and the speed of light, J. Res. Natl. Bur. Stand. (U.S.) **92**, 11–16 (1987).
7. [2.7] C. R. Pollock, D. A. Jennings, F. R. Petersen, J. S. Wells, R. E. Drullinger, E. C. Beaty, and K. M. Evenson, Direct frequency measurements of transitions at 520 THz (576 nm) in iodine and 260 THz (1.15 μm) in neon, Opt. Lett. 3, 133–135 (1983).
8. [2.8] D. A. Jennings, C. R. Pollock, F. R. Petersen, R. E. Drullinger, K. M. Evenson, J. S. Wells, J. L. Hall, and H. P. Layer, Direct measurement of the I_2_ stabilized He-Ne 473 THz (633 nm) laser, Opt. Lett. **3**, 136–138 (1983).
9. [2.9] F. R. Petersen, E. C. Beaty, and C. R. Pollock, Improved rovibrational constants and frequency tables for the normal laser bands of ^12^C^16^O_2_, J. Mol. Spectrosc. **102**, 112–122 (1983).
10. [2.10] L. C. Bradley, K. L. Soohoo, and C. Freed, Absolute frequencies of lasing transitions in nine CO_2_ isotopic species, IEEE J. Quant. Elect. **QE-22**, 234–267 (1986).
11. [2.11] F. R. Petersen, J. S. Wells, A. G. Maki, and K. J. Siemsen, Heterodyne frequency measurements of ^13^CO_2_ laser hot band transitions, Appl. Opt. **20**, 3635–3640 (1981).
12. [2.12] F. R. Petersen, J. S. Wells, K. J. Sicmsen, A. M. Robinson, and A. G. Maki, Heterodyne frequency measurements and analysis of ^12^C^16^O_2_ laser hot band transitions, J. Mol. Spectrosc. **105**, 324–330 (1984).
13. [2.13] C. Freed and H. A. Haus, Lamb dip in CO lasers, IEEE J. Quant. Elect. **QE-9**, 219–226 (1973).
14. [2.14] M. Schneider, A. Hinz, A. Groh, K. M. Evenson, and W. Urban, CO laser stabilization using the optogalvanic Lamb-dip, Appl. Phys. **B44**, 241–245 (1987).
15. [2.15] J. S. Wells, D. A. Jennings, and A. G. Maki, Improved deuterium bromide 1-0 band molecular constants from heterodyne frequency measurements, J. Mol. Spectrosc. **107**, 48–61 (1984).
16. [2.16] C. Freed, J. W. Bielinski, and W. Lo, Programable secondary frequency standard based infrared synthesizer using tunable lead-salt lasers, Proc. SPIE **438**, 119–124 (1984).
17. [2.17] K. M. Evenson, D. A. Jennings, and F. R. Petersen, Tunable far-infrared spectroscopy, Appl. Phys. Lett. **44**, 576–578 (1984).
18. [2.18] D. A. Jennings, The generation of coherent tunable far infrared radiation, Appl. Phys. **B48**, 311–313 (1989).
19. [2.19] D. A. Jennings, K. M. Evenson, M. D. Vanek, I. G. Nolt, J. V. Radostitz, and K. V. Chance, Air- and oxygen-broadening coefficients for the O_2_ rotational line at 60.46 cm^−1^, Geophys. Res. Lett. **14**, 722–725 (1987) and “Correction: Air- and oxygen-broadening coefficients for the O_2_ rotational line at 60.46 cm^−1^, Geophys. Res. Lett. **14**, 981 (1987).
20. [2.20] C. R. Pollock and D. A. Jennings, High Power cw Laser Operation Using (F^+^_2_)_A_ Color Centers, Appl. Phys. **B28**, 308–309 (1982).

## 3. Formulas and Data Sources Used to Prepare the Tables

### 3.1 Expressions Used for Fitting the Frequency Data and for Calculating the Transition Wavenumbers

For diatomic molecules and for linear triatomic molecules in ^1^*∑* electronic states the energy levels are generally given by

$$E_{vJI}=G_{v}+B_{v}J\left(J+1\right)-D_{v}{\left[J\left(J+1\right)-l^{2}\right]}^{2}+H_{v}{\left[J\left(J+1\right)-l^{2}\right]}^{3}+L_{v}{\left[J\left(J+1\right)-l^{2}\right]}^{4}+\text{higher}\;\text{terms},$$ (3.1)

where *J* is the quantum number for overall rotational angular momentum and *l* is the quantum number for vibrational angular momentum. Diatomic molecules have no vibrational angular momentum; that is, *l* = 0. In this work no higher order terms were needed and even the *H*_v_ and L_v_ terms were either poorly determined or not determinable.

For diatomic molecules, an alternative formulation is often given for the energy levels,

$$E_{vJ}=\sum_{ij}Y_{ij}{\left(\nu +1/2\right)}^{i}{\left[J\left(J+1\right)\right]}^{j}.$$ (3.2)

Dunham [3.1] has related the *Y_ij_* constants to the potential function of a diatomic molecule in a ^1^*∑* state. Most of the papers reporting constants for CO use Eq. (3.2). The ground state of the NO molecule is a ^2^*∏* state and is treated differently [5.371].

Transitional frequencies, *v*_calc_, are calculated as differences between energy levels

$$\nu_{\mathrm{calc}}=E_{vJl}\prime -{E_{vJl}}^{\prime \prime }.$$ (3.3)

In this book the band centers, *v*_0_, are defined by

$$\nu_{0}=G_{v}^{\prime }-G_{v}^{\prime \prime }.$$ (3.4)

For linear triatomic molecules two types of perturbations are commonly encountered that affect the importance of higher order terms in Eq. (3.1), *l*-type resonance and Fermi resonance. Because it has a large effect on the centrifugal distortion constants, *l*-type resonance was treated explicitly in the analysis of the OCS and N_2_O spectra. Both *l*-type doubling and *l*-type resonance are manifestations of the same matrix element that couples levels that differ only in the value of the *l* quantum number, where *l* is treated as a signed quantum number. In this book, both effects are treated under the general title of *l*-type resonance. If the bending vibrational quantum number, *v*_2_, is greater than zero, *l*-type resonance will be present and is usually noticeable.

When *v*_2_ *≠* 0, the *l*-type resonance was taken into account by diagonalizing the energy matrix which includes the matrix elements coupling *l* levels with *l* ± 2 levels. The form of these matrices has been described in Refs. [5.120] and [3.2] but we shall repeat that description for a specific case.

For *v*_2_ = 3, there are four possible values of *l*, *l* = 3, *l* = 1, *l* = −1, and *l* = −3. The *l*-doubling constant, *q*_v_, represented by

$$q_{v}={q_{v}}^{0}-q_{vJ}J\left(J+1\right)+q_{v\mathrm{JJ}}J^{2}{\left(J+1\right)}^{2},$$ (3.5)

couples these levels through the matrix element

$$W_{l-2,l}=W_{l,l-2}=<v,l\left|H\right|v,l-2>=1/4q_{v}{\left\{\left[v_{2}+l\right]\left[v_{2}-l+2\right]\times \left[J\left(J+1\right)-l\left(l-1\right)\right]\left[J\left(J+1\right)-\left(l-1\right)\left(l-2\right)\right]\right\}}^{1/2}.$$ (3.6)

For each *J* level the form of the energy matrix for v_2_ = 3 is

$$\left|\begin{array}{cccc} E^{0}\left(v,l=3\right) & W_{3,1} & 0 & 0\\ W_{1,3} & E^{0}\left(v,l=1\right) & W_{1,-1} & 0\\ 0 & W_{-1,1} & E^{0}\left(v,l=-1\right) & W_{-1,-3}\\ 0 & 0 & W_{-3,-1} & E^{0}\left(v,l=-3\right) \end{array}\right|.$$ (3.7)

Here, the matrix elements are given by Eq. (3.1) (where *E*_v_*_Jl_* =* E*^0^) and Eq. (3.6). *J* = 0 is not allowed and for *J* < 3 only the central two-by-two matrix is allowed. Higher order terms coupling *l* and *l ±* 4 levels are sometimes important but were not necessary for the present calculations.

In general the *l*-type resonance calculation requires the use of a matrix of dimension *v*_2_ +1 by *v*_2_ + l. Since this is a nonlinear system, a nonlinear least-squares fitting technique was needed to fit the experimental data to determine the best constants, as explained later.

Most other workers have only used Eq. (3.1) to fit the data of OCS and N_2_O. That has the effect of absorbing the *l*-type resonance into the effective *B*_v_, *D*_v_, and *H*_v_ values. While such a treatment is quite reasonable, the effective values of the higher order constants are quite different from the ground state values, and the level at which Eq. (3.1) is truncated has an important effect on both interpolation and extrapolation. By treating the *l*-type resonance explicitly, we bring the effective values for *D*_v_ and *H*_v_ much closer to the ground state values. This model gives a better approximation to the true Hamiltonian than the model that only uses Eq. (3.1). This improvement in the model used for fitting the data improves the reliability of the least-squares fits and gives more accurate uncertainties for the calculated transition frequencies.

For each value of |*l*| (except *l* = 0) the states with *v*_2_ > 0 are split into *e* and *f* components. For OCS and N_2_O the *l* = 0 states (^1^*∑^+^* states) always have the same symmetry (or parity) as the *e* states. These *e* and *f* components have been assigned in accordance with the convention established by Brown et al. [3.3]. That convention leads to the selection rules:

$$\begin{array}{l} \Delta J=0,e↔f\\ \Delta J=\pm 1,e↔e,\;\text{and}\;f↔f \end{array}$$

for electric dipole transitions. These selection rules are obeyed even when the normal rule, *Δl* = 0, ± 1, is broken because perturbations always connect *e* to *e* and *f* to *f*.

All of the *l*-type resonance energy matrices, like Eq. (3.7), may be factored into two submatrices which represent the e levels in one case and the *f* levels in the other case. We have used the full matrix, as indicated by Eq. (3.7), rather than a factored form, because it is more convenient for obtaining the eigenvectors needed to calculate the intensities of the transitions.

The present analysis ignores the Fermi resonance that couples the levels (*v*_1_, *v*_2_ + 2,*l*, *v*_3_ − 1,*J*) and (*v*_1_, *v*_2_,*l, v*_3_,*J*) of OCS and N_2_O. In OCS the unperturbed Fermi resonance levels are far apart, so there is very little change in the resonance across a band. This results in only small changes in the effective values of *D*_v_, *H*_v_, and *L*_v_. Such small changes can be accommodated by Eq. (3.1) without affecting either the accuracy of the least-squares fits or the accuracy of the calculated values. In N_2_O the Fermi resonance is expected to be more important but again the effective values of *D*_v_, *H*_v_, and *L*_v_ are only slightly changed from the unperturbed values.

Since the Fermi resonance coupling is different for different values of |*l*|, it gives rise to different effective values of the constants *q*_v_, *B*_v_, etc. for levels that differ only in the value of |*l*|. Consequently, in Eq. (3.7) one must use two values for *B*_v_, one for the |*l*| = 1 states and one for the |*l*| = 3 states. Similarly, two values are needed for *D*_v_, *H*_v_, and *L*_v_. There will also be two different off-diagonal coupling constants, *q*_v_, in Eq. (3.7), one for the *W*_3,1_ *W*_1,3_, W_−1_, _−3_, and *W*_−3_, _−1_ terms and a slightly different one for the *W*_1_, _−1_ and *W*_−1_,_1_ terms. However, these small differences in *q*_v_ are difficult to separate from the differences in *B*_v_ and *D*_v_. Consequently, we have been forced to use a single value of *q*_v_ for a given vibrational state irrespective of differences in the value of the *l* quantum number.

In analyzing the spectral data to get the rovibrational constants for calculating the most accurate transition frequencies of OCS and N_2_O, a large body of data on many different types of transitions was fit. Because of the form of the energy matrix for *l*-type resonance, it was not possible to use a linear least-squares technique to fit the data. Instead, an iterative nonlinear least-squares fitting procedure was used. In this procedure it was necessary to approximate the derivative of the transition frequency with respect to each constant by applying the technique described by Rowe and Wilson [3.4].

A similar nonlinear least-squares fitting procedure was used for NO but the energy matrix was somewhat different from Eq. (3.7). For a complete description of the energy matrix for NO one should refer to the work of Hinz et al. [5.371].

In order to calculate the statistical uncertainties in the calculated wavenumbers given in these tables it was necessary to use the variance-covariance matrix, given by the least-squares analysis, and the derivative of the transition frequency with respect to each constant. The uncertainty, or estimated standard error, given by *σ*(*v*) was then determined by the double summation,

$$\sigma \left(v\right)=\sum_{i}\sum_{j}V_{\mathrm{ij}}\left(\partial v/\partial c_{i}\right)\left(\partial v/\partial c_{j}\right),$$ (3.8)

where *V_ij_* is a particular element of the variance-covariance matrix and *∂v*/*∂c_i_* and *∂v*/*∂c_j_* are the derivatives of the transition frequency with respect to the rovibrational constants *c_i_*, and *c_j_* respectively.

### 3.2 Data Sources Used for Fitting the Frequency Data and for Calculating the Transition Wavenumbers

#### 3.2.1 OCS

All of the OCS transitions to be used for calibration (shown with an asterisk in the atlas) were calculated by means of constants and a variance-covariance matrix given by a single least-squares fit that included all of the frequency measurements given in the literature. The equations used in this fit were described in the preceding section. In this section we indicate what references provided the data that went into that fit and give a few more details about the fit and the selection of data.

The rotational spectrum of OCS has been extensively studied by microwave and sub-millimeter wave techniques. These measurements use frequency techniques for calibration and have uncertainties on the order of ± 0.05 MHz and in some cases even smaller uncertainties. Such measurements are blessed with small line widths and are made at low pressures which contribute to the accuracy of the measurements. The three most abundant isotopic species of OCS have no fine structure due to quadrupole effects.

Some microwave measurements [5.43, 5.79] extend to fairly high *J* values so they are able to give accurate values for *B*_v_ and *D*_v_. In addition, the heterodyne measurements made by Vanek et al. [5.124] on high-*J* transitions were used in the analysis. Although there are a great many measurements of rotational transitions for the lower vibrational states, Bogey and Bauer [5.78] and Tanaka et al. [5.98] have given measurements of rotational transitions for fairly high vibrational states, up to 4100 cm^−1^. Some transitions show the splitting due to 1-type resonance and for a few vibrational states, 01^1^0 [5.32, 5.47], 02^2^0 [5.54], and 03^1^0 [5.54], the transitions between split levels have been observed.

Altogether 333 frequency measurements of rotational transitions, taken from the above references as well as from Refs. [5.5, 5.29, 5.30, 5.37, 5.41, 5.48, 5.55, 5.66, 5.119], were included in the least-squares fit that determined the rovibrational constants given in Tables 5 and 6. When possible these measurements were given uncertainties suggested in the original papers. In some cases the uncertainty was estimated by us, based on other work from that time or from that laboratory, or based on the goodness of the fit.

With three exceptions, all of the infrared heterodyne frequency measurements came from a series of papers from the same laboratory at the National Institute of Standards and Technology in Boulder, Colorado [5.73, 5.83, 5.87, 5.94, 5.107, 5.120–5.122, 5.125, 5.129]. The exceptions are the measurements on the 02^0^0-00^0^0 band and accompanying hot bands made in the Harry Diamond Laboratory by Sattler et al. [5.88], the measurements in the same frequency region made at the University of Lille by Fayt et al. [5.126], and the preliminary measurements from the University of Bonn [5.137a]. From the root-mean-square (rms) deviations of the NIST measurements it was obvious that their assigned uncertainties were too large by approximately a factor of two. This reflected caution in allowing for systematic errors which would not be revealed by the least-squares analysis.

An extensive set of laser-Stark resonance measurements have been made by Fayt and others [5.105, 5.106, 5.111, 5.112] and by Tanaka et al. [5.71, 5.97]. This is a type of frequency measurement that should be quite accurate but we have not included those data in this least-squares fit as they would introduce the additional complications of determining dipole-moment functions and assessing the accuracy of the electric field measurements. In the case of measurements using CO laser transitions there is an additional uncertainty in the laser frequency.

In order to determine the most accurate centrifugal distortion constants, some of the better diode laser and FTS measurements were included in the least-squares fits. For the most part the diode laser measurements given in Refs. [5.83, 5.88, 5.94] were calibrated with the heterodyne measurements and probably had systematic errors much smaller than the dispersion shown by the least-squares fit.

Except for the weakest transitions, the FTS measurements were more precise than any of the other infrared measurements. The high precision, however, does not necessarily imply high accuracy. The FTS data used in the analysis for these tables were taken from Refs. [5.75, 5.101, 5.102, 5.120, 5.122, 5.128, 5.132, 5.135, 5.136] or from private communication with the authors of those papers in the cases where the original data were not published.

The FTS measurements were given uncertainties equal to the rms deviations of the fits on a band-by-band basis. In order to keep the FTS measurements from affecting the determination of the vibrational energy levels, they were fitted to the same rotational constants as the other data, but to different band centers. All of the recommended calibration data were based on vibrational energy levels determined only from frequency measurements, not from FTS or ordinary diode-laser measurements.

For the less abundant isotopomers of OCS many of the above papers plus a few additional papers [5.5, 5.52, 5.82, 5.84] give microwave and sub-millimeter wave measurements of rotational transitions. Some of the infrared heterodyne measurements also included transitions for the less abundant isotopomers of OCS [5.73, 5.87, 5.94, 5.120, 5.122, 5.125]. A few transitions of ^16^O^13^C^32^S and ^16^O^12^C^34^S have enough frequency measurements to warrant being considered as possible calibration transitions. In most cases, however, transitions of the rarer isotopomers are only included in the atlas to help in identifying the other, more useful transitions.

A good many wavelength measurements have been made on the less abundant isotopomers, and they can be found in the bibliography, Sec. 5. The most important sources of information on infrared measurements of the less abundant species are Refs. [5.45, 5.65, 5.76, 5.101, 5.109, 5.111, 5.128].

#### 3.2.2 N_2_O

The microwave data on N_2_O are not so extensive as for OCS but a great many measurements are still available. The early work of Pearson et al. [5.161] and of Lafferty and Lide [5.156] were very useful as were other early measurements given in the review by Lovas [5.5], namely the data given in Refs. [5.138, 5.139, 5.146, 5.148, 5.159, 5.162]. The *v*_1_ rotational transitions given by Bogey [5.186] and the high-*J* transitions given by Andreev et al. [5.194], by Burenin et al. [5.179], and by Vanek et al. [5.242] were of particular value for better determining the centrifugal distortion contribution to the line positions.

All of the heterodyne measurements involving N_2_O have come from two laboratories, NRC in Canada, and NIST in the United States. The only saturated absorption measurements were those of Whitford et al. [5.183] on the laser transitions, 10^0^0-00^0^1, near 930 cm^−1^. The other infrared heterodyne measurements were made by Wells and co-workers in a series of papers, Refs. [5.219, 5.221, 5.224, 5.230, 5.231, 5.241, 5.243].

There have been a great many measurements on infrared bands of N_2_O using either grating instruments or, more recently, FTS instruments. Some of the more important measurements which were used in the least squares refinement of the constants, but did not contribute to the band centers for the recommended calibration lines, were given in Refs. [5.191–5.193, 5.213, 5.215, 5.220, 5.225, 5.229].

All these data were fit in the same way as was done for OCS. The constants given by the least-squares fit are given in Tables 7 and 8.

For N_2_O the Fermi resonance is much more important than for OCS; nevertheless, the Fermi resonance was ignored in the fits and only the *l*-type resonance was included in the analysis. For some levels the Fermi resonance causes an effective centrifugal distortion quite different from that of the ground state.

In order to show the position of some of the hot band lines in the spectra, it was necessary to use constants for some levels not included in Tables 7 and 8. The wavenumbers for those lines were calculated by taking the constants for the upper state reported in Refs. [5.191, 5.213, 5.215, 5.229].

For the less abundant isotopic species, the microwave data given in Refs. [5.194, 5.227] were used in preparing these tables. One paper has reported heterodyne frequency measurements in the infrared for ^15^N^14^N^16^O and ^14^N^15^N^16^O [5.231]. For the most part the data for the rarer isotopic species were taken from Refs. [1.7, 5.173, 5.184, 5.192, 5.193, 5.213, 5.215, 5.225, 5.229].

#### 3.2.3 CS_2_

Since carbon disulfide (CS_2_) is a symmetric linear molecule, it is nonpolar and has no microwave spectrum. There are, however, several high resolution infrared studies of its spectrum in the 1450 to 1550 cm^−1^ region [5.383, 5.386, 5.389] in addition to the heterodyne measurements made by Wells et al. [5.390]. A number of other measurements have been made on CS_2_ so that the ground state constants, *B*_0_ and *D*_0_, are quite well determined for both the ^12^C^32^S_2_ and ^13^C^32^S_2_ isotopic species.

The analysis of the V_3_ band of CS_2_ is uncomplicated by either Fermi resonance or *l*-type resonance. The analysis used Eqs. (3.1)–(3.3) as described in detail by Wells et al. [5.390]. The recommended calibration frequencies are based on the constants given in their paper. Only the 00^0^1-00^0^0 transitions of ^12^C^32^S_2_ and ^13^C^32^S_2_ should be used for calibration. The other line wavenumbers given in the tables are for identification purposes and to show how close some weaker lines may be to the calibration lines. The wavenumbers of the other lines were calculated from the constants given by Winther et al. [5.389] and may be in error by as much as 0.01 cm^−1^. In the spectral maps some lines may not be shown in the region below 1505 cm^−1^ because they arise from transitions not included in the data base.

#### 3.2.4 CO

The carbon monoxide (CO) wavenumbers for the calibration lines given in the atlas were calculated from the constants given in Table 9 and based on Eqs. (3.2) and (3.3). Only the wavenumbers given for the ^12^C^16^O molecule were determined adequately by frequency measurements and so they are the only wavenumbers that should be used for frequency (wavenumber) calibration. There are a few frequency measurements for the other isotopomers, but not enough to provide good calibration.

For the ^12^C^16^O molecule the ground state constants are primarily based on the sub-millimeter wave measurements given by Gordy and Cowan [5.250], Rosenblum et al. [5.253], and Helminger et al. [5.266] and on the far-infrared heterodyne measurements of Nolt et al. [5.309] and Varberg and Evenson [5.320]. Also included in the fit were microwave measurements of the *J* = 1←0 transitions in the first two vibrationally excited states as reported by Dixon [5.288]. Aside from these two measurements the upper state constants are based primarily on the heterodyne measurements of the 1-0 band given by Schneider et al. [5.314] and by Maki et al. [5.316] and the 2-0 band given by Pollock et al. [5.304]. Also used was one sub-Doppler measurement of the 1-0 band communicated to us by Urban [5.321].

Other data included in the analysis to determine the best constants were some heterodyne laser measurements given by Schneider et al. [5.313] and some FTS measurements from Guelachvili et al. [5.289, 5.301, 5.307] and Brown and Toth [5.306]. The FTS measurements were used to help determine the best centrifugal distortion constants for the *v* = 1, 2, and 3 states. Only heterodyne frequency measurements were used to determine the vibrational frequencies.

The analysis of the CO data was carried out in the same way as described by Maki et al. [5.316] except that new data have been included [5.320, 5.321]. In order to avoid the possibility of problems with the potential function model, the lowest order constants, *Y*_10_, *Y*_20_, *Y*_01_, *Y*_11_, *Y*_21_, *Y*_02_, *Y*_12_, and *Y*_03_ were fit to data for only the *v* = 0, 1, and 2 states. The other constants were constrained to values given by earlier fits which included data for higher vibrational states, as described by Schneider et al. [5.314].

The uncertainties given for the calibration wavenumbers are based on the variance-covariance matrix given by the least-squares fit with the higher order constants constrained.

The wavenumbers for the other isotopic species of CO were calculated from constants given by Guelachvili et al. [5.301], but corrected to agree with the offset observed in the wavenumbers for the ^12^C^16^O species. The wavenumbers for the rarer isotopic species are given to help in correctly identifying the calibration lines.

#### 3.2.5 NO

The wavenumbers of the line positions used to produce the NO atlas were calculated using the constants given by Hinz et al. [5.371] for the ^14^N^16^O species. The constants given by Amiot and Guelachvili [5.355] were used for ^15^N^16^O and those given by Amiot et al. [5.350] were used for ^14^N^18^O Those were the only transitions strong enough to show on the spectral plots. The uncertainties given in the tables are only estimates based on the accuracy of other heterodyne measurements and an estimate of the accuracy of the Hamiltonian used to fit the data.

The tables only give the wavenumber values for lines of ^14^N^16^O. Because only one paper [5.371] reports infrared frequency measurements for NO, the NO lines are not recommended for calibration in those regions where either OCS or N_2_O transitions are available for calibration. For many spectrometers most of the NO transitions are unresolved doublets. At low pressures the doublets will have equal intensities and widths so that no appreciable errors will be incurred by using the average frequency of the doublet. A few of the low-*J* transitions, especially for the *Q*-branch lines, will have additional structure due to the interaction of the nuclear electric quadrupole moment of the nitrogen atom with the surrounding charge distribution. Such small splittings will be no larger than a few megahertz and the average line positions given in the tables will not be affected.

### 3.3 Intensity Calculations

This section is intended to show how the transition intensities were calculated and how the intensity was defined. This will enable users to determine the intensity to be expected for conditions other than those used to prepare the atlas. In order to estimate the appearance of the spectrum under conditions of pressure broadening and spectrometer resolution different from that used to produce the atlas figures, it is also necessary to consider the line shapes and the effect of finite slit functions. Such effects are discussed in Sec. 4.

The integrated intensity of an individual line representing a single rovibrational transition is independent of the line shape. For this atlas we take the integrated line intensity, *S*, to mean

$$S=\int_{-\infty }^{+\infty }k(v)dv=(1/pl)\int_{-\infty }^{+\infty }\ln{\left(I_{0}/I\right)}_{v}dv,$$ (3.9)

where *k*(*v*) is the absorption coefficient at frequency *v, p* is the partial pressure of the gas, *l* is the length of the absorption path, *I*_0_ is the intensity of radiation without absorption from the line in question, and *I* is the intensity of radiation after absorption by the line. Note that Eq. (3.9) is only concerned with the absorption due to a particular transition.

The integrated intensities, as given in this atlas, were calculated by using the equation

$$\begin{array}{l} S=\exp(-E^{\prime \prime }/kT)[1-\exp(-v/0.69504T)]\\ (N_{i}/Q_{V}Q_{R})\times vC{\left|R_{v^{\prime \prime }l^{\prime \prime }J^{\prime \prime }}^{v^{\prime }l^{\prime }J^{\prime }}\right|}^{2}S_{v}^{2}S_{R}^{2}, \end{array}$$ (3.10)

where *C* is a proportionality constant that includes the factors *8π*^3^/3*hc* and other factors, such as the Loschmidt constant (2.686 763 × 10^25^ molecules/m^3^), required to give *S* in appropriate units. If S is in units of cm/molecule at STP, *C* is 4.162 38 × 10^−19^ cm^2^ D^−2^/molecule. If *S* is in units of cm^−2^ atm^−1^ at temperature *T, C* would be 3054.7262/*T* cm^−1^ D^−2^ atm^−1^. In Eq. (3.10) $\left|R_{v^{\prime \prime }l^{\prime \prime }J^{\prime \prime }}^{v^{\prime }l^{\prime }J^{\prime }}\right|$ is the vibrational transition moment or dipole derivative in de-bye units (1D = 3.335 64 × 10^−30^ *C* m), *v* is the transition wavenumber in units of cm^−1^, *T* is the temperature in kelvin, *N*_i_ is the concentration of the isotopic species under consideration, *Q*_v_ and *Q*_R_ are the vibrational and rotational partition functions respectively, and *S*_v_ and *S*_R_ are vibrational and rotational strength factors that should be included in the intensity. Some workers prefer to include *S*_V_ in the transition moment but we prefer to express it separately so the transition moment can be seen to be nearly the same for the ground state transitions and the accompanying hot bands. *S*_R_ is also called the direction cosine matrix element. The intensities given in these tables were calculated for a temperature of 296 K.

In Eq. (3.10) the term *N*_i_/*Q*_v_*Q*_R_ compensates for the fact that the pressure used in Eq. (3.9) is the total pressure of the gas being measured, including all isotopic species. That is to say, the pressure does not take into account the isotopic concentration or the number of molecules in different states. The values of *N*_i_ were calculated from the isotopic abundances given in Table 10 and taken from Refs. [3.5, 3.6]. The vibrational partition function was calculated by summing the Boltzmann population of the vibrational energy levels. The vibrational partition functions are given in Table 11.

The rotational partition functions were calculated from the equations given by McDowell [3.7]. A different rotational partition function was calculated for each vibrational energy level and for each isotopomer.

Some workers like to use *S*^0^ =* S/N*_i_ for 5 because *S*^0^ seems to be a more appropriate molecular property. On the other hand, S is more useful for analytical purposes, such as the determination of the amount of CO in the atmosphere. Actually, it is |*R*| or |*R*|^2^, rather than *S*, that is the true molecular property in Eq. (3.10), and, as suggested by Toth [5.218], all intensity measurements should report the value of |*R*|, or |*R*|^2^. Unfortunately, some authors have left out the *N*_i_ term and report values of |*R*| that are not true molecular properties relatable to the electron distribution in the molecule.

In the infancy of infrared spectroscopy the instrumentation was unable to resolve individual rovibrational transitions, so many early papers measured the intensity of entire vibrational bands. Those papers measured band intensities by using a modification of Eq. (3.9) in which the integration was over the entire band rather than over a single line. The fine points of isotopic concentration and vibrational hot bands were ignored. Even today integrated band intensities are often measured for heavier molecules for which the density of lines is very high.

From those early measurements the term band intensity came to mean the intensity of all the lines in a band including all isotopes present in a normal sample and all hot bands. As a first approximation such a band intensity can be calculated from Eq. (3.10) if the terms $S_{V}^{2}S_{R}^{2}$, and *N*_i_/*Q*_V_*Q*_R_ are all omitted and *v* is set equal to the center of the band. This band intensity will generally be within a few percent of the intensity obtained by adding all the line intensities for the band. To be faithful to the original meaning, the true band intensity should be the sum of the intensities of all the lines in the band, including all isotopes and all hot bands.

#### 3.3.1 Calculation of the Strength Factors

The vibrational strength factors were slightly different for even and odd values of *Δv*_2_. For simplicity the strength factor was broken into two factors such that

$$S_{v}^{2}=S_{13}^{2}S_{2}^{2}$$ (3.11)

with

$$S_{13}^{2}=(v_{1}+\Delta v_{1})!(v_{3}+\Delta v_{3})!/(v_{1}!v_{3}!\Delta v_{1}!\Delta v_{3}!),$$ (3.12)

and for *Δv*_2_ even (or zero)

$$\begin{array}{l} S_{2}^{2}=[1/2(v_{2}+l+\Delta v_{2})]![1/2(v_{2}-l+\Delta v_{2})]!\\ /\{[1/2(v_{2}+l)]![1/2(v_{2}-l)]!\times {\left\{[1/2(\Delta v_{2})]!\right\}}^{2}\}, \end{array}$$ (3.13)

while for *Δv*_2_ odd

$$\begin{array}{c} S_{2}^{2}=[1/2(v_{2}+l+\Delta v_{2}-1)]![1/2(v_{2}-l+\Delta v_{2}-1)]!\\ (v_{2}+l\Delta l+\Delta v_{2}+1)/\{[1/2(v_{2}+l)]![1/2(v_{2}-l)]!\\ {\left\{[1/2(\Delta v_{2}-1)]!\right\}}^{2}(\Delta v_{2}+1)\}. \end{array}$$ (3.14)

In both cases *S*_2_ = 0 if |Δ*l*| > 1. In Eqs. (3.12)–(3.14) the smaller of *v*′ and *v*″ is used for *v*, *Δv = |v*′ *− v*″|, *l* = *l*″, and Δ*l* = *l*′ – *l*″. For the present calculations, *S*_2_ can be taken as the positive square-root of Eqs. (3.13) and (3.14).

Equation (3.12) was derived from the properties of harmonic oscillator wave functions such as can be found, among other places, in Appendix III of Ref. [3.8]. Equations (3.13) and (3.14) were derived from the properties of the two-dimensional harmonic oscillator wave functions given by Moffitt and Liehr [3.9].

Note that $S_{V}^{2}$ is normalized so that transitions from the ground state always have $S_{V}^{2}=1$. For certain hot bands, such as 2*v*_1_ − *v*_1_, $S_{V}^{2}=2$, while for the hot band 3*v*_1_ − *v*_1_, $S_{V}^{2}=3$. Other authors sometimes include *S*_V_ in the transition moment, |*R*|, in which case the transition moment for certain hot bands will be very different from the transition moment for the ground state transitions.

For transitions for which *v*_2_ = 0 (and *l* = 0) the rotational strength factors are given by

$$S_{R}^{2}=|m|\text{for}\;\Delta J=\pm 1$$

and

$$S_{R}^{2}=0\;\text{for}\;\Delta J=0,$$

where *m* has the usual meaning of −*J*″ for *ΔJ* = −1 and *J*″ + l for *ΔJ* = +1.

If *v*_2_ ≠ 0, the *l*-type resonance energy matrix was used and the intensity was obtained by multiplying each term of the eigenvector, for the appropriate eigenvalue, by the appropriate intensity factor given by *S*_V_*S*_R_ where *S*_R_ was determined from Table 2.1 of Gordy and Cook [3.11], or Table 4-4 of Townes and Schawlow [3.12]. Di Lauro and Mills [3.13] describe a similar procedure for determining intensities of transitions in Coriolis coupled levels of a symmetric rotor. For the specific cases of *l*-type resonance, this procedure was described by Maki et al. [3.14] although they dealt with the symmetry factored matrix whereas the present calculations used the unfactored matrix, such as Eq. (3.7).

To understand this intensity calculation let us consider a transition from an unperturbed lower state (*v*_1_, *v*_2_, *l*, *v*_3_, *J*) to an upper state that is involved in *l*-type resonance, (*v*_1_′, *v*_2_″, *l*′, *v*_3_′, *J*′) = (*v*_1_ + *Δv*_1_, *v*_2_ *+ Δv*_2_,*l + Δl, v*_3_ *+ Δv*_3_, *J*′). The upper state energy is given by a particular eigenvalue, *E*_1_. The eigenvector for this eigenvalue gives the mixing coefficients α_11_, α_12_, α_13_, etc. that measure the contribution of each unperturbed state to the perturbed state. The transition intensity is given by

$$\begin{array}{l} |<v_{1}+\Delta v_{1},v_{2}+\Delta v_{2},l+\Delta l,v_{3}+\Delta v_{3},J+\Delta J|\mu {\left|v_{1},v_{2},l,v_{3},J>\right|}^{2}=\\ |a_{11}<v_{1}+\Delta v_{1},v_{2}+\Delta v_{2},l^{\prime }=v_{2}+\Delta v_{2},v_{3}+\Delta v_{3},J\\ +\Delta J|\mu |v_{1},v_{2}l,v_{3},J>\\ +a_{12}<v_{1}+\Delta v_{1},v_{2}+\Delta v_{2},l^{\prime }\\ =v_{2}+\Delta v_{2}-2,v_{3}+\Delta v_{3},J+\Delta J|\mu |v_{1},v_{2},l,v_{3},J>\\ +a_{13}<v_{1}+\Delta v_{1},v_{2}+\Delta v_{2},l^{\prime }\\ =v_{2}+\Delta v_{2}-4,v_{3}+\Delta v_{3},J+\Delta J|\mu |v_{1},v_{2},l,v_{3},J>\\ {\ldots |}^{2}. \end{array}$$ (3.15)

All the upper state quantum numbers on the left side of Eq. (3.15) are the same as those on the right side except the *l*′ values. The *l*-type resonance mixes levels that differ only in the value of *l*.

If one assumes that the transition moment, or dipole derivative, is the same for all values of *l*, then Eq. (3.15) can be rewritten

$$\begin{array}{l} {\left|R\right|}^{2}S_{V}^{2}S_{R}^{2}={\left|R\right|}^{2}S_{13}^{2}\{a_{11}S_{2}S_{R}(v_{2}+\Delta v_{2},l\\ =v_{2}+\Delta v_{2},J^{\prime }\leftarrow v_{2},l^{\prime \prime },J^{\prime \prime })+a_{12}S_{2}S_{R}(v_{2}+\Delta v_{2},l\\ {=v_{2}+\Delta v_{2}-2,J^{\prime }\leftarrow v_{2},l^{\prime \prime },J^{\prime \prime })+a_{13}\ldots \}}^{2}. \end{array}$$ (3.16)

Each term within the curly brackets on the right side of Eq. (3.16) has a different value for *S*_2_ and *S*_R_ depending on the unperturbed transition to which they apply. In many cases they will be zero because they apply to |*Δl*| > 1. In Eqs. (3.12)–(3.14) we have already given the formulas for the values of $S_{13}^{2}$ and $S_{2}^{2}$.

The only nonzero values of *S*_R_ are given below. For *Δl* = 0, the *ΔJ* = 0 transitions have

$$S_{R}={\left[\left(2J+1)l^{2}/J(J+1\right)\right]}^{1/2}$$ (3.17)

and the *ΔJ* = ± 1 transitions have

$$S_{R}={\left[\left({\left|m\right|}^{2}-l^{2})/|m\right|\right]}^{1/2}.$$ (3.18)

For *Δl* = ± 1, the expressions for *ΔJ* = 0 were

$$S_{R}=1/2{\left[\left(2J+1)(J+l)(J-l+1)/J(J+1\right)\right]}^{1/2},$$ (3.19)

where *l* is the larger of *l*′ and *l*″. For *Δl* = + 1 and *ΔJ* = + l,

$$S_{R}=-1/2{\left[(J+l)(J+l+1)/J\right]}^{1/2};$$ (3.20)

for *Δl* = + 1 and *ΔJ* = − 1,

$$S_{R}=1/2{\left[(-J+l)(-J+l+1)/J\right]}^{1/2};$$ (3.21)

for *Δl* = − 1 and *ΔJ* = + 1,

$$S_{R}=1/2{\left[(J-l)(J-l+1)/J\right]}^{1/2};$$ (3.22)

and for *Δl = −* 1 and *ΔJ* = − 1,

$$S_{R}=-1/2{\left[(-J-l)(-J-l+1)/J\right]}^{1/2}.$$ (3.23)

In Eqs. (3.19)−(3.23) *J* is the larger of *J*′ and *J″* but *l* is always *l*″.

When both the upper and lower states are involved in *l*-type resonance, Eqs. (3.15) and (3.16) must be modified to include the eigenvectors for both the upper and lower states, otherwise the treatment is the same.

#### 3.3.2 Herman-Wallis Terms

Sometimes it is necessary to divide ${\left|R_{v^{\prime \prime }l^{\prime \prime }J^{\prime \prime }}^{v^{\prime }l^{\prime }J^{\prime }}\right|}^{2}$ into two terms such that

$${\left|R_{v^{\prime \prime }l^{\prime \prime }J^{\prime \prime }}^{v^{\prime }l^{\prime }J^{\prime }}\right|}^{2}={\left|R_{v^{\prime \prime }l^{\prime \prime }}^{v^{\prime }l^{\prime }}\right|}^{2}F_{J^{\prime \prime }}^{J^{\prime }},$$ (3.24)

where $F_{J^{\prime \prime }}^{J^{\prime }}$ is similar to the Herman-Wallis term [3.15, 3.16]. There are several forms that have been used for the $F_{J^{\prime \prime }}^{J^{\prime }}$ term. For the overtone of CO the form used was

$$F_{J^{\prime \prime }}^{J^{\prime }}=1+C_{1}m+C_{2}m^{2}.$$ (3.25)

Toth [5.218], in his extensive intensity measurements for N_2_O, has given the values for the Herman-Wallis constants for several bands. Toth has used several formulations such as Eq. (3.25) above and also

$$F_{J^{\prime \prime }}^{J^{\prime }}=[1+aJ(J+1)+bJ^{2}{\left(J+1\right)}^{2}]$$ (3.26)

$$F_{J^{\prime \prime }}^{J^{\prime }}={\left[1+a_{1}\;m+a_{2}J^{\prime }(J^{\prime }+1)\right]}^{2}$$ (3.27)

$$F_{J^{\prime \prime }}^{J^{\prime }}={\left[1+\xi_{1}m+\xi_{2}m^{2}\right]}^{2}.$$ (3.28)

In the case of OCS, Dang-Nhu and Guelachvili [5.104] used the form of Eq. (3.25) for the 11^1^0-00^0^0 band. The same constant was also used to calculate the intensities of the hot bands for which Δ*v*_1_ = 1 and Δ*v*_2_ = 1. The same form and, coincidentally, almost the same value for the Herman-Wallis term was used for the *v*_2_ band system. As recommended by Maki et al. [5.132], we used

$$F_{J^{\prime \prime }}^{J^{\prime }}=[1+a_{3}J\prime (J\prime +1)]$$ (3.29)

for the hot bands 10^0^2-00^0^1 and 04^0^2-00^0^1 in order to allow for the resonance interaction of the upper states. For the other transitions, there seemed to be no need for using a Herman-Wallis term in the intensity calculations.

For the 03^1^0-01^1^0 band of OCS, Depannemaecker and Lemaire [5.118] found that the first term, *C*_1_, in Eq. (3.25) was needed although no Herman-Wallis term was needed for the 02^0^0-00^0^0 band. On the other hand, the more extensive measurements of Blanquet et al. [5.134] showed that a Herman-Wallis term would improve the intensity fit for 02^0^0-00^0^0.

### 3.4 Data Sources for Intensity Calculations

#### 3.4.1 OCS

Only a small number of intensity measurements have been published for OCS. Most of them are measurements of integrated band intensities including transitions from the ground state as well as from other low energy states populated at room temperature, hot bands. The most thorough studies of band intensities are those of Foord and Whiffen [5.49] and Kagann [5.89] where references to earlier work may be found. Kagann’s values were consistently smaller by 11 to 15 percent except for the strongest band near 2062 cm^−1^ for which Kagann found an intensity that was 18 percent *larger.*

There have been only nine papers reporting intensity measurements of individual rovibrational transitions [5.73, 5.74, 5.83, 5.85, 5.103, 5.104, 5.118, 5.127, 5.134] and the first three of those papers are considerably less thorough than the others. In addition, there are two papers that give information on the relative intensities of well resolved rovibrational transitions without making absolute intensity measurements [5.101, 5.132].

Several papers [5.49, 5.97, 5.100, 5.106, 5.112] have used intensity and/or dipole moment measurements to derive a dipole moment function for OCS. At the present time the dipole function of OCS can be used to predict the dipole moment of OCS in different vibrational states, but it is not very useful for predicting transition intensities. The accuracy of the dipole moment function for predicting dipole moments is due largely to the fact that it is based on very accurate laser-Stark measurements involving many vibrational states.

Table 12 summarizes the intensity measurements reported in the literature. Table 12 also gives the transition moments used in the calculations for this atlas and the integrated intensity of each band as obtained by actually summing all of the transitions within each band, including all hot bands and all isotopic species. In a few cases, where indicated by a footnote, the integrated intensity given in the literature and reported in Table 12 does not include the hot band intensity. In those cases Table 12 gives the intensity calculated without including the contribution from hot bands in order to more easily compare the intensity used in this atlas with what was reported in the literature.

Only a few bands of OCS show any need for Herman-Wallis terms. As is usually the case, the two perpendicular bands, *v*_2_ and *v*_1_ + *v*_2_, required a small Herman-Wallis term, *C*_1_ = 0.0045 and 0.00463 for the two bands, respectively. The Herman-Wallis term is defined by Eqs. (3.24) and (3.25). For *v*^2^ it was estimated by us from inspection of the experimental spectrum and is not a very accurate value. The Herman-Wallis term for *v*_1_ *+ v*_2_ was measured by Dang-Nhu and Guelachvili [5.104]. Although the Herman-Wallis term is likely to be somewhat different for different isotopes and for hot bands, the same term was used for all bands involving the same quantum number changes unless indicated in the key that accompanies the tables.

The only other bands that were given nonzero Herman-Wallis constants were some of the hot bands that go with 2*v*_2_ and the hot bands 04^0^2-00^0^l and 10^0^2-00^0^1 which involve resonance coupled upper states. Depannemaecker and Lemaire [5.118] determined that *C*_1_ = 0.0019 gives the best fit to their intensity data for 03^1^0-01^1^0 transitions. We have used the same Herman-Wallis term for most of the other hot bands accompanying 2*v*_2_; see the descriptive key accompanying the 1000 to 1095 cm^−1^ tables. Maki et al. [5.132] have given effective constants *a*_3_ = 0.00105 and *a_3_*= −0.00034, respectively, to the resonance coupled transitions. The latter constants are based on Eqs. (3.24) and (3.29).

After the tables were prepared the new intensity measurements of Blanquet et al. [5.134] became available and it leads us to believe that the intensity values in the tables are too high by about 7 percent because we assumed that the dipole derivative given by Depannemaecker and Lemaire took into account the isotopic abundance. Any revised intensity calculations for the 2*v*_2_ band should also include the Herman-Wallis constant measured by Blanquet et al. [5.134].

#### 3.4.2 N_2_O

A great many intensity measurements have been made on the various bands of N_2_O given in this atlas. Not all the measurements are given in Table 13, but the most important or most recent measurements are given there.

As was the case for OCS, the earlier measurements were of the intensity of the unresolved bands. Such measurements included all hot bands and all isotopic species present in a normal sample. One of the more complete sets of measurements is that reported by Kagann [5.214]. Many earlier measurements are summarized in that paper. Kobayashi and Suzuki [5.228] have fit the available data on intensities to a dipole moment function from which they calculate transition moments and Herman-Wallis constants. More measurements are needed to evaluate the accuracy of the constants calculated from their dipole moment function.

Because of the interest in N_2_O as an atmospheric gas, the more recent intensity measurements involve spectra with resolved rotational structure and individual rovibrational lines have been measured. In many places in Table 13 we give both the intensity of the transitions from the ground state and the intensity of all transitions with the same change of quantum numbers regardless of the lower state. That allows one to compare our intensities with those reported in the literature.

For most of the transitions we have tried to use what we judge to be the best measurements for the intensity but in a few cases we have departed from the literature values in order to give a more realistic appearance to the spectrum. For example, we have used the transition moment given by Toth [5.218] for the *v*_3_ band near 1270 cm^−1^, but the transition moment given for the 2*v*_2_ band did not give a calculated spectrum that matched the observed spectrum where the two bands overlap. This mismatch caused us to use a slightly smaller value for the transition moment for 2*v*_2_ than that given by Toth. It is possible that either the Herman-Wallis constants or the intensity of the *v*_3_ band should have been changed. The more reasonable thing seemed to be to change the intensity of the weaker band. We have also recognized that Toth has included the vibrational factors in the dipole moment matrix elements that he reported, whereas Eq. (3.10) treats that as a separate term that multiplies the transition moment.

The two bands 2*v*_2_ + *v*_3_ and 2*v*_3_ also overlap and again there was some modification of the literature values for the intensities in order to get a good agreement between the calculated spectrum and that obtained experimentally.

We know of no intensity measurements for the *v*_1_ – 2*v*_2_ transitions between 990 and 1090 cm^−1^; consequently, we have estimated the intensity for that region. The true intensity could be quite different from what we have estimated, but the relative intensities of the lines in that region are probably good enough to recognize and assign the lines needed for calibration. That band is so weak that it is probably useless as a source of calibration for many workers, but it was included because it is based on frequency measurements. Since the lower energy level of the band is quite high, heating the absorption cell will make a large difference in the intensity.

Please see Eqs. (3.24) to (3.29) for the definition of the various Herman-Wallis constants used in this work. In his intensity studies [5.218, 5.185] Toth has given Herman-Wallis terms for all the bands. For the most part we have used those constants in the intensity calculations for the tables and figures. Several exceptions were made, however. For the 03^1^0-01^1^0 hot band we have used the same Herman-Wallis terms as given by Toth for the 02^0^0-00^0^0 band. For the other transitions involving Δv_2_ = 2, we have estimated the intensity constants to be used for the calculations because they were not included in Toth’s tables.

There seems to be no determination of a Herman-Wallis constant for the Δv_2_ = 1 transitions, therefore, we used *a*_1_ = 0.0016 which was estimated from the experimental spectrum. This value was estimated from laboratory spectra that were compared with the calculated spectrum. The intensities of the lines in the bands in the 1835–1925 cm^−1^ region were all calculated with ξ_1_ = −0.0107 as measured by Toth and Farmer [5.185]. The two Herman-Wallis constants measured by Boissy et al. [5.187] for the *v*_1_ band, were used for all the *Δ*v_1_ = 1 transitions including hot bands and different isotopomers used in the tables. The 2*v*_2_ *+ v*_3_ band also seemed to require a Herman-Wallis term although the spectra were better matched with a value of *ξ*_2_ = 0.3 × 10^−4^ rather than the term given by Levy et al. [5.216]. No Herman-Wallis terms were used in the intensity calculations for the *v*_1_ − *v*_3_, *v*_1_ *–* 2*v*_2_, 2*v*_3_, and *v*_1_ *+ v*_2_ bands and the hot bands that accompany them.

#### 3.4.3 CS_2_

Only a few intensity measurements have been made on the *v*_3_ band of CS_2_ [5.375, 5.377, 5.378, 5.379]. There seem to be no measurements of individually resolved rovibrational transitions, only integrated band intensities. For the purposes of this atlas we have calculated all hot band and isotopomer transitions with the same transition moment, 0.27 debye, and with no Herman-Wallis terms. The integrated intensity for the 1500 cm^−1^ band region of CS_2_ is 9.309 × 10^−17^ cm/molecule (2308 cm^−2^ atm^−1^ at 296 K) as determined from adding all the line intensities including hot bands and different isotopes. This may be compared to the value 9.55 × 10^−17^ cm/molecule given by McKean et al. [5.378], 9.38 × 10^−17^ cm/molecule given by Robinson [5.375], 9.23 × 10^−17^ cm/molecule given by Kiyama and Ozawa [5.377], and 9.13 × 10^−17^ cm/molecule given by Person and Hall [5.379].

#### 3.4.4 CO

##### Fundamental Band

Because of the large spacing between lines, CO was one of the first molecules for which individual line intensities were measured [5.255]. More recent measurements are tabulated in the review article by Smith et al. [5.2]. For the fundamental band near 2143 cm^−1^ it is difficult to determine which measurements are best but most of the recent measurements give integrated band intensities close to 1.027 × 10^−17^ cm/molecule (276 cm^−2^ atm^−1^ at 273.15 K). For this atlas we have used a dipole transition moment of 0.1073 D which gives a total band intensity of 1.03 × 10^−17^ cm/molecule (276 cm^−2^ atm^−1^ at 273.15 K or 255 cm^−2^ atm^−1^ at 296 K). The changes in the matrix elements for the less abundant isotopic species were taken from the theoretical calculations of Chackerian and Tipping [5.305]. The isotopic abundance was taken from Table 11. The uncertainty in the total band intensity seems to be about 3 percent. The intensity measurements of Chackerian et al. [5.300] (1.027 × 10^−17^ cm/molecule) are in agreement with other recent measurements for the main isotopic species and they also have studied the intensity of the weaker isotopic transitions. They gave transition intensities calculated from the electric dipole moment function given by Chackerian and Tipping [5.305]. Their calculated intensities seem to include a weak Herman-Wallis effect but they do not give any explicit constants for easily calculating that effect. A calculation that duplicates their temperature and dipole derivative agrees with their values to within one percent (for *J* < 35) even without including a Herman-Wallis effect.

In an earlier paper [5.282] Tipping gave calculated values for the Herman-Wallis constants but they were small enough to ignore for this atlas. Bouanich [5.310] has also given calculated values for the Herman-Wallis constants. Apparently there are only two experimental determinations of Herman-Wallis constants that have been reported for the fundamental band of CO [5.261, 5.318]. The earlier work is subject to question because of the sensitivity to temperature errors. The most recent measurement was reported after this work was done and would change the intensities by no more than one percent. The intensity calculations used for the atlas did not include a Herman-Wallis effect for the fundamental band.

##### First Overtone

Fewer intensity measurements have been made on individual lines of the overtone band near 4260 cm^−1^. A good average of the more recent values seems to give an integrated band intensity of 7.78 × 10^−20^ cm/molecule (2.09 cm^−2^ atm^−1^ at 273.15 K) with an uncertainty of about 6 percent. This is equivalent to a transition dipole matrix element of 0.0066 D.

The Herman-Wallis effect is significant for the first overtone band and has been included in the intensity calculation for the atlas. The constants given by Tipping [5.282], *C*_1_ = 0.005 and *C*_2_ = 0.000 034 [see Eq. (3.25)], were used for the calculation of the intensities of the first overtone transitions. Those calculated Herman-Wallis constants were in agreement with three experimental determinations [5.265, 5.271, 5.295] as well as with the calculation of Toth et al. [5.265].

#### 3.4.5 NO

A transition moment of 0.00412 D was used to calculate the line intensities given in the tables. This gives an integrated band intensity of about 5.04 × 10^−18^ cm/molecule (125 cm^−2^ atm^−1^ at 296 K) which agrees with the measurements of King and Crawford [5.330], Mandin et al. [5.359], and Holland et al. [5.368]. Some earlier measurements [5.326, 5.344, 5.347, 5.364, 5.367] and even some recent measurements [5.374] indicate that the intensity might be more like 4.44 to 4.64 × 10^−18^ cm/molecule (110 to 115 cm^−2^ atm^−1^ at 296 K) so the intensities given in the tables may be too large by about 10 percent. We have taken the higher intensity value because NO is prone to having impurities that are hard to remove, thus giving low intensity readings.

### 3.5 Other Line Parameters

#### 3.5.1 Lineshape and Pressure Broadening

Smith et al. [5.2] have given a good discussion of lineshapes and the determination of pressure broadening coefficients. We shall give here only a brief description to ensure that the reader understands the principal equations describing these effects. For a more complete understanding of the subject one should refer to the work of Smith et al. [5.2] and the papers to which they refer.

For a static gas in a field-free environment at pressures below one atmosphere, there are four major factors that might contribute to the shapes of infrared absorption lines, 1) lifetime broadening, 2) Doppler broadening, 3) pressure broadening, and 4) collisional narrowing.

##### Lifetime Broadening

The lifetime broadening of a state *n* is given by

$$\gamma_{n}=h/2\pi \tau_{n},$$

where *γ_n_* is the half-width of the state and τ*_n_* is the lifetime of that state. For a transition *i → j* between two states, *i* and *J*, the transition linewidth will be given by the lifetime of both upper and lower states,

$$\gamma_{i\to j}=(1/\tau_{i}+1/\tau_{j})h/2\pi .$$

For most stable molecules in the ground electronic state the radiative lifetime in a given rovibrational state will be on the order of 1 ms or more which gives a linewidth of about 1 × 10^−8^ cm^−1^ (0.16 kHz) or less. This is much smaller than the Doppler width even at temperatures on the order of 5 K. For Doppler-free measurements the effect of lifetime broadening could be important even for stable molecules although various instrumental effects, such as beam width or beam collimation, usually limit the effective linewidth. Transit time broadening is a variation of lifetime broadening where the lifetime is the time that the molecule is in the light-beam.

##### Doppler Broadening

The Doppler width is the result of the random motion of the molecules in a gas sample and is given by

$$\gamma_{D}=3.581\times 10^{-7}v{\left(T/M\right)}^{1/2},$$ (3.30)

where *v* is the wavenumber or frequency of the transition, *T* is the temperature of the gas in kelvin, *M* is the relative molecular mass of the molecule in atomic mass units, and *γ*_D_ is half the width of the transition at half the intensity (half-width at half height or HWHH). Since spectrometer resolution is often expressed in terms of the full width at half the line height (FWHH), the Doppler width is sometimes given by 2*γ*_D_.

For most gases at ambient temperatures and pressures below 2 kPa (15 Torr), the true lineshape (that is, the lineshape that would be observed with an instrument with infinite resolution) is dominated by the Doppler effect. For some molecules the effect of collisional narrowing, see below, is also important in that pressure regime. The Doppler effect gives a Gaussian lineshape which is described by the function

$$\begin{array}{l} f(v)=\{{\left[(\ln2)/\pi \right]}^{1/2}/\gamma_{D}\}\\ \exp\{-(\ln2){\left[(v-v_{0})/\gamma_{D}\right]}^{2}\} \end{array}$$ (3.31)

where *v*_0_ is the frequency (or wavenumber) of the center of the line.

In Eq. (3.31) the Gaussian shape function *f*(*v*) has been normalized so that

$$\int_{-\infty }^{+\infty }f(v)dv=1.$$ (3.32)

Consequently, since

k(v)=Sf(v), (3.33)

integrating both sides of Eq. (3.33) over all frequency space gives

$$\int_{-\infty }^{+\infty }k(v)dv=\int_{-\infty }^{+\infty }Sf(v)dv=S.$$ (3.34)

Here *k*(*v*) is the absorption coefficient at frequency *v* and *S* is the integrated line intensity given earlier in Eq. (3.9).

One important characteristic of the Gaussian lineshape is the small wing absorption due to the exponential reduction in absorption as one gets farther from the center of the line. As pointed out by Korb et al. [5.263], accurate intensity measurements are more easily made when the wing absorption is small. Another characteristic of the Gaussian shape is the bluntness at the center of the line.

##### Pressure Broadening

Pressure broadening gives rise to lines with a Lorentzian lineshape for which the normalized shape function has the form

$$f(v)=(\gamma_{L}/\pi )/[{\left(v-v_{0}\right)}^{2}+\gamma_{L}^{2}],$$ (3.35)

where *γ*_L_ is half the width of the line at the half intensity point. When pressure broadening is the dominant effect determining the lineshape, then Eq. (3.35) must be used in Eqs. (3.33) and (3.34).

Pressure broadening is an additive effect, therefore, the broadening of each gas in a mixture depends only on the partial pressure, *P_x_*, of that gas. The total broadening in a mixture is the sum of the broadening of each gas. Thus, for gases a and b with broadening coefficients *c*_a_ and *C*_b_, the total pressure broadened width will be *γ*_L_ = *c*_a_*P*_a_ + *c*_b_*P*_b_. The broadening coefficient is unique to each absorbing molecule and to each collision partner. The broadening coefficient is a function of the temperature as one might expect since the average collision velocity changes with temperature. The broadening coefficient is also different for each rotational transition although the changes are systematic with the rotational quantum numbers.

The pressure broadening coefficients generally have values in the range of 400 to 1300 MHz/Pa (3 to 10 MHz/Torr) and are greatest for molecules that have large dipole moments. The pressure broadening coefficients are generally smallest for those rotational energy levels that are at relatively high energy.

In contrast to Gaussian shaped lines, the Lorentzian shaped lines are sharper at the line center but have very extensive wings. For very strong lines it is possible to have significant absorption intensity twenty half-widths from the line center. This is noticeable in the CO atlas where there is only a small amount of pressure broadening and yet the strong lines have very noticeable wings. If there were no pressure broadening, the wings of the strong lines would not be so prominent.

##### Voigt Profile

In many cases the lineshape is determined both by the Doppler effect and by the effects of pressure broadening. In such cases the lineshape is more accurately given by a Voigt profile which is a convolution of the Gaussian and Lorentzian profiles. There is no good single closed-form expression for the Voigt profile. Rather, the Voigt shape function is given by the integral expression

$$f(v)=(Bx/\pi )\int_{-\infty }^{+\infty }[\exp(-y^{2})]/[x^{2}+{\left(z-y\right)}^{2}]dy$$ (3.36)

and various approximations to that integral. Equation (3.36) has been simplified by using

$$\begin{array}{l} B=(1/\gamma_{D}){\left[(\ln2)/\pi \right]}^{1/2},\\ x=(\gamma_{L}/\gamma_{D}){\left(\ln2\right)}^{1/2}, \end{array}$$

and

$$z=[(v-v_{0})/\gamma_{D}]{\left(\ln2\right)}^{1/2}.$$

There are a number of good computer programs [3.17, 3.18] for evaluating the Voigt shape function.

##### Collisional Narrowing

Collisional narrowing or Dicke narrowing [3.19–3.21] has only recently been measured for a few molecules, but its effects have been observed for NO [5.370]. Collisional narrowing has the effect of reducing the size of the Gaussian linewidth *γ*_D_. In other words, it makes the line appear to have a Doppler width that is smaller than that calculated by Eq. (3.30). For NO, the effective Gaussian width is about 9 percent smaller than the Doppler width at a pressure of 6.6 kPa (50 Torr). Since collisional narrowing is primarily a kinetic collisional effect (sometimes described in terms of hard and soft collision models), it is not expected to depend on the rotational or vibrational levels involved in the transition. There is, however, a weak dependence on the transition assignment as shown by Pine and Looney’s work on HCl and HF [3.22].

#### 3.5.2 Estimating Peak Intensities

From tables of line intensities, such as are given in this atlas, it is possible to estimate the pressure-pathlength product needed to obtain a spectrum with adequate intensity. For this purpose we consider the two limiting cases of Doppler (or Gaussian) shaped lines at low pressures and the pressure broadened Lorentzian lines for high pressures.

If the effect of pressure broadening is negligible and the lineshape is determined by the Doppler width rather than the spectrometer slit function, the percent transmission at the center of a line is given by

%transmission=100exp(−CSlp/γ), (3.37)

where *C* is 1.1494 × 10^14^ if *S* is the intensity given in the tables (in units of cm/molecule), *γ* is the Doppler width [given by Eq. (3.30)], *l* is the pathlength in centimeters, and *p* is the pressure in pascals (Pa). (If *p* is measured in Torr, then *C* = 1.5324 × 10^16^.) Note that C has been evaluated for a temperature of 296 K, which is the temperature for which the intensities have been calculated in this atlas.

If the lineshape is dominated by pressure broadening, the percent transmission is again given by Eq. (3.37), but *γ* should be the pressure-broadened linewidth, *γ*_L_, and *C* will be 7.789 × 10^13^ if *p* is in Pa (1.038 × 10^16^ if *p* is in Torr).

For the same linewidth and integrated intensity a Doppler-broadened line will have a peak intensity 1.476 times greater than a pressure-broadened line, even though the pressure broadened line is sharper.

For intermediate pressures the peak intensity can be more accurately estimated by modifying Eq. (3.37) so as to add the approximate contribution of both shapes according to

$$\%\;\text{transmission}=100\;\exp\{-Slp[(1.1494\times 10^{14}\gamma_{D}+7.789\times 10^{13}\gamma_{L})/(\gamma_{D}^{2}+\gamma_{L}^{2})]\}$$ (3.38)

Equation (3.38) reduces to Eq. (3.37) when either *γ*_D_ or *γ*_L_ dominates the lineshape.

The peak intensity observed with an instrument that introduces any instrumental broadening will, of course, be smaller than that calculated with either Eq. (3.37) or (3.38). As a rule of thumb, the peak intensity will be diminished by more than the ratio *γ*/*γ_S_*, where *γ* is the true linewidth and *γ_S_* is the width of the instrumental resolution function. Thus, for an instrument with a resolution function that is ten times greater than the true linewidth, an absorption line will appear at least ten times weaker than what is calculated by either Eq. (3.37) or Eq. (3.38), provided the line is not saturated.

#### 3.5.3 Pressure Induced Lineshifts

A good frequency calibration standard is one whose frequency is not changed as the measurement conditions are varied. Some of the absorption lines given in this atlas are weak enough to require that either long pathlengths or moderate pressures, 0.3 to 1.3 Pa (2 to 10 Torr), be used. It is important that one recognize the additional calibration uncertainty introduced by using pressures that are too high. Although pressure induced shifts in the frequency of the absorption lines are poorly understood and measurements are few and not very accurate, it is possible to estimate the approximate effect of moderate pressures on the frequencies of these calibration standards.

As a general rule, pressure induced frequency shifts are at least an order of magnitude smaller than pressure induced broadening. For the overtone of CO, Pollock et al. [5.304] found a pressure shift (due to CO) on the order of −2±1.5 kHz/Pa (−0.3 ± 0.2 MHz/Torr). Bouanich [5.302] found pressure shifts on the order of −1.2 ± 0.3 kHz/Pa (−0.16 ± 0.04 MHz/Torr). He seems to have observed a significant rotational dependence, but we only quote an average value. For the overtone of NO, Pine et al. [5.370] found a self-induced pressure shift of −1.1 ± 0.3 kHz/Pa (−0.15 ± 0.04 MHz/Torr). They found that there may be a weak rotational dependence but it was obscured by experimental error.

For N_2_O an attempt to measure the self-induced pressure shift in the rotational spectrum resulted in an estimate of an upper limit of 0.75 kHz/Pa [5.201]. The pressure shift of several lines of N_2_O were measured near 4500 cm^−1^ and an average value of −1.2 ± 0.3 kHz/Pa (−0.16 ± 0.04 MHz/Torr) was found [5.219]. The pressure shift caused by N_2_ and O_2_ on the *v*_3_ band of N_2_O near 1280 cm^−1^ was measured by Varanasi and Chudamani [5.238]. Their diode laser measurements gave an average value of about −0.6 ± 0.15 kHz/Pa (−0.08 MHz/Torr) for several lines between 1250 and 1300 cm^−1^.

More pressure shift measurements seem to have been made on OCS than on any of the other molecules in this atlas. The earlier measurements indicated that the pressure shift was on the order of 4.0 kHz/Pa (0.5 MHz/Torr) or less [5.94]. Later this estimate was improved by new measurements that gave an average value of −0.4 ± 1.5 kHz/Pa (−0.05 ± 0.20 MHz/Torr) [5.120]. The most recent measurements at about 1000 cm^−1^ [5.133] indicate that the self-shift is −0.37 ± 0.04 kHz/Pa (−0.049 + 0.005 MHz/Torr). Recent measurements of the self-shift of microwave transitions [5.119] found that the shift was too small to measure, 0.000 ± 0.04 kHz/Pa or 0.000 ±0.006 MHz/Torr.

Kou and Guelachvili [3.23] have recently measured the self-induced pressure shift for the CO_2_ laser lines near 1000 cm^−1^. They found the shift to be on the order of −1.05 ± 0.2 kHz/Pa (−0.14 ± 0.03 MHz/Torr) with no evidence of a *J*-dependence. One might expect that the self-induced pressure-shift of CO_2_ would be similar to that of the molecules used in the present compilation.

Since the pressure shift is a shift in the energy levels, it is likely to be greater as one goes to higher energy levels. As a first approximation one can probably assume that the shift is proportional to the frequency. It is also possible that for polyatomic molecules, the pressure shift may depend, to a large extent, on the vibrational mode involved.

For purposes of estimating the maximum error that could be introduced in a calibration measurement using any of the data given in this atlas, one should treat the frequencies given in this work as applying for a pressure below 130 Pa (1 Torr). For each increase in pressure of 130 Pa (1 Torr) the uncertainty in the frequency for N_2_O, NO, CO, and CS_2_ should be increased by 0.3 MHz unless the pressure shift is added to the frequencies given in these tables or unless more accurate values for the pressure shift become available. For OCS the uncertainty should be increased by about 0.1 MHz/Torr. Note that the pressure shift seems to be negative for all the molecules in this atlas.

### 3.6 References

[3.1] J. L. Dunham, The energy levels of a rotating vibrator, Phys. Rev. **41**, 721–731 (1932).
[3.2] A. G. Maki and D. R. Lide, Microwave and infrared measurements on HCN and DCN: observations on *l*-type resonance doublets, J. Chem. Phys. **47**, 3206–3210 (1967).
[3.3] J. M. Brown, J. T. Hougen, K.-P. Huber, J. W. C. Johns, I. Kopp. H. Lefebvre-Brion, A. M. Merer, D. A. Ramsay, J. Rostas, and R. N. Zare, The labeling of parity doublet levels in linear molecules, J. Mol. Spectrosc. **55**, 500–503 (1975).
[3.4] W. F. Rowe and E. B. Wilson, An Application of the Hellmann-Feynman Theorem to vibration-rotation interactions, J. Mol. Spectrosc. **56**, 163–165 (1975).
[3.5] H. S. Peiser, N. E. Holden, P. De Bievre, I. L. Barnes, R. Hagemann, J. R. DeLaeter, T. J. Murphy, E. Roth, M. Shima, and H. G. Thode, Element by element review of their atomic weights, Pure Appl. Chem. **56**, 696–768 (1984).
[3.6] P. De Bievre, M. Gallet, N. E. Holden, and I. L. Barnes, Isotopic abundances and atomic weights of the elements, J. Phys. Chem. Ref. Data **13**, 809–891 (1984).
[3.7] R. S. McDowell, Rotational partition functions for linear molecules, J. Chem. Phys. **88**, 356–361 (1988).
[3.8] E. B. Wilson, J. C. Decius, and P. C. Cross, Molecular Vibrations—The Theory of Infrared and Raman Vibrational Spectra, McGraw-Hill Book Co, Inc., New York, 1955.
[3.9] W. Moffitt and A. D. Liehr, Configurational instability of degenerate electronic states, Phys. Rev. **106**, 1195–1200 (1957).
[3.10] R. H. Tipping, Vibration-rotation intensities for Hot bands, J. Mol. Spectrosc. **61**, 272–281 (1976).
[3.11] W. Gordy and R. L. Cook, Microwave Molecular Spectra, John Wiley and Sons, New York, 1970.
[3.12] C. H. Townes and A. L. Schawlow, Microwave Spectroscopy, McGraw-Hill Book Co., New York, 1955.
[3.13] C. Di Lauro and I. M. Mills, Coriolis interactions about X-Y axes in symmetric tops, J. Mol. Spectrosc. **21**, 386–413 (1966).
[3.14] A. G. Maki, W. B. Olson, and R. L. Sams, HCN rotational-vibrational energy levels and intensity anomalies determined from infrared measurements, J. Mol. Spectrosc. **36**, 433–447 (1970).
[3.15] R. Herman and R. F. Wallis, Influence of vibration-rotation interaction on line intensities in vibration-rotation bands of diatomic molecules, J. Chem. Phys. **23**, 637–646 (1955).
[3.16] R. H. Tipping and R. M. Herman, Line intensities in HBr vibration-rotation spectra, J. Mol. Spectrosc. **36**, 404–413 (1970).
[3.17] C. Young, Calibration of the absorption coefficient for lines with combined Doppler and Lorentz broadening, J. Quant. Spectrosc. Radiat. Transfer **5**, 549–552 (1965).
[3.18] S. R. Drayson, Rapid computation of the Voigt profile, J. Quant. Spectrosc. Radiat. Transfer **16**, 611–614 (1976).
[3.19] R. H. Dicke, The effect of collisions upon the Doppler width of spectral lines, Phys. Rev. **89**, 472–473 (1953); J. P. Wittke and R. H. Dicke, Redetermination of the hyperfine splitting in the ground state of atomic hydrogen, Phys. Rev. **103**, 620–631 (1956).
[3.20] L. Galatry, Simultaneous effect of Doppler and foreign gas broadening on spectral lines, Phys. Rev. **122**, 1218–1223 (1961).
[3.21] S. G. Rautian and I. I. Sobclman, The effect of collisions on the Doppler broadening of spectral lines, Sov. Phys. Usp. **9**, 701–716 (1967).
[3.22] A. S. Pine and J. P. Looncy, N_2_ and air broadening in the fundamental bands of HF and HCl, J. Mol. Spectrosc. **122**, 41–55 (1987).
[3.23] Q. Kou and G. Guelachvili, Self-induced pressure shifts in the 9.4- and 10.4-μm bands of CO_2_ by Fourier transform spectroscopy, J. Mol. Spectrosc. **148**, 324–328 (1991).

## 4. Error Analysis

The standards presented in this book are based on data derived in large part from frequency measurements because such measurements are less prone to systematic errors. In assessing the uncertainties that should be assigned to the recommended calibration frequencies, we must consider five major factors that may contribute to error in the determination of the line frequencies.

1. The accuracy of the calibration source.
2. The uncertainty in locating the center of the absorption line.
3. The accuracy of transferring the calibration to the line center measurement.
4. Errors caused by environmental effects.
5. Model errors, or uncertainties in the application of least-squares techniques to obtain the best estimate of the correct line frequency.

In succeeding paragraphs of this section each of these factors will be examined in relation to the calibration frequencies recommended in this book.

### 4.1 Accuracy of the Calibration Source

Much of the primary infrared data used for these tables originated at the NIST laboratory in Boulder, Colorado. Two other laboratories have reported heterodyne frequency measurements on the 2*v*_2_ band of OCS, [5.88, 5.126]. The most important of these were two saturated absorption measurements made by Fayt et al. [5.126] which considerably reduce the uncertainty in the frequencies for 2*v*_2_. The measurement of the laser transitions of N_2_O by Whitford et al. [5.183] gave accurate frequencies for the separation of the *v*_1_ and *v*_3_ states which were useful for the tables between 880 and 980 cm^−1^. Recently frequency measurements have been made by Urban and coworkers [5.321, 5.137a].

The NIST measurements used well characterized CO_2_ lasers that were virtually identical to the lasers used by Petersen et al. [2.9, 2.11, 2.12] in the last major determination of the CO_2_ laser frequencies. Since much of the laser technology that went into the determination of the CO_2_ laser frequencies was developed in the same laboratory where the heterodyne measurements were made, one can be sure of the accuracy of the calibration source. These lasers have been described in Sec. 2. The CO_2_ laser frequencies are good to at least ±0.05 MHz and are not a significant source of error for the Doppler limited heterodyne measurements.

### 4.2 Uncertainty in Locating the Line Center

Two major factors contribute to errors in the location or determination of absorption line centers: the signal-to-noise ratio, and the slope or other irregularities in the background.

For the infrared measurements the slope or irregular background in the radiation being absorbed was only a minor contributor to error since its effect could be practically eliminated, as described in Sec. 2.

In some cases imperfectly resolved or overlapping lines were measured. In cases where the lines are close doublets, the measurement represents the center of gravity of the doublets, and the least-squares analysis took that into account. If the lines were partially resolved, that was taken into account in assigning the uncertainty for the measurement. In most cases partially resolved lines that have unequal intensities should not be used unless a very generous allowance is made for the uncertainty since the resulting error is not random, but is in a particular direction. OCS, CO, and CS2 are good molecules to use for standards because they have no quadrupole fine structure. N_2_O is not as good because all of its transitions have a small quadrupole splitting. Since the splitting for N_2_O is never greater than 4 MHz and diminishes rapidly with increasing *J*-values and since the Doppler fullwidth of N_2_O at 500 cm^−1^ is 28 MHz, the splitting should not affect the use of N_2_O for most infrared calibration purposes.

For each infrared heterodyne frequency measurement two line centers must be measured: the absorption line center, and the center of the difference frequency (between the TDL and the local oscillator). The location of the absorption line center is determined by the technique used to lock the TDL to the absorption line as described in Sec. 2. The uncertainty assigned to the line-center lock is given by half the Doppler-width of the line divided by the signal-to-noise ratio of the derivative signal for the line. Because of modulation broadening the linewidth is slightly greater than the Doppler-width but this approximation to the lock uncertainty is adequate. The lock error is random and is reflected in the statistical analysis of the least-squares fit of many measurements.

More important than the uncertainty in locking to the line center is the uncertainty in the difference frequency measurements. That uncertainty is given by one-tenth the beat note linewidth, or by half the beat note width divided by the signal-to-noise ratio, whichever is larger. We believe this covers both random and residual systematic errors for a single measurement. In most of our measurements, this has been the predominant uncertainty and the lock uncertainty has been negligible by comparison.

One source of error in the heterodyne measurements that has no direct counterpart in wavelength measurements is due to frequency-dependent differences in the transmission or amplification components involved in the heterodyne frequency measurements. This frequency dependence sometimes presented an additional distortion to the heterodyne frequency lineshape as displayed by the spectrum analyzer. Fortunately this error, which would be systematic for all measurements of the same heterodyne frequency, becomes randomized if enough different lines are measured. In most cases this problem was recognized and was taken into account in estimating the uncertainty of each measurement.

Examination of our results over the past decade indicates that the procedure outlined above for assigning the uncertainty produced values which turned out to be close to a 2 σ uncertainty.

For the microwave and sub-millimeter wave measurements used to prepare these tables, the uncertainties given in the literature are generally accurate enough although we have increased the uncertainties slightly in a few cases. There may be some systematic error due to incomplete modulation when Stark modulation was used, but this is compensated by the much smaller linewidths, and the smaller frequency dependent irregularities, compared to the infrared measurements.

### 4.3 Accuracy of Transferring the Calibration to the Line Center Measurement

The primary difference in the reliability of wavelength and frequency measurements is in the accuracy of transferring the calibration to the measurement. In frequency measurements the accuracy of the measurements is not affected by beam dimensions, by wavefront mismatch or other misalignments, or by refractive index effects. The frequency of the radiation is always the same no matter what the medium may be, or how it is measured. Modern electronics excel at counting, and that is how frequency is determined. With frequency measurements, if a measurement can be made at all, the uncertainty must come from the four other sources of error discussed in this chapter.

On the other hand, wavelength comparisons are susceptible to many different wavelength-dependent errors, errors due to misalignment, errors due to differences in the ratio of the dimensions of the optical elements (or of the optical beam), to the wavelength. The best wavelength measurements use a calibration that is nearly the same wavelength and intensity as the feature to be calibrated. Each type of wavelength measurement has its own peculiarities.

The present tables are based in part on higher order centrifugal distortion constants determined to some extent by FTS measurements which are essentially wavelength measurements. These measurements were all internally calibrated by means of lines whose frequency could be determined by heterodyne frequency measurements. These calibration features were always within the approximately 120 cm^−1^ band-pass of the FTS measurements. For the FTS measurements there may be a phase error which is different for weak features and for strong features and that is one reason why greater emphasis was placed on the use of frequency measurements of pure rotational transitions to determine the rotational constants wherever possible. In no case were FTS measurements used to determine the band centers or vibrational levels for the lines recommended as frequency standards.

### 4.4 Errors Caused by Environmental Effects

For closed shell molecules the only significant environmental effects are due to pressure shifts and electric field effects such as either the ac or dc Stark effects. As long as the radiation field (for absorption spectra) is too low to give saturated absorption effects, the ac Stark effect can be ignored in its effect on the absorption line center. Even the dc Stark effect can be ignored for most work since stray electric fields are generally too small to give noticeable Stark shifts.

We believe that even in the case of the saturated absorption measurements on the 2-0 band of CO, the shift due to the ac Stark effect will be smaller than a tenth of the linewidth, or less than 0.2 MHz.

The ac Stark effect may have a small effect on the frequency of the CO_2_ lasers but, since those lasers have the same characteristics as the lasers used in the original measurements against the cesium frequency standard, the effect of the shift will already be included in the frequency assigned to the laser lines. In the case of the CO transfer oscillators, the CO frequencies are measured at the same time as the beat note frequency measurement and any ac shift is included in the measurement.

On the other hand, the effect of pressure induced shifts is potentially significant and deserves serious consideration. Pressure-induced shifts in infrared spectra have not been extensively studied, so there is no experimentally confirmed theory that one can use to calculate the pressure shifts to be expected for much of the data given in the present tables. All of the pressure shift measurements seem to indicate that the shift for the transitions given in these tables may be on the order of −2.2kHz/Pa (−0.3 MHz/Torr) or less. Only in the case of the measurements given by Vanek et al. [9.133] is the shift of −0.37 ±0.04 kHz/Pa (−49 ±5 kHz/Torr) reliably given for the 2*v*_2_ band of OCS. Since neither the frequency dependence nor the rotational or vibrational dependence of the pressure shift is known, this remains one of the most important uncertainties in the application of these tables to real measurements.

### 4.5 Uncertainties in the Application of Least-Squares Techniques

Because individual measurements of infrared absorption lines by heterodyne measurement techniques are not very precise (uncertainties of the order of 5 to 10 MHz for some regions) and only a small number of transitions can be measured, it is necessary to use least-squares techniques to combine all available measurements to yield calculated transitions that are considerably more accurate than any one measurement. Such fitting techniques can be no more accurate than the equations used to describe the transitions (the Hamiltonian). The molecules and the particular transitions in these tables were chosen in part because of the reliability of the Hamiltonian as shown by extensive studies reported in the literature. Although certain interactions, such as Fermi resonance, affect the constants used in the effective Hamiltonian, such resonances can be ignored, provided that measurements are available for a wide range of rotational levels for each vibrational state. In the analysis used in this book the effect of *l*-type resonance was included because it represents the largest resonance effect and it can be reliably estimated, thereby giving the correct functional form to the centrifugal distortion constants. The details of the fitting procedure and Hamiltonian are given in Sec. 3.

The variance-covariance matrix determined by the least-squares fit gives a reliable estimate of the uncertainties of the transitions in the range of rotational levels for which there are good measurements. The calculated uncertainties get large quite rapidly for transitions extrapolated beyond the range of measured energy levels, but the reliability of those calculated uncertainties deteriorates even more quickly. For that reason we have terminated the recommended calibration standard indication at the highest *J*-value for which there are good measurements. Higher transitions are given in the tables but their accuracy is less certain.

### 4.6 Summary

Of the five sources of error identified above, the first (the accuracy of the calibration source) does not contribute significantly to the uncertainties in the calibration frequencies given in these tables. For frequency measurements the second and third source (uncertainty in locating the line center and accuracy of transferring the calibration) will appear as random errors and so will be given by the statistical analysis of the least-squares fit of the measurements. For the molecules and bands represented in these tables the uncertainties contributed by model errors are small and likely to show as deviations that are included in the statistical analysis.

In conclusion we think that the uncertainties given by the statistical analysis are adequate to describe the errors in the frequency measurements that might arise from all causes except errors due to pressure-induced frequency shifts. Most of the heterodyne measurements were made at low pressures but some measurements of weaker transitions were made at pressures as great as 1200 Pa (9 Torr). To allow an extra margin of error due to pressure shifts, the uncertainties assigned to the heterodyne frequency measurement data used in the fit were about twice as large as the rms deviation. Primarily, this had the effect of increasing the uncertainty in the vibrational energy levels.

## 5. Bibliography

We attempt here to list all references to papers giving infrared or microwave frequency (or wave-number) measurements, as well as lineshape and intensity measurements that are relevant to this atlas. Papers involving bands not included in this atlas may be missing from this bibliography. Some papers are not included if they involve foreign gas broadening measurements only. For a more complete listing of pressure broadening papers see the review given by Smith et al. [5.2]. When completely superseded, some of the older papers may not appear in this bibliography but they can be found referenced in the more recent papers. The references are grouped by molecule and arranged in chronological order for each molecule. At the end of each reference is a list of initials that indicate the subject matter covered by the reference as follows:

F — frequency measurements,
FB — foreign gas broadening measurements,
I — intensity measurements,
LS — laser-Stark measurements,
PS — pressure shift measurements,
SB — self-broadening measurements,
T — theory, and
W —wavenumber or wavelength measurements.

### 5.1 Intensity and Pressure Broadening Bibliographies

[5.1] L. A. Pugh and K. Narahari Rao, in Molecular Spectroscopy: Modern Research, Vol. II (K. Narahari Rao, ed.), pp. 165–227 Academic Press, New York (1976) I.
[5.2] M. A. H. Smith, C. P. Rinsland, B. Fridovich, and K. Narahari Rao, in Molecular Spectroscopy: Modern Research, Vol. III (K. Narahari Rao, ed.), pp. 111–248. Academic Press, San Diego (1985) I.FB.SB.

### 5.2 Microwave Tables and Bibliographies

[5.3] A. G. Maki, Microwave spectra of molecules of astrophysical interest VI. Carbonyl sulfide and hydrogen cyanide, J. Phys. Chem. Ref. Data **3**, 221–244 (1974) F.
[5.4] F. J. Lovas, Microwave spectral tables. I. Diatomic molecules, J. Phys. Chem. Ref. Data **3**, 609–770 (1974) F.
[5.5] F. J. Lovas, Microwave spectral tables. II. Triatomic molecules, J. Phys. Chem. Ref. Data **7**, 1445–1750 (1978) F.

### 5.3 OCS

[5.6] A. Roberts, Rotational spectrum of OC^14^S and the nuclear spin of C^14^, Phys. Rev. **73**, 1405 (1948) F.
[5.7] C. H. Townes, A. N. Holden, and F. R. Merritt, Microwave spectra of some linear XYZ molecules, Phys. Rev. **74**, 1113–1133 (1948) F.
[5.8] M. W. P. Strandberg, T. Wentink, and R. L. Kyhl, Rotational absorption spectrum of OCS, Phys. Rev. **75**, 270–278 (1949) F.
[5.9] W. Low and C. H. Townes, O^17^ and S^36^ in the rotational spectrum of OCS, Phys. Rev. **75**, 529–530 (1949) F.
[5.10] T. Wentink, W. S. Koski, and V. W. Cohen, The Mass of S^35^ from microwave spectroscopy, Phys. Rev. **81**, 948–951 (1951) F.
[5.11] C. M. Johnson, R. Trambarulo, and W. Gordy, Microwave spectroscopy in the region from two to three millimeters, part II, Phys. Rev. **84**, 1178–1180 (1951) F.
[5.12] D. Z. Robinson, The experimental determination of the intensities of infrared absorption bands. IV. Measurements of the stretching vibrations of OCS and CS_2_, J. Chem. Phys. **19**, 881–886 (1951) I.
[5.13] H. J. Callomon, D. C. McKean, and H. W. Thompson, Intensities of vibration bands. IV. Carbonyl sulphide and acetylene, Proc. Roy. Soc. (London) **A208**, 341–351 (1951) I.
[5.14] D. C. McKean, H. J. Callomon, and H. W. Thompson, Intensities of vibration bands of carbonyl sulfide and carbon disulfide, J. Chem. Phys. **20**, 520 (1951) I.
[5.15] S. Geschwind, G. R. Gunther-Mohr, and G. Silvey, The spin and quadrupole moment of O^17^, Phys. Rev. **85**, 474–477 (1952) F.
[5.16] J. R. Eshbach, R. E. Hillger, and M. W. P. Strandberg, The nuclear magnetic moment of S^33^ from microwave spectroscopy, Phys. Rev. **85**, 532–539 (1952) F.
[5.17] S. J. Tetenbaum, Six-millimeter spectra of OCS and N_2_O, Phys. Rev. **88**, 772–774 (1952) F.
[5.18] B. F. Burke, M. W. P. Strandberg, V. W. Cohen, and W. S. Koski, The nuclear magnetic moment of S^35^ by microwave spectroscopy, Phys. Rev. **93**, 193–194 (1954) F.
[5.19] W. C. King and W. Gordy, One-to-two millimeter wave spectroscopy. IV. Experimental methods and results for OCS, CH_3_F, and H_2_O, Phys. Rev. **93**, 407–412 (1954) F.
[5.20] C. A. Burrus and W. Gordy, Submillimeter wave spectroscopy, Phys. Rev. **93**, 897–898 (1954) F.
[5.21] E. C. Wingfield and J. W. Straley, Intensity of the bending mode in carbonyl sulfide, J. Chem. Phys. **22**, 1949–1950 (1954) I.
[5.22] W. Low, Fermi resonance in the microwave spectrum of linear XYZ molecules, Phys. Rev. **97**, 1664–1667 (1955) F.
[5.23] H. C. Allen, E. K. Plyler, and L. R. Blaine, Infrared spectrum of carbonyl sulfide, J. Chem. Phys. **26**, 400–403 (1957) W.
[5.24] A. G Maki, E. K. Plyler, and E. D. Tidwell, Vibration-rotation bands of carbonyl sulfide, J. Res. Natl. Bur. Stand. (U.S.) **66A**, 163–167 (1962) W.
[5.25] E. A. Triaille, Spectres de vibration-rotation a haute resolution du ^16^O^13^C^32^S entre 3650 cm^−1^ et 7000 cm^−1^, Ann. Soc. Scient. Bruxelles **79**, 193–221 (1965) W.
[5.26] E. A. Triaille and C. P. Courtoy, Further evidence for perturbations in the infrared spectra of carbonyl sulfide, J. Mol. Spectrosc. **18**, 118–128 (1965) W.
[5.27] T. F. Deutsch, OCS molecular laser, Appl. Phys. Lett. **8**, 334–335 (1966) W.
[5.28] H. Yamada and W. B. Person, Absolute infrared intensities of some linear triatomic molecules in the gas phase, J. Chem. Phys. **45**, 1861–1865 (1966) I.
[5.29] Y. Morino and C. Matsumura, Microwave spectra and equilibrium structure of carbonyl sulfide, Bull. Chem. Soc. Japan **40**, 1095–1100 (1967) F.
[5.30] R. H. Hill, D. E. Kaplan, G. F. Herrmann, and S. K. Ichiki, Emission microwave spectroscopy: OCS, Phys. Rev. Lett. **18**, 105–107 (1967) F.
[5.31] L. H. Scharpen, J. S. Muenter, and V. W. Laurie, Determination of the polarizability anisotropy of OCS by microwave spectroscopy, J. Chem. Phys. **46**, 2431–2434 (1967) F.
[5.32] A. G Maki, Measurement of the direct *l*-doublet transitions in carbonyl sulfide, J. Mol. Spectrosc. **23**, 110–111 (1967) F.
[5.33] B. T. Berendts and A. Dymanus, Evaluation of molecular quadrupole moments from broadening of microwave spectral lines. I. Measurements, J. Chem. Phys. **48**, 1361–1367 (1968) SB,FB.
[5.34] A. Fayt, Resonance anharmonique du troisieme ordre dans le OCS, Ann. Soc. Scient. Bruxelles **82**, 101–112 (1968) W.
[5.35] Y. Morino and T. Nakagawa, Least-squares determination of the anharmonic potential constants of carbonyl sulfide, J. Mol. Spectrosc. **26**, 496–523 (1968) T,F,W.
[5.36] J. S. Murphy and J. E. Boggs, Collision broadening of ratational absorption lines. III. Broadening by linear molecules and inert gases and the determination of molecular quadrupole moments, J. Chem. Phys. **49**, 3333–3343 (1968) SB,FB.
[5.37] C. C. Costain, The use of saturation dip absorption in microwave spectroscopy and in microwave frequency stabilization, Can. J. Phys. **47**, 2431–2433 (1969) F.
[5.38] L. H. Sharpen, J. S. Muenter, and V. W. Laurie, Electric polarizability anisotropics of nitrous oxide, propyne, and carbonyl sulfide by microwave spectroscopy, J. Chem. Phys. **53**, 2513–2519 (1970) F.
[5.39] A. Fayt, Spectres infrarouges de l’oxysulfure de carbone entre 2400 ct 7000 cm^−1^, Ann. Soc. Scient. Bruxelles **84**, 69–106 (1970) W.
[5.40] F. Meyer-Bourbonneux and C. Meyer, Constantes vibrorotationnelles des trois especes isotopiques OCS^32^, OCS^33^, OCS^34^, Compt. Rend. **270**, 1224–1226 (1970) W.
[5.41] R. S. Winton and W. Gordy, High-precision millimeterwave spectroscopy with the Lamb dip, Phys. Lett. **32A**, 219–220 (1970) F.
[5.42] F. H. De Leeuw and A. Dymanus, Electric and Magnetic Properties of OCS measured by molecular-beam electric-resonance spectroscopy, Chem. Phys. Lett. **7**, 288–292 (1970) F.
[5.43] P. Helminger, F. C. DeLucia, and W. Gordy, Extension of microwave absorption spectroscopy to 0.37-mm wavelength, Phys. Rev. Lett. **25**, 1397–1399 (1970) F.
[5.44] I. C. Story, V. I. Metchnik, and R. W. Parsons, The measurement of the widths and pressure-induced shifts of microwave spectral lines, J. Phys. B 4, 593–608 (1971) PS, SB, FB.
[5.45] A. Fayt and R. Vandenhaute, Spectres infrarouges et analyse vibrorotationnelle du ^16^O^13^C^32^S, Ann. Soc. Scient. Bruxelles **85**, 105–116 (1971) W.
[5.46] A. Fayt, Molecular constants of carbonyl sulfide, Ann. Soc. Scient. Bruxelles **86**, 61–88 (1972) W.
[5.47] J. M. L. J. Reinartz, W. L. Meerts, and A. Dymanus, Electric and magnetic properties of OCS in the (01 ^1^0) vibrational state measured by molecular-beam electric-resonance spectroscopy, Chem. Phys. Lett. **16**, 576–580 (1972) F.
[5.48] A. G. Maki and D. R. Johnson, Microwave spectra of carbonyl sulfide: measurements of ground state and vibrationally excited ^16^O^13^C^32^S, ^18^O^12^C^32^S, and other isotopic species, J. Mol. Spectrosc. **47**, 226–233 (1973) F.
[5.49] A. Foord and D. H. Whiffen, Dipole moment function of carbonyl sulphide, Mol. Phys. **26**, 959–968 (1973) I.
[5.50] H. Taft, P. Bhattacharyya, N. Smith, and B. P. Dailey, High resolution microwave studies of the magnetic properties of OCS, Chem Phys. Lett. **22**, 113–117 (1973) F.
[5.51] R. E. Davis and J. S. Muenter, The rotational magnetic moment for the *J* = 1,2,3,4 and 5 states of OCS, Chem. Phys. Lett. **24**, 343–345 (1974) F.
[5.52] J. M. L. J. Reinartz and A. Dymanus, Molecular constants of OCS isotopes in the (01 ^1^0) vibrational state measured by molecular–beam electric-resonance spectroscopy, Chem. Phys. Lett. **24**, 346–351 (1974) F.
[5.53] M. Bogey, A. Bauer, and S. Maes, Observation of rotational transitions in the *v*_3_ state of OCS through vibrational energy transfer from activated nitrogen, Chem. Phys. Lett. **24**, 516–519 (1974) F.
[5.54] B. Fabricant and J. S. Muenter, *l*-type doubling transitions in *Δ* and *Φ* states of OCS, J. Mol. Spectrosc. **53**, 57–61 (1974) F.
[5.55] N. W. Larsen and B. P. Winnewisser, Millimeter wave rotational transitions of ^16^O^12^C^32^S and ^16^O^13^C^32^S, Z. Naturforsch. A **29**, 1213–1215 (1974) F.
[5.56] M. Bogey, Microwave absorption spectroscopy in the *v*_3_ states of OCS and N_2_O through energy transfer from $N_{2}^{*}$, J. Phys. B **8**, 1934–1938 (1975) F.
[5.57] R. J. Butcher, R. B. Dennis, and S. D. Smith, The tunable spin-flip Raman laser II. Continuous wave molecular spectroscopy, Proc. Roy. Soc. (London) A **344**, 541–561 (1975) W,SB.
[5.58] R. A. Creswell, S. R. Brown, and R. H. Schwendeman, Linewidths in OCS: isotope effects, vibrational effects, temperature dependence, and T_1_/T_2_, J. Chem. Phys. **64**, 1820–1827 (1976) SB.
[5.59] K. A. Davis and J. Overend, The general quartic valence force field of OCS, Spectrochim. Acta A **32**, 233–240 (1976) W,T.
[5.60] A. G. Maki and S. M. Freund, Laser Stark measurements on OCS including the observation of zero-field-forbidden *ΔJ* = 0, ±2 transitions, J. Mol. Spectrosc. **62**, 90–98 (1976) LS.
[5.61] F. Meyer-Bourbonneux, J. Dupre-Maquaire, and C. Meyer, Molecular constants of some levels of the *v*_2_ mode of carbonyl sulfide, J. Mol. Spectrosc. **63**, 288–305 (1976) W.
[5.62] K. Nakagawa, T. Nakagawa, Y. Ueda, and K. Kuchitsu, Laser Stark spectroscopy of OCS in the 9.5 *µ*m region, J. Mol. Spectrosc. **63**, 547–552 (1976) LS.
[5.63] W. R. MacGillivray, The measurement of widths and pressure-induced shifts of rotational lines in the microwave region, J. Phys. B **9**, 2511–2520 (1976) PS, SB.
[5.64] D. H. Whiffen, Computation of centrifugal distortion constants from the force field; example of carbonyl sulphide, Mol. Phys. **31**, 989–1000 (1976) T.
[5.65] A. Fayt and R. Vandenhaute, Infra-red spectra and rovibrational analysis of ^18^O^12^C^32^S, Mol. Phys. **31**, 1861–1873 (1976) W.
[5.66] J. G. Smith, Centrifugal distortion of carbonyl sulphide in excited vibrational states, J. Chem. Soc. Faraday Trans. II **72**, 2298–2300 (1976) F.
[5.67] S. C. M. Luijendijk, On the shape of pressure-broadened absorption lines in the microwave region II. Collision-induced width and shift of some rotational absorption lines as a function of temperature, J. Phys. B **10**, 1741–1747 (1977) PS, SB.
[5.68] P. L. Hewitt, Width and shift measurements in the microwave spectra of NH_3_, CH_3_Cl, and OCS, J. Quant. Spectrosc. Radiat. Transfer **17**, 227–232 (1977) PS, SB.
[5.69] A. Fayt, D. Van Lerberghe, J. P. Kupfer, H. Pascher, and H. G. Hafele, Analysis of vibration-rotation bands of OCS in the 5.3 *µ*m region using a tunable Q-switched spin-flip Raman laser, Mol. Phys. **33**, 603–610 (1977) W.
[5.70] P. G. Buckley, J. H. Carpenter, A. McNeish, J. D. Muse, J. J. Turner, and D. H. Whiffen, High resolution infrared spectroscopy using a spin-flip Raman laser: the bands of carbonyl sulphide near 1890 cm^−1^, J. Chem. Soc. Faraday Trans. II **74**, 129–135 (1978) W.
[5.71] K. Tanaka and T. Tanaka, Laser microwave double resonance spectroscopy of OCS with a 9.4-*µ*m CO_2_ laser—Precise measurement of Dipole moment in the 03^1^0 vibrational state, J. Mol. Spectrosc. **69**, 335–340 (1978) F,LS.
[5.72] F. Herlemont, M. Lyszyk, and J. Lemaire, Infrared spectroscopy of OCS, SO_2_, O_3_ with a CO_2_ waveguide laser, J. Mol. Spectrosc. **77**, 69–75 (1979) F.
[5.73] J. S. Wells, F. R. Petersen, and A. G. Maki, Heterodyne frequency measurements with a tunable diode laser-CO_2_ laser spectrometer: spectroscopic reference frequencies in the 9.5 *µ*m band of carbonyl sulfide, Appl. Opt. **18**, 3567–3573 (1979) F,I.
[5.74] W. G. Mankin, M. T. Coffey, D. W. T. Griffith, and S. R. Drayson, Spectroscopic measurement of carbonyl sulfide (OCS) in the stratosphere, Geophys. Res. Lett. **6**, 853–856 (1979) I,FB.
[5.75] G. Guelachvili, Nombres d’ondes absolus dc la bande *v*_3_ de ^16^O^12^C^32^S par spectroscopie de Fourier, Opt. Commun. **30**, 361–363 (1979) W.
[5.76] G. Blanquet, J. Walrand, and C. P. Courtoy, The *v*_3_ band of ^16^O^12^C^34^S and ^16^O^13^C^34^S, J. Mol. Spectrosc. **81**, 473–479 (1980) W.
[5.77] B. M. Landsberg, New optically pumped cw submillimeter emission lines from OCS CH_3_OH and CH_3_OD, IEEE J. Quant. Elect. **QE-16**, 704–706 (1980) W.
[5.78] M. Bogey and A. Bauer, Microwave spectroscopy of OCS in highly excited vibrational states through energy transfer from N_2_, J. Mol. Spectrosc. **84**, 170–178 (1980) F.
[5.79] A. Dubrulle, J. Demaison, J. Burie, and D. Boucher, The millimeter wave rotational spectra of carbonyl sulfide, Z. Naturforsch. A **35**, 471–474 (1980) F.
[5.80] W. A. Wensink, C. Noorman, and H. A. Dijkerman, Self-shifting of some rotational transitions of OCS and CH_3_CCH (propyne). A survey of measurements on shifting of rotational absorption lines of molecules, J. Phys. B **14**, 2813–2821 (1981) F,PS.
[5.81] M. Sergent-Rozey and Nguyen-Van-Thanh, Pure rotational spectrum of carbonyl sulfide in the far-infrared, Infrared Phys. **21**, 221–224 (1981) W.
[5.82] A. V. Burenin, E. N. Karyakin, A. F. Krupnov, S. M. Shapin, and A. N. Valdov, Submillimeter microwave spectrum and spectroscopic constants of the OCS molecule, J. Mol. Spectrosc. **85**, 1–7 (1981) F.
[5.83] J. S. Wells, F. R. Petersen, A. G. Maki, and D. J. Sukle, Heterodyne frequency measurements on the 11.6-µm band of OCS: new frequency/wavelength calibration tables for 11.6- and 5.8-*µ*m OCS bands, Appl. Opt. **20**, 1676–1684 (1981) and **20**, 2874 (1981) F,I.
[5.84] A. V. Burenin, A. N. Val’dov, E. N. Karyakin, A. F. Krupnov, and S. M. Shapin, Submillimeter microwave spectrum and spectroscopic constants of the OCS molecule, J. Mol. Spectrosc. **87**, 312–315 (1981) F.
[5.85] V. Malathy Devi, P. P. Das, A. Bano, and K. Narahari Rao, Diode laser measurements of strengths of ^16^O^12^C^32^S lines at 12 *µ*m, J. Mol. Spectrosc. **87**, 578–581 (1981) I,SB.
[5.86] Nguyen-van-Thanh, J.-P. Bouanich, I. Rossi, and H. Strapelias, Intensity and dipole correlation functions for the *v*_3_ band of compressed OCS, Can. J. Phys. **59**, 1563–1568 (1981) I,SB.
[5.87] J. S. Wells, F. R. Petersen, A. G. Maki, and D. J. Sukle, Heterodyne frequency measurements (at 11.6 *µ*m) on isotopic species of carbonyl sulfide, OC^34^S,O^13^CS, OC^33^S, ^18^OCS, and O^13^C^14^S, J. Mol. Spectrosc. **89**, 421–429 (1981) F,W.
[5.88] J. P. Sattler, T. L. Worchesky, A. G. Maki, and W. J. Lafferty, Heterodyne frequency measurements on carbonyl sulfide near 1050 cm^−1^, J. Mol. Spectrosc. **90**, 460–466 (1981) F,W.
[5.89] R. H. Kagann, Infrared absorption intensities for OCS, J. Mol. Spectrosc. **94**, 192–198 (1982) I.
[5.90] J. Kauppinen, K. Jolma, and V.-M. Horneman, New wave-number calibration tables for H_2_O, CO_2_, and OCS lines between 500 and 900 cm^−1^, Appl. Opt. **21**, 3332–3336 (1982) W.
[5.91] W. Klebsch, K. Yamada, and G. Winnewisser, Diode laser spectrum of OCS: the *v*_1_ band at 2062 cm^−1^, Z. Naturforsch. A **38**, 157–162 (1983) W.
[5.92] R. P. Leavitt and J. P. Sattler, Pressure Broadening of OCS in the 10 µm region, J. Quant. Spectrosc. Radiat. Transfer **29**, 179–181 (1983) SB.
[5.93] A. Picard-Bersellini, B. J. Whitakcr, and Ph. Brechignac, Rotational inelastic transitions in OCS using a tunable diode laser and a frequency doubled CO_2_ laser, J. Chcm. Phys. **79**, 1556–1557 (1983) SB.
[5.94] J. S. Wells, F. R. Petersen, and A. G. Maki, Heterodyne frequency measurements of carbonyl sulfide transitions at 26 and 51 THz. Improved OCS, O^13^CS, and OC^34^S molecular constants, J. Mol. Spectrosc. **98**, 404–412 (1983) F,W,PS.
[5.95] W. Klebsch, K. Yamada, and G. Winnewisser, Diode laser spectrum of OCS in a dc discharge, J. Mol. Spectrosc. **99**, 479–481 (1983) W.
[5.96] K. Jolma, J. Kauppinen, and V.-M. Horneman, Vibration-rotation bands of CO_2_ and OCS in the region 540–890 cm^−1^, J. Mol. Spectrosc. **101**, 300–305 (1983) W.
[5.97] K. Tanaka, H. Ito, K. Harada, and T. Tanaka, CO_2_ and CO laser microwave double resonance spectroscopy of OCS: precise measurement of dipole moment and polarizability anisotropy, J. Chcm. Phys. **80**, 5893–5905 (1984) LS,F.
[5.98] K. Tanaka, H. Ito, and T. Tanaka, Millimeter wave spectroscopy of OCS in vibrationally excited states, J. Mol. Spectrosc. **107**, 32–332 (1984) F.
[5.99] S. C. Mehrotra and H. Mader, Study of T_2_ relaxation for *l*-doublet transitions of OCS, Can. J. Phys. **62**, 1280–1285 (1984) F,SB.
[5.100] K. Tanaka, T. Tanaka, and I. Suzuki, Dipole moment function of carbonyl sulfide from analysis of precise dipole moments and infrared intensities, J. Chem. Phys. **82**, 2835–2844 (1985) LS,I
[5.101] N. Hunt, S. C Foster, J. W. C. Johns, and A. R. W. McKellar, High-resolution spectroscopy of 16 bands of OCS in the region 1975–2140 cm^−1^ for diode laser calibration, J. Mol. Spectrosc. **111**, 42–53 (1985) W,I.
[5.102] K. Jolma, V.-M. Horneman, J. Kauppinen, and A. G. Maki, Absolute OCS wavenumbers and analysis of bands in the region of the lowest fundamental *v*_2_, J. Mol. Spectrosc. **113**, 167–174 (1985) W.
[5.103] J.-P. Bouanich, G. Blanquet, J. Walrand, and C. P. Courtoy, Diode laser measurements of line strengths and collisional half-widths in the *v*_1_ band of OCS at 298 and 200 K, J. Quant. Spectrosc. Radiat. Transfer **36**, 295–306 (1986) I,SB.
[5.104] M. Dang-Nhu and G. Guclachvili, Intensities des raies d’absorption dans la bandc *v*_2_*+v*_3_ de ^16^O^12^C^32^S, Mol. Phys. **58**, 535–540 (1986) W,I.
[5.105] A. Fayt, R. Vandenhaute, and J.-G. Lahaye, Global rovibrational analysis of carbonyl sulfide, J. Mol. Spectrosc. **119**, 233–266 (1986) F,LS,W.
[5.106] J.-G. Lahaye, R. Vandenhaute, and A. Fayt, CO_2_ laser saturation Stark spectra and global Stark analysis of carbonyl sulfide, J. Mol. Spectrosc. **119**, 267–279 (1986) LS.
[5.107] A. G. Maki, J. S. Wells, and A. Hinz, Heterodyne frequency measurements on the 12^0^0-00^0^0 band of OCS, Int. J. Infrared Millimeter Waves **7**, 909–917 (1986) F.
[5.108] B. Lamalle, P. Suzeau, F. Truchetct, and J. Chanussot, Utilisation d’un asservissement de frequence pour la determination precise des parametres de decalage et d’elargissement de la raic *J*:2→3 d’OCS en function de la pression, Can. J. Phys. **65**, 452–457 (1987) SB.PS.
[5.109] G. Blanquet, F. Dcrie, and J. Walrand, Diode-laser spectrum of isotopic carbonyl sulfide OC^34^S in the region of 847 cm^−1^, J. Mol. Spectrosc. **123**, 14–25 (1987) W.
[5.110] J.-P. Bouanich, J. Walrand, S. Alberty, and G. Blanquet, Diode-laser measurements of oxygen-broadened linewidths in the *v*_1_ band of OCS, J. Mol. Spectrosc. **123**, 37–47 (1987) FB.
[5.111] J.-G. Lahaye, R. Vandenhaute, and A. Fayt, CO_2_ laser saturation Stark spectra and global rovibrational analysis of the main isotopic species of carbonyl sulfide (OC^34^S, O^13^CS, and ^18^OCS), J. Mol. Spectrosc. **123**, 48–83 (1987) LS.
[5.112] L. S. Masukidi, J.-G. Lahaye, B. Coveliers, and A. Fayt, Intracavity CO-laser Stark spectrometer from 2063 to 1228 cm^−1^, J. Opt. Soc. Am. B **4**, 1177–1180 (1987) LS.
[5.113] I. Merke and H. Dreizler, Determination of quadrupole and spin-rotation coupling in the rotational spectrum of carbonylsulfide-^33^S and -^17^O, Z. Naturforsch. A **42**, 1043–1044 (1987) F.
[5.114] F. J. Lovas and R. D. Suenram, Pulsed beam Fourier transform microwave measurements on OCS and rare gas complexes of OCS with Ne, Ar, and Kr, J. Chem. Phys. **87**, 2010–2020 (1987) F.
[5.115] K. M. T. Yamada and W. Klebsch, High-temperature spectrum of OCS in a dc discharge by diode laser spectroscopy, J. Mol. Spectrosc. **125**, 380–392 (1987) W.
[5.116] J.-P. Bouanich, C. Campers, G. Blanquet, and J. Walrand, Diode-laser measurements of Ar- and CO_2_-broadened linewidths in the *v*_1_ band of OCS, J. Quant. Spectrosc. Radiat. Transfer **39**, 353–365 (1988) FB.
[5.117] J.-P. Bouanich and G. Blanquet, Pressure broadening of CO and OCS spectral lines, J. Quant. Spectrosc. Radiat. Transfer **40**, 205–220 (1988) T,SB,FB.
[5.118] J. C. Depannemaecker and J. Lemaire, Measurement with a double-beam spectrometer of strengths and half-widths of 2*v*_2_ and 3*v*_2_–*v*_2_ OCS lines, J. Mol. Spectrosc. **128**, 350–359 (1988) I,SB.
[5.119] J. P. M. De Vreede, M. P. W. Gillis, and H. A. Dijkerman, Linewidth, lineshift, and lineshape measurements on rotational transitions of OCS using frequency modulation, J. Mol. Spectrosc. **128**, 509–520 (1988) F,SB,PS.
[5.120] A. G. Maki, Wm. B. Olson, J. S. Wells, and M. D. Vanek, Heterodyne and FTS measurements on the OCS hot bands near 1890 cm^−1^, J. Mol. Spectrosc. **130**, 69–80 (1988) F,W,PS.
[5.121] M. Schneider, A. G. Maki, M. D. Vanek, and J. S. Wells, Heterodyne measurements on OCS near 1327 cm^−1^, J. Mol. Spectrosc. **134**, 349–353 (1989) F.
[5.122] J. S. Wells, M. D. Vanek, and A. G. Maki, Heterodyne frequency and Fourier transform spectroscopy measurements on OCS near 1700 cm^−1^, J. Mol. Spectrosc. **135**, 84–88 (1989) F,W.
[5.123] J. P. Bouanich, Diode laser measurements of He-broadened linewidths in the *v*_1_ band of OCS, J. Quant. Spectrosc. Radiat. Transfer **42**, 319–326 (1989) FB.
[5.124] M. D. Vanek, D. A. Jennings, J. S. Wells, and A. G. Maki, Frequency measurements of high-*J* rotational transitions of OCS and N_2_O, J. Mol. Spectrosc. **138**, 79–83 (1989) F.
[5.125] J. S. Wells, M. Schneider, and A. G. Maki, Heterodyne frequency measurements on OCS near 61.76 THz (2060 cm^−1^), J. Mol. Spectrosc. **140**, 170–176 (1990) F.
[5.126] A. Fayt, J.-G. Lahaye, J. Lemaire, F. Herlemont, and J. G. Bantegnie, Waveguide and diode heterodyne measurements with CO_2_ laser and assignment of the CW FIR laser emission of OCS, J. Mol. Spectrosc. **140**, 252–258 (1990) F.
[5.127] G. Blanquet, J. Walrand, I. Hilgers, and D. Lambot, Spectral intensities in the *v*_1_ band of carbonyl sulfide and its isotopic species, J. Mol. Spectrosc. **140**, 295–300 (1990) I.
[5.128] A.-M. Tolonen, V.-M. Horneman, and S. Alanko, Absolute OCS wavenumbers and analysis of bands in the region of the overtone 2*v*_2_, J. Mol. Spectrosc. **144**, 18–26 (1990) W.
[5.129] A. G. Maki, J. S. Wells, and D. A. Jennings, Heterodyne frequency measurements on CO and OCS beyond 2100 cm^−1^, J. Mol. Spectrosc. **144**, 224–229 (1990) F.
[5.130] G. Blanquet, J. Walrand, J.-P. Bouanich, and C. Boulet, Line-mixing effects in Ar-broadened doublets of a hot band of OCS, J. Chem. Phys. **93**, 6962–6970 (1990) FB.
[5.131] G. Blanquet, J. Walrand, and J.-P. Bouanich, Diode laser measurements of Kr-broadencd linewidths in the *v*_1_ band of OCS, Appl. Opt. **29**, 5366–5371 (1990) FB.
[5.132] A. G. Maki, J. S. Wells, and J. B. Burkholder, High resolution measurements of the bands of carbonyl sulfide between 2510 and 3150 cm^−1^, J. Mol. Spectrosc. **147**, 173–181 (1991) W,I.
[5.133] M. D. Vanek, J. S. Wells, and A. G. Maki, Pressure dependent lineshift measurements on OCS, J. Mol. Spectrosc. **147**, 398–405 (1991) PS,PB,F.
[5.134] G. Blanquet, P. Coupe, F. Derie, and J. Walrand, Spectral intensities in the $2v_{2}^{0}$ band of carbonyl sulfide, J. Mol. Spectrosc. **147**, 543–545 (1991) I.
[5.135] T. L. Tan and E. C. Looi, FTIR measurements on the 3*v*_2_*–v*_2_ band of carbonyl sulfide, J. Mol. Spectrosc. **148**, 262–264 (1991) W.
[5.136] T. L. Tan, E. C. Looi, and K. T. Lua, Hot-band spectrum of OCS near 850 cm^−1^, J. Mol. Spectrosc. **148**, 265–269 (1991) W.
[5.137] L. S. Masukidi, J. G. Lahaye, and A. Fayt, Intracavity CO laser Stark spectroscopy of the *v*_3_ band of carbonyl sulfide, J. Mol. Spectrosc. **148**, 281–302 (1991) LS.
[5.137a] A. Dax, M. Mürtz, M. Schaefer, M. Schneider, E. M. Bachem, W. Urban, J. S. Wells, and A. Maki, Extension of Heterodyne measurements to 3.3 *μ*m, (to be published) F.

### 5.4 N_2_O

[5.138] D. K. Coles, E. S. Elyash, and J. G. Gorman, Microwave absorption spectra of N_2_O, Phys. Rev. **72**, 973 (1947) F.
[5.139] D. K. Coles and R. H. Hughes, Microwave spectra of nitrous oxide, Phys. Rev. **76**, 178A (1949) F.
[5.140] R. G. Shulman, B. P. Dailey, and C. H. Townes, Molecular dipole moments and Stark effects III. dipole moment determinations, Phys. Rev. **78**, 145–148 (1950) F.
[5.141] H. W. Thompson and R. L. Williams, Vibration-rotation bands of nitrous oxide, Proc. Roy. Soc. (London) A **208**, 326–331 (1951) W.
[5.142] H. J. Callomon, D. C. McKean, and H. W. Thompson, Intensities of vibration bands III. nitrous oxide, Proc. Roy. Soc. (London) A **208**, 332–341 (1951) I.
[5.143] R. M Goody and T. W. Wormell, The quantitative determination of atmospheric gases by infrared spectroscopic methods I. Laboratory determination of the absorption of 7.8 and 8.6 *μ* bands of nitrous oxide with dry air as a foreign gas, Proc. Roy. Soc. (London) A **209**, 178–196 (1951) I,FB.
[5.144] D. F. Eggers and B. L. Crawford, Vibrational intensities. III. Carbon dioxide and nitrous oxide, J. Chem. Phys. **19**, 1554–1561 (1951) I.
[5.145] C. M. Johnson, R. Trambarulo, and W. Gordy, Microwave spectroscopy in the region from two to three millimeters, part II, Phys. Rev. **84**, 1178–1180 (1951) F.
[5.146] S. J. Tetenbaum, Six-millimeter spectra of OCS and N_2_O, Phys. Rev. **88**, 772–774 (1952) F.
[5.147] K. Lakshmi, K. Narahari Rao, and H. H. Nielsen, Molecular constants of nitrous oxide from measurements of *v*_2_ at 17 *μ*, J. Chem. Phys. **24**, 811–813 (1956) W.
[5.148] C. A. Burrus and W. Gordy, Millimeter and submillimeter wave spectroscopy, Phys. Rev. **101**, 599–602 (1956) F.
[5.149] K. Narahari Rao and H. H. Nielsen, Fermi diad 10^0^0 and 02^0^0 of nitrous oxide, Can. J. Phys. **34**, 1147–1152 (1956) W.
[5.150] E. D. Tidwell, E. K. Plyler, and W. S. Benedict, Vibration-rotation bands of N_2_O, J. Opt. Soc. Am. **50**, 1243–1263 (1960) W.
[5.151] D. E. Burch and D. Williams, Total absorptanee of nitrous oxide bands in the infrared, Appl. Opt. **1**, 473–482 (1962) I.
[5.152] P. E. Fraley, W. W. Brimm, and K. Narahari Rao, Vibration-rotation bands of N^4^O^16^ at 4.5 *μ*, J. Mol. Spectrosc. **9**, 487–493 (1962) W.
[5.153] K. Narahari Rao, R. V. deVore, and E. K. Plyler, Wavelength calibrations in the far infrared (30 to 1000 microns), J. Res. Natl. Bur. Stand. (U.S.) **67A**, 351–358 (1963) W.
[5.154] E. K. Plyler, E. D. Tidwell, and A. G. Maki, Infrared absorption spectrum of nitrous oxide (N_2_O) from 1830 cm^−1^ to 2270 cm^−1^, J. Res. Natl. Bur. Stand. (U.S.) **68A**, 79–86 (1964) W.
[5.155] R. P. Grosso and T. K. McCubbin, The *v*_1_–2*v*_2_ Fermi diads of ^14^N_2_^16^O, ^15^N^14^N^16^O, and ^15^N_2_O, J. Mol. Spectrosc. **13**, 240–255 (1964) W.
[5.156] W. J. Lafferty and D. R. Lide, Rotational constants of excited states of ^14^N_2_O, J. Mol. Spectrosc. **14**, 407–408 (1964) F.
[5.157] H. Yamada and W. B. Person, Absolute infrared intensities of some linear triatomic molecules in the gas phase, J. Chem. Phys. **45**, 1861–1865 (1966) I.
[5.158] B. T. Berendts and A. Dymanus, Evaluation of molecular quadrupole moments from broadening of microwave spectral lines. I. Measurements, J. Chem. Phys. **48**, 1361–1367 (1968) SB,FB.
[5.159] I. P. French and T. E. Arnold, Foreigngas broadening of the *J* = 5→6 rotational transition of nitrous oxide, J. Mol. Spectrosc. **27**, 218–224 (1968) SB, FB.
[5.160] J. S. Murphy and J. E. Boggs, Collision broadening of rotational absorption lines. III. Broadening by linear molecules and inert gases and the determination of molecular quadrupole moments, J. Chem. Phys. **49**, 3333–3343 (1968) SB,FB.
[5.161] R. Pearson, T. Sullivan, and L. Frenkel, Microwave spectrum and molecular parameters for ^14^N_2_^16^O, J. Mol. Spectrosc. **34**, 440–449 (1970) F.
[5.162] L. H. Sharpen, J. S. Muenter, and V. W. Laurie, Electric polarizability anisotropics of nitrous oxide, propyne, and carbonyl sulfide by microwave spectroscopy, J. Chem. Phys. **53**, 2513–2519 (1970) F.
[5.163] J. Lemaire, J. Houriez, J. Thibault, and B. Maillard, Double irradiation des molecules de bromure de methyle et de protoxyde d’azote par rayonnements infrarouge et Hertzien, J. Phys. (Paris) **32**, 35–40 (1971) F.
[5.164] L. D. Gray Young, Calculation of the partition function for ^14^N_2_^16^O, J. Quant. Spectrosc. Radiat. Transfer **11**, 1265–1270 (1971) T.
[5.165] G. D. T. Tejwani and P. Varanasi, Theoretical line widths in N_2_O-N_2_O and N_2_O-air collisions, J. Quant. Spectrosc. Radiat. Transfer **11**, 1659–1664 (1971) T.
[5.166] R. A. Toth, Line strengths on N_2_O in the 2.9 micron region, J. Mol. Spectrosc. **40**, 588–604 (1971) I.
[5.167] R. A. Toth, Self-broadened and N_2_ broadened linewidths of N_2_O, J. Mol. Spectrosc. **40**, 605–615 (1971) SB,FB.
[5.168] C. L. Tien, M. F. Modest, and C. R. McCreight, Infrared radiation properties of nitrous oxide, J. Quant. Spectrosc. Radiat. Transfer **12**, 267–277 (1972) I.
[5.169] L. D. Gray Young, Relative intensity calculations for nitrous oxide, J. Quant. Spectrosc. Radiat. Transfer **12**, 307–322 (1972) I,T.
[5.170] J. S. Margolis, Intensity and half width measurements of the (00^0^2-00^0^0) band of N_2_O, J. Quant. Spectrosc. Radiat. Transfer **12**, 751–757 (1972) I,SB,FB.
[5.171] J. E. Lowder, Band intensity and line half-width measurements in N_2_O near 4.5 *μ*, J. Quant. Spectrosc. Radiat. Transfer **12**, 873–880 (1972) I,FB.
[5.172] N. Lacome, C. Boulet, and E. Arie, Spectroscopie par source laser. III. Intensities et largeurs des raies de la transition 00^0^1-10^0^0 du protoxide d’azote. Ecarts a la forme de Lorentz, Can. J. Phys. **51**, 302–310 (1973) I,SB.
[5.173] J. Walrand, G. Blanquet, and C. P. Courtoy, Spectres infrarouge a haute resolution du protoxyde d’azote ^14^N^13^N^16^O etude de la band *v*_3_, Ann. Soc. Scient. Bruxelles **87**, 409–419 (1973) W.
[5.174] L. D. Tubbs and D. Williams, Broadening of infrared absorption lines at reduced temperatures, III. Nitrous oxide, J. Opt. Soc. Am. **63**, 859–863 (1973) I,SB.
[5.175] R. Farrenq and J. Dupre-Maquaire, Vibrational luminescence of N_2_O excited by dc discharge—rotation-vibration constants, J. Mol. Spectrosc. **49**, 268–279 (1974) W.
[5.176] R. Farrenq, D. Gaultier, and C. Rossetti, Vibrational luminescence of N_2_O excited by dc discharge—emission lines intensities and populations of rotational-vibrational levels, J. Mol. Spectrosc. **49**, 280–288 (1974) I.
[5.177] N. Lacome, Line shape in the 00^0^1-10^0^0 band of nitrous oxide and carbon dioxide: contribution of the hot band O1^1^1-11^1^O to its determination, Can. J. Phys. **52**, 470–471 (1974) I,SB.
[5.178] P. Varanase and B. P. Bangaru, Measurement of line intensities of linear molecules under low resolution, J. Quant. Spectrosc. Radiat. Transfer **14**, 1253–1257 (1974) I.
[5.179] A. V. Burenin, A. N. Valbov, L. I. Gershtein, E. N. Karyakin, A. F. Krupnov, A. V. Maslovskii, and S. M. Shchapin, Submillimeter spectrum and intermolecular parameters, Opt. Spectrosc. **37**, 676–678 (1974) F.
[5.180] C. Amiot and G. Guelachvili, Vibration-rotation bands of ^14^N_2_O: 1.2 micron-3.3 micron region, J. Mol. Spectrosc. **51**, 475–491 (1974) W.
[5.181] J. M. Krell and R. L. Sams, Vibration-rotation bands of nitrous oxide: 4.1 micron region, J. Mol. Spectrosc. **51**, 492–507 (1974) W.
[5.182] K. H. Casleton and S. G. Kukolich, Beam maser measurements of hyperfine structure in ^14^N_2_O, J. Chem. Phys. **62**, 2696–2699 (1975) F.
[5.183] B. G. Whitford, K. J. Siemsen, H. D. Rieeius, and G. R. Hanes, Absolute frequency measurements of N_2_O laser transitions, Opt. Commun. **14**, 70–74 (1975) F.
[5.184] G. Blanquet, J. Walrand, and C. P. Courtoy, Bandes de vibration-rotation de formes isotopiques de N_2_O dans la region de 4.5 *μ*m, Ann. Soc. Scient. Bruxelles **89**, 93–114 (1975) W.
[5.185] R. A. Toth and C. B. Farmer, Line strengths of H_2_O and N_2_O in the 1900 cm^−1^ region, J. Mol. Spectrosc. **55** 182–191 (1975) I.
[5.186] M. Bogey, Microwave absorption spectroscopy in the *v*_3_ states of OCS and N_2_O through energy transfer from $N_{2}^{*}$, J. Phys. B 8, 1934–1938 (1975) F.
[5.187] J. P. Boissy, A. Valentin, P. Cardinet, M. L. Claude, and A. Henry, Line intensities of the *v*_3_ fundamental band of nitrous oxide, J. Mol. Spectrosc. **57**, 391–396 (1975) I.
[5.188] J. Perrizo, L. A. Pugh, and K. Narahari Rao, Bands of nitrous oxide at 5.3 *μ*m, J. Mol. Spectrosc. **57**, 397–401 (1975) W.
[5.189] J. Dupre-Maquaire and P. Pinson, Emission spectrum of N_2_O in the 8 μm range, J. Mol. Spectrosc. **58**, 239–249 (1975) W.
[5.190] A. Valentin, M.-F. Le Moal, P. Cardinet, and J.-P. Boissy, High precision spectrum of N_2_O at 4.5 *μ*m and determination of molecular constants for the *v*_3_ and the (v_2_*+v*_3_*v − v*_2_) bands, J. Mol. Spectrosc. **59**, 96–102 (1976) W.
[5.191] C. Amiot and G. Guelachvili, Extension of the 10^6^ samples Fourier spectrometry to the indium anti-monide region: vibration-rotation bands of ^14^N_2_^16^O: 3.3–5.5 *μ*m region, J. Mol. Spectrosc. **59**, 171–190 (1976) W.
[5.192] C. Amiot, Vibration-rotation bands of ^14^N^15^N^16^O-^15^N^14^N^16^O: 1.6–5.7 *μ*m region, J. Mol. Spectrosc. **59**, 191–208 (1976) W.
[5.193] C. Amiot, Vibration-rotation bands of ^15^N_2_^16^O-^14^N_2_^18^O, J. Mol. Spectrosc. **59**, 380–395 (1976) W.
[5.194] B. A. Andreev, A. V. Burenin, E. N. Karyakin, A. F. Krupnov, and S. M. Shapin, Submillimeter wave spectrum and molecular constants of N_2_O, J. Mol. Spectrosc. **62**, 125–148 (1976) F.
[5.195] A. Chedin, C. Amiot, and Z. Cihla, The potential energy function of the nitrous oxide molecule using pure vibrational data, J. Mol. Spectrosc. **63**, 348–369 (1976) T.
[5.196] K. Siemsen and J. Reid, New N_2_O laser band in the 10 *μ*m wavelength region, Opt. Commun. **20**, 284–288 (1977) F.
[5.197] T. Kunitomo, S. Ueoka, H. Masuzaki, K. Utsunomiya, and M. Osumi, Emissivity and band-model parameters for infrared bands of nitrous oxide, J. Quant. Spectrosc. Radiat. Transfer **18**, 405–417 (1977) I.
[5.198] P. Varanasi and F. K. Ko, Intensity and transmission measurements in the *v*_3_ fundamental of N_2_O at low temperatures, J. Quant. Spectrosc. Radiat. Transfer **18**, 465–470 (1977) I.
[5.199] N. Lacome and A. Levy, A parametric deconvolution method: application to two bands of N_2_O in the 1.9-μm region, J. Mol. Spectrosc. **71**, 175–192 (1978) I,SB.
[5.200] W. B. Person and K. C. Kim, Measurements of some line positions and strengths in the *v*_4_ region of SF_6_, J. Chem. Phys. **69**, 1764–1769 (1978) I.
[5.201] W. A. Wensink, C. Noorman, and H. A. Dijkerman, Self-broadening and self-shifting of some rotational transitions of CF_3_H and N_2_O, J. Phys. B **12**, 1687–1699 (1979) SB,PS.
[5.202] K. G. P. Sulzmann, J. M. Kline, and S. S. Penner, Empirical determinations of the effective absorption coefficients for the NO *γ*-bands at 2259 A and the *v*_3_ fundamental of N_2_O at 4.52 μ, J. Quant. Spectrosc. Radiat. Transfer **21**, 475–482 (1979) I.
[5.203] M. J. Reisfeld and H. Flicker, The *v*_2_ band of N_2_O as a frequency standard in the 17-*μ*m region of the infrared, Appl. Opt. **18**, 1136–1138 (1979) W.
[5.204] N. Lacome and A. Levy, High-pressure absorption spectrum of N_2_O in the 2 μm region, Mol. Phys. **39**, 1221–1232 (1980) I,SB,T.
[5.205] I. Suzuki, Dipole moment functions of carbon dioxide and nitrous oxide, J. Mol. Spectrosc. **80**, 12–22 (1980) I,T.
[5.206] Nguyen-Van-Thanh, J.-P. Bouanich, and I. Rossi, The *v*_3_ and 2*v*_3_ IR bandshapes and dipole correlation functions of compressed gaseous N_2_O; intensity measurements of the 2*v*_3_ band, Mol. Phys. **40**, 869–S81 (1980) I.
[5.207] D. B. Braund, A. R. H. Cole, J. A. Cugley, F. R. Honey, R. E. Pulfrey, and G. D. Reece, Precise measurements with a compact vacuum infrared spectrometer, Appl. Opt. **19**, 2146–2152 (1980) W.
[5.208] N. Lacome and A. Levy, Line strengths and self-broadening linewidths of N_2_O in the 2-μm region −24^0^0-00^0^0 and 01^1^2-00^0^0 transitions, J. Mol. Spectrosc. **85**, 205–214 (1981) I,SB.
[5.209] M. O. Bulanin, V. P. Bulychev, and E. B. Khodos, Determination of the parameters of the vibrational-rotational lines of the 00^0^1-10^0^0 band of nitrous oxide, Opt. Spectrosc. (USSR) **56**, 595–597 (1982) I,SB,FB.
[5.210] W. B. Olson, A. G. Maki, and W. J. Lafferty, Tables of N_2_O absorption lines for the calibration of tunable infrared lasers from 522 cm^−1^ to 657 cm^−1^ and from 1115 cm^−1^ to 1340 cm^−1^, J. Phys. Chem. Ref. Data **10**, 1065–1084 (1981) W.
[5.211] Da-Wun Chen, E. R. Niple, and S. K. Poultney, Determining tunable diode laser spectrometer performance through measurement of N_2_O line intensities and widths at 7.8 *μ*m, Appl. Opt. **21**, 2906–2911 (1982) I,SB,FB.
[5.212] C. P. Rinsland, A. Goldman, F. J. Mureray, D. G. Murcray, M. A. H. Smith, R. K. Seals, J. C. Larsen, and P. L. Rinsland, Stratospherie N_2_O mixing ratio profile from high-resolution balloon-borne solar absorption spectra and laboratory speetra near 1880 cm^−1^, Appl. Opt. **21**, 4351–4355 (1982) I.
[5.213] G. Guelaehvili, Absolute N_2_O wavenumbers between 1118 and 1343 cm^−1^ by Fourier transform spectroscopy, Can. J. Phys. **60**, 1334–1347 (1982) W.
[5.214] R. H. Kagann, Infrared absorption intensities for N_2_O, J. Mol. Spectrose. **95**, 297–305 (1982) I.
[5.215] K. Jolma, J. Kauppinen, and V.-M. Horneman, Vibration-rotation spectrum of N_2_O in the region of the lowest fundamental *v*_2_, J. Mol. Spectrose. **101**, 278–284 (1983) W.
[5.216] A. Levy, N. Lacome, and G. Guelaehvili, Measurement of N_2_O line strengths from high-resolution Fourier transform spectra, J. Mol. Spectrose. **103**, 160–175 (1984) I.
[5.217] N. Lacome, A. Levy, and G. Guelaehvili, Fourier transform measurement of self-, N_2_-, and O_2_-broadening of N_2_O lines: temperature dependence of linewidths, Appl. Opt. **23**, 425–435 (1984) SB,FB.
[5.218] R. A. Toth, Line strengths of N_2_O in the 1120–1440 cm^−1^ region, Appl. Opt. **23**, 1825–1834 (1984) I.
[5.219] C. R. Pollock, F. R. Petersen, D. A. Jennings, J. S. Wells, and A. G. Maki, Absolute frequency measurements of the 00^0^2-00^0^0, 20^0^1-00^0^0, and 12^0^1-00^0^0 bands of N_2_O by heterodyne spectroscopy, J. Mol. Spectrose. **107**, 62–71 (1984) F,PS.
[5.220] L. R. Brown and R. A. Toth, Comparison of the frequencies of NH_3_, CO_2_, H_2_O, N_2_O, CO, and CH_4_ as infrared calibration standards, J. Opt. Soc. Am. B **2**, 842–856 (1985) W.
[5.221] J. S. Wells, D. A. Jennings, A. Hinz, J. S. Murray, and A. G. Maki, Heterodyne frequency measurements on N_2_O at 5.3 and 9.0 *μ*m, J. Opt. Soc Am. B **2**, 857–861 (1985) F.
[5.222] M. Margottin-Maclou, P. Dahoo, A. Henry, and L. Henry, Self-broadening parameters in the *v*_3_ band of ^14^N_2_^16^O, J. Mol. Spectrose. **111**, 275–290 (1985) I, SB.
[5.223] A. Henry, M. Margottin-Maclou, and N. Lacome, N_2_-and O_2_-Broadening parameters in the *v*_3_ band of ^14^N_2_^16^O, J. Mol. Spectrose. **111**, 291–300 (1985) FB.
[5.224] J. S. Wells, A. Hinz, and A. G. Maki, Heterodyne frequency measurements on N_2_O between 1257 and 1340 cm^−1^, J. Mol. Spectrose. **114**, 84–96 (1985) F.
[5.225] R. A. Toth, Frequencies of N_2_O in the 1100- to 1440-cm^−1^ region, J. Opt. Soc. Am. B **3**, 1263–1281 (1986) W.
[5.226] M. Loewenstein, J. R. Podolske, T. E. Blackburn, and P. Varanasi, Diode laser Measurements of line strengths and widths in the 4.5-μm bands of N_2_O, J. Quant. Spectrose. Radiat. Transfer **35**, 231–235 (1986) I,FB.
[5.227] A. Bauer, J. L. Teffo, A. Valentin, and T. K. McCubbin, The ground state rotational constants of ^15^N^15^N^16^O, J Mol Spectrose. **120**, 449–454 (1986) F,W.
[5.228] M. Kobayashi and I. Suzuki, Dipole moment function of nitrous oxide, J. Mol. Spectrose. **122**, 157–170 (1987) I,T
[5.229] R. A. Toth, N_2_O vibration-rotation parameters derived from measurements in the 900–1090- and 1580–2380-cm^−1^ regions, J. Opt. Soc. Am. B **4**, 357–374 (1987) W.
[5.230] L. R. Zink, J. S. Wells, and A. G. Maki, Heterodyne frequency measurements on N_2_O near 1060 cm^−1^, J. Mol. Spectrosc. **123**, 426–433 (1987) F.
[5.231] A. Hinz, J. S. Wells, and A. G. Maki, Heterodyne measurements of hot bands and isotopie transitions of N_2_O near 7.8 *μ*m, Z. Phys. D **5**, 351–358 (1987) F.
[5.232] J. M. Colmont and N. Semmoud-Monnanteuil, Pressure broadening of the N_2_O *J* = 9←8 rotational transition by N_2_O, N_2_, and O_2_, J. Mol. Speetrose. **126**, 240–242 (1987) SB,FB.
[5.233] M. P. Esplin, W. M. Barowy, R. J. Huppi, and G. A. Vanasse, High resolution Fourier spectroscopy of nitrous oxide at elevated temperatures, Mikrochim. Aeta (Wien) 403–407 (1988) W.
[5.234] P. Varanasi, Measurement of the absolute intensities of the 7.8 and 8.6 μm band systems of N_2_O, J. Quant. Speetrose. Radiat. Transfer **39**, 189–191 (1988) I.
[5.235] L. L. Straw and A. S. Pine, Q-branch line mixing in N_2_O: Effcets of *l*-type doubling, J. Chem. Phys. **89**, 1427–1434 (1988) I,SB,FB.
[5.236] V. V. Zhurov, V. V. Kovtun, and N. N. Kudryavtsev, Measurement of the intensities and collisional broadening of rotational lines of the 4.5 *μ*m band of N_2_O with the help of a tunable semiconductor laser, J. Appl. Spectrose. **50**, 151–156 (1989) I,SB.
[5.237] Lai-Wa Tang, S. Nadler, and S. J. Daunt, Tunable diode laser measurements of absolute line strengths in the 2*v*_2_ band of N_2_O near 8 μm, J. Quant. Spectrose. Radiat. Transfer **41**, 97–101 (1989) I.
[5.238] P. Varanasi and S. Chudamani, Measurement of pressure-induced shifts of infrared lines with a tunable diode laser, J. Quant. Speetrose. Radiat. Transfer **41**, 173–176 (1989) PS.
[5.239] P. Varanasi and S. Chudamani, Tunable diode laser measurements of line widths in the *v*_1_-fundamental band of ^14^N_2_^16^O at atmospheric temperatures, J. Quant. Spectrose. Radiat. Transfer **41**, 351–357 (1989) FB.
[5.240] P. Varanasi and S. Chudamani, Line strength measurements in the *v*_1_-fundamental band of ^14^N_2_^16^O using a tunable diode laser, J. Quant. Spectrose. Radiat. Transfer **41**, 359–362 (1989) I.
[5.241] M. D. Vanek, M. Schneider, J. S. Wells, and A. G. Maki, Heterodyne measurements on N_2_O near 1635 cm^−1^, J. Mol. Spectrose. **134**, 154–158 (1989) F.
[5.242] M. D. Vanek, D. A. Jennings, J. S. Wells, and A. G. Maki, Frequency measurements of high-*J* rotational transitions of OCS and N_2_O, J. Mol. Spectrose. **138**, 79–83 (1989) F.
[5.243] A. G. Maki, J. S. Wells, and M. D. Vanek, Heterodyne frequency measurements on N_2_O near 930 cm^−1^, J. Mol. Spectrose. **138**, 84–88 (1989) F.
[5.244] M. Margottin-Maelou, A. Henry, and A. Valentin, Line mixing effects in the self- and N_2_-broadened Q-branch of the *v*_2_*+v*_3_ band of N_2_O, Appl. Opt. **28**, 4920–4923 (1989) SB,FB.
[5.245] K. M. T. Yamada, Pure rotation spectrum of NNO in the far infrared region, Z. Naturforsch. **A45**, 837–838 (1990) W.

### 5.5 CO

[5.246] O. R. Gilliam, C. M. Johnson, and W. Gordy, Microwave spectroscopy in the region from two- to three millimeters, Phys. Rev. **84**, 140–144 (1950) F.
[5.247] C. M. Johnson, R. Trambarulo, and W. Gordy, Microwave spectroscopy in the region from two to three millimeters, part II, Phys. Rev. **84**, 1178–1180 (1951) F.
[5.248] I. M. Mills and H. W. Thompson, The fundamental vibration-rotation bands of ^13^C^16^O and ^12^C^18^O, Trans. Faraday Soc. **49**, 224–227 (1953) W.
[5.249] E. K. Plyler, L. R. Blaine, and W. S. Connor, Velocity of light from the molecular constants of carbon monoxide, J. Opt. Soc. Am. **45**, 102–106 (1955) W.
[5.250] W. Gordy and M. J. Cowan, Precision measurements of millimeter and submillimeter wave lines of CO, Bull. Amer. Phys. Soc. **2**, 212–213 (1957) F.
[5.251] B. Rosenblum and A. H. Nethercot, Quadrupole coupling constant and molecular structure of CO^17^, J. Chem. Phys. **27**, 828–829 (1957)F.
[5.252] D. H. Rank, A. H. Guenther, G. D. Saksena, J. N. Shearer, and T. A. Wiggins, Tertiary interferometric wavelength standards from measurements on lines of the 2-0 band of carbon monoxide and derived wavelength standards for some lines of the 1-0 band of carbon monoxide. The velocity of light derived from a band spectrum method. IV, J. Opt. Soc. Am. **47**, 686–689 (1957) W.
[5.253] B. Rosenblum, A. H. Nethercot, and C. H. Townes, Isotopic mass ratios, magnetic moments and sign of the electric dipole moment of carbon monoxide, Phys. Rev. **109**, 400–412 (1958) F.
[5.254] C. A. Burrus, Stark effect from 1.1 to 2.6 millimeters wavelength: PH_3_, PD_3_, DI, and CO, J. Chem. Phys. **28**, 427–429 (1958) F.
[5.255] W. S. Benedict, R. Herman, G. E. Moore, and S. Silverman, The strengths, widths, and shapes of lines in the vibration-rotation bands of CO, Astrophys. J. **135**, 277–297 (1962) I,SB,FB.
[5.256] J. H. Shaw and J. T. Houghton, Total band absorptancc of CO near 4.7 *μ*. Appl. Opt. **3**, 773–779 (1964) I,SB,FB.
[5.257] D. H. Rank, A. G. St. Pierre, and T. A. Wiggins, Rotational and vibration constants of CO, J. Mol. Spectrosc. **18**, 418–427 (1965) W.
[5.258] J. M. Weinberg, E. S. Fishburne, and K. Narahari Rao, Hot bands of CO at 4.7 microns measured to high *J* values, J. Mol. Spectrosc. **18**, 428–442 (1965) W.
[5.259] L. A. Young and W. J. Eachus, Dipole moment function and vibration-rotation matrix elements for CO, J. Chem. Phys. **44**, 4195–4206 (1966) I,T.
[5.260] W. H. Flygare and V. W. Weiss, ^13^C spin-rotation interaction and magnetic shielding in the carbon and oxygen nuclei in formaldehyde, J. Chem. Phys. **45**, 2785–2792 (1966) F.
[5.261] L. Hochard-Demolliere, Mesure de la dispersion dans la bande fondamentale de I’oxyde de carbone, J. Phys. (Paris) **27**, 341–344 (1966) I.
[5.262] J.-P. Bouanich, A. Levy, and C. Haeusler, Constantes Moleculaires de l’oxide de carbone, J. Phys. (Paris) **29**, 641–645 (1968) W,SB,I.
[5.263] C. L. Korb, R. H. Hunt, and E. K. Plyler, Measurement of line strengths at low pressures—Application to the 2-0 band of CO, J. Chem. Phys. **48**, 4252–4260 (1968) I.
[5.264] I. Ozier, L. M. Crapo, and N. F. Ramsey, Spin rotation constant and rotational magnetic moment of ^13^C^16^O, J. Chem. Phys. **49**, 2314–2321 (1968) F.
[5.265] R. A. Toth, R. H. Hunt, and E. K. Plyler, Line intensities in the 3-0 band of CO and dipole moment matrix elements for the CO molecule, J. Mol. Spectrosc. **32**, 85–96 (1969) I,T.
[5.266] P. Helminger, F. C. DcLucia, and W. Gordy, Extension of microwave absorption spectroscopy to 0.37-mm wavelength, Phys. Rev. Lett. **25**, 1397–1399 (1970) F.
[5.267] J.-P. Bouanich, Determination experimental des largcurs et des deplacements des raies de la bande 0→2 dc CO perturbe par Ies gaz rares (He, Ne, Ar, Kr, Xc), J. Quant. Spectrosc. Radiat. Transfer **12**, 1609–1615 (1972) FB,PS.
[5.268] J.-P. Bouanich and C. Brodbeck, Mesure des largcurs et des deplacements des raies de la bande 0→2 de CO autoperturbe et perturbe par N_2_, O_2_, H_2_, HCl, NO, et CO_2_, J. Quant. Spectrosc. Radiat. Transfer **13**, 1–7 (1973) SB,FB,PS.
[5.269] G. Guelachvili, New near infrared wavenumber standards (2←0 band of ^12^C^16^O) by high resolution Fourier spectroscopy in vacuum, Opt. Commun. **8**, 171–175 (1973) W.
[5.270] J.-P. Bouanich and C. Brodbeck, Etalonnage et deplacement des raies de vibration-rotation des bands 0→2 ct 0→3 de I’oxyde de carbone, Rev. Phys. Appl. **9**, 475–478 (1974) PS.
[5.271] J.-P. Bouanich and C. Brodbeck, Moments de transition vibrationnelle des molecules diatomiques. Intensite des raies rovibrationnelles des bandes 0→2 et 0→3 et fonction dipolaire de CO, J. Quant. Spectrosc. Radiat. Transfer **14**, 1199–1208 (1974) I,T.
[5.272] A. W. Mantz and J.-P. Maillard, Emission spectra with a high resolution Fourier transform spectrometer: CO spectra and their astrophysical importance, J. Mol. Spectrosc. **53**, 466–478 (1974) W.
[5.266] P. Helminger, F. C. DcLucia, and W. Gordy, Extension of microwave absorption spectroscopy to 0.37-mm wavelength, Phys. Rev. Lett. **25**, 1397–1399 (1970) F.
[5.267] J.-P. Bouanich, Determination experimental des largcurs et des deplacements des raies de la bande 0→2 de CO perturbe par les gaz rares (He, Ne, Ar, Kr, Xe), J. Quant. Spectrosc. Radiat. Transfer **12**, 1609–1615 (1972) FB,PS.
[5.268] J.-P. Bouanich and C. Brodbeck, Mesure des largeurs et des deplacements des raies de la bande 0→2 de CO autoperturbe et perturbe par N_2_, O_2_, H_2_, HCl, NO, et CO_2_, J. Quant. Spectrosc. Radiat. Transfer **13**, 1–7 (1973) SB,FB,PS.
[5.269] G. Guelachvili, New near infrared wavenumber standards (2←0 band of ^12^C^16^O) by high resolution Fourier spectroscopy in vacuum, Opt. Commun. **8**, 171–175 (1973) W.
[5.270] J.-P. Bouanich and C. Brodbeck, Etalonnage et deplacement des raies de vibration-rotation des bands 0→2 et 0→3 de I’oxyde de carbone, Rev. Phys. Appl. **9**, 475–478 (1974) PS.
[5.271] J.-P. Bouanich and C. Brodbeek, Moments de transition vibrationnelle des molecules diatomiques. Intensite des raies rovibrationnelles des bandes 0→2 et 0→3 et fonction dipolaire de CO, J. Quant. Spectrosc. Radiat. Transfer **14**, 1199–1208 (1974) I,T.
[5.272] A. W. Mantz and J.-P. Maillard, Emission spectra with a high resolution Fourier transform spectrometer: CO spectra and their astrophysical importance, J. Mol. Spectrosc. **53**, 466–478 (1974) W.
[5.273] M. Crance and J. Verges, Measurements of line strengths in the 2-0 band of CO, J. Phys. B **8**, 3001–3006 (1975) I.
[5.274] A. W. Mantz, J.-P. Maillard, W. B. Roh, and K. Narahari Rao, Ground state molecular constants of ^12^C^16^O, J. Mol. Spectrosc. **57**, 155–159 (1975) W.
[5.275] K. Tanabe, Infrared intensity measurement of gas phase molecules, J. Mol. Struct. **29**, 319–327 (1975) I.
[5.276] N. I. Moskalenko, Measurement of intensities and half-widths of spectral absorption lines of the fundamental 0-1 band of CO, Opt. Spectrosc. (USSR) **38**, 382–384 (1975) I,SB,FB.
[5.277] P. Varanasi and S. Sarangi, Measurements of intensities and nitrogen-broadened linewidths in the CO fundamental at low temperature, J. Quant. Spectrosc. Radiat. Transfer **15**, 473–482 (1975) I,FB.
[5.278] R. B. Nerf and M. A. Sonnenberg, Pressure broadening of the *J* = l←0 transition of carbon monoxide, J. Mol. Spectrosc. **58**, 474–478 (1975) SB,FB,PS.
[5.279] J. W. Fleming, Line strength and halfwidth measurements from far infrared absorption spectra: carbon monoxide, J. Quant. Spectrosc. Radiat. Transfer **16**, 63–68 (1976) SB,I.
[5.280] J. P. Bouanich and C. Brodbeck, Vibration-rotation matrix elements for diatomic molecules; vibration-rotation interaction functions $F_{v}^{v^{\prime }}(m)$ for CO, J. Quant. Spectrosc. Radiat. Transfer **16**, 153–163 (1976) I,T.
[5.281] J. Bonamy and D. Robert, Atom-atom potential in rotational line broadening for molecular gases: application to CO lines broadened by CO, N_2_, O_2_, and NO, J. Quant. Spectrosc. Radiat. Transfer **16**, 185–190 (1976) FB.SB.T.
[5.282] R. H. Tipping, Vibration-rotation intensities for hot bands, J. Mol. Spectrosc. **61**, 272–281 (1976) I,T.
[5.283] J. P. Bouanich, High order vibrational matrix elements for diatomic molecules; dipole moment function and transition moments of CO, J. Quant. Spectrosc. Radiat. Transfer. **16**, 1119–1131 (1976) T,I.
[5.284] T. R. Todd, C. M. Clayton, W. B. Telfair, T. K. McCubbin, and J. Pliva, Infrared emission of ^12^C^16^O, ^13^C^16^0, and ^12^C^18^O, J. Mol. Spectrosc. **62**, 201–227 (1976) W.
[5.285] G. Guelachvili, Atomic Masses and Fundamental Constants, (J. H. Sanders and A. H. Wapstra, Eds.), Vol. 5, pp. 424–430, Plenum Press, New York, 1976 W.
[5.286] W. L. Mcerts, F. H. DeLecuw. and A. Dymanus, Electric and magnetic properties of carbon monoxide by molecular-beam electric-resonance spectroscopy, Chem. Phys. 22, 319–324 (1977) F.
[5.287] J. P. Bouanich, Fourth-order contributions to the rotational-vibrational matrix elements for diatomic molecules; application to the $F_{v}^{v^{\prime }}$ functions for CO, J. Quant. Spectrosc. Radiat. Transfer **17**, 639–650 (1977) T,I.
[5.288] T. A. Dixon, Ph.D. thesis, University of Wisconsin, 1977 F.
[5.289] G. Guelachvili, Absolute wavenumbers and molecular constants of the fundamental bands of ^12^C^16^O, ^12^C^17^O, ^12^C^18^O,^13^C^16^O,^13^C^17^O,^13^C^1B^O and of the 2-1 bands of ^12^C^16^O and ^13^C^16^O, around 5 *μ*m, by Fourier spectroscopy under vacuum, J. Mol. Spectrosc. **75**, 251–269 (1979) W.
[5.290] P. L. Varghese and R. K. Hanson, Tunable infrared diode laser measurements of line strengths and collision widths of ^12^C^16^O at room temperature, J. Quant Spectrosc. Radiat. Transfer **24**, 479–489 (1980) I,FB.
[5.291] J. Mink, A. Ayoub, G. Kemeny, and F. Kling, The 1-0 band and rotational-vibrational constants of ^14^C-labeled carbon monoxide, J. Mol. Spectrosc. **86**, 258–261 (1981) W.
[5.292] J.-P. Bouanich, Nguyen-Van-Thanh, and H. Strapelias, Intensity, bandshapes and dipole correlation functions for the first overtone of compressed CO, J. Quant. Spectrosc. Radiat. Transfer **26**, 53–63 (1981) I,SB.
[5.293] J. N-P. Sun and P. R. Griffiths, Temperature dependence of the self-broadening coefficients for the fundamental band of carbon monoxide, Appl. Opt. **20**, 1691–1695 (1981) SB.
[5.294] G. Guelachvili, Differential Fourier spectroscopy with simultaneous interferograms: application to extensive accurate pressure-shift measurements, Appl. Opt. **20**, 2121–2132 (1981) PS.
[5.295] G. Chandraiah and G. R. Hebert, Absorption intensity measurements of the first overtone band of CO, Can. J. Phys. **59**, 1367–1372 (1981) I.
[5.296] J. J. BelBruno, J. Gelfand, W. Radigan, and K. Verges, Helium and self-broadening in the first and second overtone bands of ^12^C^16^0, J. Mol. Spectrosc. **94**, 336–342 (1982) SB,FB.
[5.297] H. S. Lowry and C. J. Fisher, Line parameter measurements and calculations of CO broadened by nitrogen at elevated temperatures, J. Quant. Spectrosc. Radiat. Transfer **27**, 585–591 (1982) I,FB.
[5.298] T. Nakazawa and M Tanaka, Measurements of intensities and self- and foreign-gas-broadencd half-widths of spectral lines in the CO fundamental band, J. Quant. Spectrosc. Radiat. Transfer **28**, 409–416 (1982) I,SB,FB.
[5.299] T. Nakazawa and M. Tanaka, Intensities, Half-widths and Shapes of Spectral Lines in the Fundamental Band of CO at Low Temperatures, J. Quant. Spectrosc. Radiat. Transfer **28**, 471–480 (1982) I,SB,FB.
[5.300] C. Chaekerian, G. Guelachvili, and R. H. Tipping, CO 1-0 band isotopic lines as intensity standards, J. Quant. Spectrosc. Radiat. Transfer **30**, 107–112 (1983) I,T.
[5.301] G. Guelachvili, D. de Villeneuve, R. Farrenq, W. Urban, and J. Verges, Dunham coefficients for seven isotopic species of CO, J. Mol. Spectrosc. **98**, 64–79 (1983) W.
[5.302] J.-P. Bouanich, Lineshifts in the first overtone band of CO self-perturbed and perturbed by N_2_ at 298, 193, and 133 K, Can. J. Phys. **61**, 919–921 (1983) PS,T.
[5.303] K. Kim, The integrated intensity of the carbon monoxide fundamental band, J. Quant. Spectrosc. Radiat. Transfer **30**, 413–416 (1983) I.
[5.304] C. R. Pollock, F. R. Petersen, D. A. Jennings, J. S. Wells, and A. G. Maki, Absolute frequency measurements of the 2-0 band of CO at 2.3 *μ*m; calibration standard frequencies from high resolution color center laser spectroscopy, J. Mol. Spectrosc. **99**, 357–368 (1983) F,PS.
[5.305] C. Chackerian and R. H. Tipping, Vibration-rotational and rotational intensities for CO isotopes, J. Mol. Spectrosc. **99**, 431–449 (1983) T.
[5.306] L. R. Brown and R. A. Toth, Comparison of the frequencies of NH_3_, CO_2_, H_2_O, N_2_O, CO, and CH_4_ as infrared calibration standards, J. Opt. Soc. Am. B **2**, 842–856 (1985) W.
[5.307] G. Guelachvili and K. Narahari Rao, Handbook of Infrared Standards, Academic Press, San Diego, 1986 W.
[5.308] J. M. Hartmann, M. Y. Perrin, J. Taine, and L. Rosenmann, Diode-laser measurements and calculations of CO 1-0 P(4) line broadening in the 294- to 765-K temperature range, J. Quant. Spectrosc. Radiat. Transfer **35**, 357–363 (1986) I,FB.
[5.309] I. G. Nolt, J. V. Radostitz, G. DiLonardo, K. M. Evenson, D. A. Jennings, K. R. Leopold, M. D. Vanek, L. R. Zink, A. Hinz, and K. V. Chance, Aceurate rotational constants of CO, HCl, and HF: spectral standards for the 0.3- to 6-THz (10- to 200-cm^−1^) region, J. Mol. Spectrosc. **125**, 274–287 (1987) F.
[5.310] J.-P. Bouanich, Vibration-rotation matrix elements for infrared transitions of diatomic molecules, J. Quant. Spectrosc. Radiat. Transfer **37**, 17–46 (1987) I.
[5.311] N. Semmoud-Monnanteuil and J. M. Colmont, Pressure broadening of millimeter lines of carbon monoxide, J. Mol. Spectrosc. **126**, 210–219 (1987) SB,FB.
[5.312] J.-P. Bouanich and G. Blanquet, Pressure broadening of CO and OCS spectral lines, J. Quant. Spectrosc. Radiat. Transfer **40**, 205–220 (1988) T,SB,FB.
[5.313] M. Schneider, K. M. Evenson, M. D. Vanek, D. A. Jennings, J. S. Wells, A. Stahn, and W. Urban, Heterodyne frequency measurements of ^12^C^16^O laser transitions, J. Mol. Spectrosc. **135**, 197–206 (1989) F.
[5.314] M. Schneider, J. S. Wells, and A. G. Maki, Heterodyne frequency measurements of ^12^C^16^O laser transitions near 2050 cm^−1^, J. Mol. Spectrosc. **139**, 432–438 (1990), and **141**, 351 (1990) F.
[5.315] L. R. Zink, P. De Natale, F. S. Pavone, M. Prevedelli, K. M. Evenson, and M. Inguscio, Rotational far infrared spectrum of ^13^CO, J. Mol. Spectrosc. **143**, 304–310 (1990) F,W.
[5.316] A. G. Maki, J. S. Wells, and D. A. Jennings, Heterodyne frequency measurements on CO and OCS beyond 2100 cm^−1^, J. Mol. Spectrosc. **144**, 224–229 (1990) F.
[5.317] A. LeFloch, Revised molecular constants for the ground state of CO, Mol. Phys. **72**, 133–144 (1991) T.
[5.318] D. Bailly, C. Rossetti, F. Thibault, and R. Le Doucen, ^12^C^16^O: Experimental determination of the linear coefficient of the Herman-Wallis factor, J. Mol. Spectrosc. **148**, 329–337 (1991) I.
[5.319] R. Farrenq, G. Guelachvili, A. J. Sauval, N. Grevesse, and C. B. Farmer, Improved Dunham coefficients for CO from infrared solar lines of high rotational excitation, J. Mol. Spectrosc. **149**, 375–390 (1991) W.
[5.320] T. D. Varberg and K. M. Evenson, Accurate far-infrared rotational frequencies of carbon monoxide, Astrophys. J. (in press) F.
[5.321] T. George, B. Wu, A. Dax, M. Schneider, and W. Urban, Saturation stabilization of the CO fundamental band laser, Appl. Phys. B (in press) F.

### 5.6 NO

[5.322] K. Narahari Rao, R. V. deVore, and E. K. Plyler, Wavelength calibrations in the far infrared (30 to 1000 microns), J. Res. NBS **67A**, 351–358 (1963) W.
[5.323] T. C. James, Intensity of the forbidden X^2^∏_3_/2-X^2^∏_1/2_ satellite bands in the infrared spectrum of nitric oxide, J. Chem. Phys. **40**, 762–771 (1964) I,T,SB.
[5.324] T. C. James and R. J. Thibault, Spin-orbit coupling constant of nitric oxide. Determination from fundamental and satellite band origins, J. Chem. Phys. **41**, 2806–2813 (1964) W.
[5.325] R. L. Brown and H. E. Radford, L-uncoupling effects on the electron-paramagnetic-resonance spectra of N^14^O^16^ and N^15^O^16^, Phys. Rev. **147**, 6–12 (1966) F.
[5.326] L. L. Abels and J. H. Shaw, Widths and strengths of vibration-rotation lines in the fundamental band of nitric oxide, J. Mol. Spectrosc. **20**, 11–28 (1966) I,SB.
[5.327] C. Alamiehel, Etude de la dispersion dans la bande fondamentale de vibration-rotation de NO, J. Phys. (Paris) **27**, 345–352 (1966) I.
[5.328] R. M. Neumann, High-precision radiofrequency spectrum of ^14^N^16^O, Astrophys. J. **161**, 779–784 (1970) F.
[5.329] J. M. Brown, A. R. H. Cole, and F. R. Honey, Magnetic dipole transitions in the far infra-red spectrum of nitric oxide, Mol. Phys. **23**, 287–295 (1972) I,W.
[5.330] W. T. King and B. Crawford, Jr., The integrated intensity of the nitric oxide fundamental band, J. Quant. Spectrosc. Radiat. Transfer **12**, 443–447 (1972) I.
[5.331] G. Chandraiah and C. W. Cho, A study of the fundamental and first overtone bands of NO in NO-rare gas mixtures at pressures up to 10,000 psi, J. Mol. Spectrosc. **47**, 134–147 (1973) I.
[5.332] K. Hakuta and H. Uehara, Laser magnetic resonance for the *v* = 1←0 transition of NO (2*∏*_3/2_) by CO laser, J. Mol. Spectrosc. **58**, 316–322 (1975) LS.
[5.333] A. Goldman and S. C. Schmidt, Infrared spectral line parameters and absorptance calculations of NO at atmospheric and elevated temperatures for the *Δv* = 1 bands region, J. Quant. Spectrosc. Radiat. Transfer **15**, 127–138 (1975) T.
[5.334] A. R. Hoy, J. W. C. Johns, and A. R. W. McKellar, Stark spectroscopy with the CO laser: dipole moments, hyperfine structure, and level crossing effects in the fundamental band of NO, Can. J. Phys. **53**, 2029–2039 (1975) LS.
[5.335] R. J. Butcher, R. B. Dennis, and S. D. Smith, The tunable spin-flip Raman laser II. Continuous wave molecular spectroscopy, Proc. Roy. Soc. (London) A **344**, 541–561 (1975) W.
[5.336] W. L. Meerts, A theoretical reinvestigation of the rotational and hyperfine lambda doubling spectra of diatomic molecules with a ^2^*Π* state: the spectrum of NO, Chem. Phys. **14**, 421–425 (1976) F,T
[5.337] R. E. Richten, NO line parameters measured by CO laser transmittance, Appl. Opt. **15**, 1686–1687 (1976) FB,SB.
[5.338] R. K. Hanson, J. P. Monat, and C. H. Kruger, Absorption of CO laser radiation by NO, J. Quant. Spectrosc. Radiat. Transfer **16**, 705–713 (1976) I,SB,F.
[5.339] G. D. T. Tejwani, B. M. Golden, and E. S. Yeung, Pressure-broadened linewidths of nitric oxide, J. Chem. Phys. **65**, 5110–5114 (1976) FB,SB,T.
[5.340] P. Rabache, Far infrared spectral lines and absorptance calculation of NO, J. Quant. Spectrosc. Radiat. Transfer **17**, 673–678 (1977) I,W.
[5.341] J. W. C. Johns, J. Reid, and D. W. Lepard, The vibration-rotation fundamental of NO, J. Mol. Spectrosc. **65**, 155–162 (1977) W.
[5.342] M. A. Guerra, A. Sanchez, and A. Javan, v = 2←1 absorption spectroscopy of vibrationally heated NO molecules using optical pumping in a wave guide, Phys. Rev. Lett. **38**, 482–484 (1977) F.
[5.343] R. M. Dale, J. W. C. Johns, A. R. W. McKellar, and M. Riggin, High-resolution laser magnetic resonance and infrared-radiofrequency double-resonance spectroscopy of NO and its isotopes near 5.4 *μ*m, J. Mol. Spectrosc. **67**, 440–458 (1977) LS.
[5.344] B. K. Garside, E. A. Ballik, M. Elsherbiny, and J. Shewchun, Resonance absorption measurements of NO with a line-tunable CO laser: spectroscopic data for pollution monitoring, Appl. Opt. **16**, 398–402 (1977) I,SB.
[5.345] A. Valentin, A. Henry, Ph. Cardinet, M. F. Le Moal, Da-Wun Chen, and K. Narahari Rao, Measurement and interpretation of the 1-0 band of ^14^N^16^O at 1900 cm^−1^, J. Mol. Spectrsoc. **70**, 9–17 (1978) W.
[5.346] A. Henry, M. F. Le Moal, Ph. Cardinet, and A. Valentin, Overtone bands of ^14^N^16^O and determination of molecular constants, J. Mol. Spectrosc. **70**, 18–26 (1978) W.
[5.347] K. Kunimori, H. Horiguchi, and S. Tsuchiya, Intensity and line-width measurements of the NO fundamental by infrared molecular absorption spectrometry, J. Quant. Spectrosc. Radiat. Transfer **19**, 127–133 (1978) I,FB.
[5.348] L. D. G. Young, A. T. Young, S. A. Clough, and F. X. Kneizys, Calculation of spectroscopic data for the v = 0 and *v* = 1 states of nitric oxide, J. Quant. Spectrosc. Radiat. Transfer **20**, 317–325 (1978) I,W.
[5.349] R. E. Richton, Pressure-broadening linewidths of the R(9.5)_3/2_ NO transitions, Appl. Opt. **17**, 1606–1609 (1978) SB.
[5.350] C. Amiot, R. Bacis, and G. Guelachvili, Infrared study of the X^2^*Π* v = 0,1,2 levels of ^14^N^16^O. Preliminary results on the v = 0,1 levels of ^14^N^17^O, ^14^N^18^O, and ^15^N^16^O, Can. J. Phys. **56**, 251–265 (1978) W.
[5.351] L. A. Farrow and R. E. Richten, Extinction coefficient of the R_1/2_(25/2) NO transition at the 8-7 P(11) CO laser line, Appl. Opt. **18**, 597–599 (1979) LSB.
[5.352] A. S. Pine and K. W. Nill, Molecular-beam tunable-diode-laser sub-Doppler spectroscopy of *Λ*-doubling in nitric oxide, J. Mol. Spectrosc. **74**, 43–51 (1979) W.
[5.353] A. S. Pine, J. W. C. Johns, and A. G. Robiette, id-doubling in the *v* = 2←0 overtone band in the infrared spectrum of NO, J. Mol. Spectrosc. **74**, 52–69 (1979) W.
[5.354] L. D. G. Young and A. T. Young, Calculation of spectroscopic data for the *v* = 2 and *v* = 3 states of nitric oxide, J. Quant. Spectrosc. Radiat. Transfer **21**, 227–231 (1979) W,I.
[5.355] C. Amiot and G. Guelachvili, Infrared study of the ^15^N isotopic species of nitric oxide near 5.4 *μ*m, J. Mol. Spectrosc. **76**, 86–103 (1979) W.
[5.356] C. Amiot and J. Verges, The ^14^N^16^O ground state up to *v* = 15 by emission Fourier transform spectroscopy of the *Δv* = 2 sequence, J. Mol. Spectrosc. **81**, 424–444 (1980) W.
[5.357] W. L. Meerts and L. Vcscth, The Zeeman spectrum of the NO molecule, J. Mol. Spectrosc. **82**, 202–213 (1980) F.
[5.358] J. L. Teffo, A. Henry, Ph. Cardinet, and A. Valentin, Determination of molecular constants of nitric oxide from (1-0), (2-0), (3-0) bands of the ^15^N^16^O and ^15^N^18^O isotopic species, J. Mol. Spectrosc. **82**, 348–363 (1980) W.
[5.359] J.-Y. Mandin, C. Amiot, and G. Guelachvili, Intensity and sclf-broadcning coefficient measurements from Fourier transform spectra: application to the nitric oxide fundamental band, Ann. Phys. (Paris) **5**, 91–111 (1980) I,SB.
[5.360] R. Freedman and R. W. Nicholls, Molecular constants for the *v*″ = 0,1 (A^2^Σ^+^) levels of the NO molecule and its isotopes, J. Mol. Spectrosc. **83**, 223–227 (1980) W.
[5.361] W. Rohrbeck, R. Winter, W. Herrmann, J. Wildt, and W. Urban, Pressure broadening coefficients for nitric oxide, measured with a spin-flip Raman-laser spectrometer, Mol. Phys. **39**, 673–681 (1980) FB,SB.
[5.362] F. C. van den Heuvel, W. L. Meerts, and A. Dymanus, High-resolution tunable spectroscopy of rotational transitions of NO near 30 cm^−1^, J. Mol. Spectrosc. **84**, 162–169 (1980) F.
[5.363] R. S. Lowe, A. R. W. McKellar, P. Veillette, and W. L. Meerts, Hyperfine and *Λ*-doubling parameters for the *v* = 1 state of NO from infrared-radiofrequency double resonance, J. Mol. Spectrosc. **88**, 372–377 (1981) F,W.
[5.364] J. A. Sell, Infrared diode laser spectroscopy of nitric oxide, J. Quant. Spectrosc. Radiat. Transfer **25**, 19–24 (1981) I,FB.
[5.365] J. R. Gillis and A. Goldman, Nitric oxide IR line parameters for the upper atmosphere, Appl. Opt. **21**, 1161–1163 (1982) I.
[5.366] C. Amiot, The infrared emission spectrum of NO: analysis of the *Δv* = 3 sequence up to *v* = 22, J. Mol. Spectrosc. **94**, 150–172 (1982) W.
[5.367] P. K. Falcone, R. K. Hanson, and C. H. Kruger, Tunable diode laser measurements of the band strength and collision halfwidths of nitric oxide, J. Quant. Spectrosc. Radiat. Transfer **29**, 205–221 (1983) I,FB.
[5.368] R. F. Holland, M. C. Vasquez, W. H. Beattie, and R. S. McDowell, Absorptivity of nitric oxide in the fundamental vibrational band, J. Quant. Spectrosc. Radiat. Transfer **29**, 435–438 (1983) I.
[5.369] W. Lempert, G. J. Rosasco, and W. S. Hurst, Rotational collisional narrowing in the NO fundamental *Q* branch studied with cw stimulated Raman spectroscopy, J. Chem. Phys. **81**, 4241–4245 (1984) I,SB.
[5.370] A. S. Pine, A. G. Maki, and N.-Y. Chou, Pressure broadening, Iineshapes, and intensity measurements in the 2←0 band of NO, J. Mol. Spectrosc. **114**, 132–147 (1985) I,SB,FB,PS.
[5.371] A. Hinz, J. S. Wells, and A. G. Maki, Heterodyne frequency measurements on the nitric oxide fundamental band, J. Mol. Spectrosc. **119**, 120–125 (1986) F.
[5.372] W. J. Phillips and H. C. Walker, Nitrogen-broadened linewidths and strengths of nitric oxide utilizing tunable diode laser spectroscopy, J. Chem. Phys. **85**, 3213–3216 (1986) I,FB.
[5.373] T. G. Neiss, R. W. Lovejoy, and C. Chackerian, Jr. Pressure broadening coefficients of ^14^N^16^O-N_2_ gas mixtures, J. Mol. Spectrosc. **124**, 229–235 (1987) I,FB.
[5.374] J. Ballard, W. B. Johnston, B. J. Kerridge, and J. J. Remedios, Experimental spectral line parameters in the 1-0 band of Nitric oxide, J. Mol. Spectrosc. **127**, 70–82 (1988) I,SB,FB.

### 5.7 CS_2_

[5.375] D. Z. Robinson, The experimental determination of the intensities of infrared absorption bands. IV. Measurements of the stretching vibrations of OCS and CS_2_, J. Chem. Phys. **19**, 881–886 (1951) I.
[5.376] H. J. Callomon, D. C. McKcan, and H. W. Thompson, Intensities of vibration bands. IV. Carbonyl sulphide and acetylene, Proc. Roy. Soc. (London) A **208**, 341–351 (1951) I.
[5.377] R. Kiyama and K. Ozawa, Rev. Phys. Chem. Japan **25**, 38 (1955) I.
[5.378] D. C. McKean, H. J. Callomon, and H. W. Thompson, Intensities of vibration bands of carbonyl sulfide and carbon disulfide, J. Chem. Phys. **20**, 520 (1951) I.
[5.379] W. B. Person and L. C. Hall, Absolute infrared intensities of CS_2_ fundamentals in gas and liquid phases. An interpretation of the bond moments of CO_2_ and CS_2_, Spectrochimica Acta **20**, 771–779 (1964) I.
[5.380] H. Yamada and W. B. Person, Absolute infrared intensities of some linear triatomic molecules in the gas phase, J. Chem. Phys. **45**, 1861–1865 (1966) I.
[5.381] D. F. Smith, T. Chao, J. Lin, and J. Ovcrcnd, High resolution of ^12^CS_2_ and ^13^CS_2_ and the *v*_3_ – *v*_1_, *v*_3_ *–* 2*v*_2_ and *v*_3_ + 4*v*_2_ bands, Spectrochim. Acta A **27**, 1979–1987 (1970) W.
[5.382] K. Jolma and J. Kauppinen, High-resolution infrared spectrum of CS_2_ in the region of the bending fundamental *v*_2_, J. Mol. Spectrosc. **82**, 214–219 (1980) W.
[5.383] G. Blanquet, J. Walrand, and C. P. Courtoy, La bande *v*_3_ du disulfure de carbone ^12^C^34^S_2_ et ^13^C^34^S_2_, Ann. Soc. Scient. Bruxelles **94**, 129–139 (1980) W.
[5.384] E. Baeten, G. Blanquet, J. Walrand, and C. P. Courtoy, Spectres infrarouges a haute resolution du disulfure de carbone ^12^C^32^S_2_ et ^12^C^34^S_2_ la bande *v*_3_ – *v*_1_, Ann. Soc. Scient. Bruxelles **97**, 229–241 (1984) W.
[5.385] E. Baeten, G. Blanquet, J. Walrand, and C. P. Courtoy, Tunable diode laser spectra of the *v*_3_ – *v*_1_ region of CS_2_, Can. J. Phys. **62**, 1286–1292 (1984) W.
[5.386] J. Lindenmayer and H. Jones, Diode laser spectroscopy of the *v*_3_ band region of four isotopic forms of CS_2_, J. Mol. Spectrosc. **110**, 65–73 (1985) W.
[5.387] G. Blanquet, E. Baeten, I. Cauuct, J. Walrand, and C. P. Courtoy, Diode-laser Measurements of carbon disulfide and general rovibrational analysis, J. Mol. Spectrosc. **112**, 55–70 (1985) W.
[5.388] M. Dang-Nhu, G. Blanquet, J. Walrand, and C. P. Courtoy, Spectral intensities in the *v*_3_ – *v*_1_ band of CS_2_, Mol. Phys. **58**, 995–1000 (1986) I.
[5.389] F. Winther, U. Heyne, and A. Guarnieri, The infrared spectrum of CS_2_ in the *v*_3_ band region, Z. Naturforsch. **43a**, 215–218 (1988) W.
[5.390] J. S. Wells, M. Schneider, and A. G. Maki, Calibration tables covering the 1460-1550-cm^−1^ region from heterodyne frequency measurements on the *v*_3_ bands of ^12^CS_2_ and ^13^CS_2_, J. Mol. Spectrosc. **132**, 422–428 (1988) F.

## 6. Atlas and Wavenumber Tables

### 6.1 Scope of the Atlas

The range of wavenumber coverage of the molecular bands is indicated below for the five molecules selected for use.

CO Atlas–1948 to 2275 cm^−1^ and 4071 to 4352 cm^−1^
OCS Atlas–486 to 567 cm^−1^
OCS Atlas–812 to 890 cm^−1^
OCS Atlas–1000 to 1095 cm^−1^
OCS Atlas–1650 to 1739 cm^−1^
OCS Atlas–1832 to 1934 cm^−1^
OCS Atlas–1970 to 2141 cm^−1^
OCS Atlas–2510 to 2600 cm^−1^
OCS Atlas−2693 to 2763 cm^−1^
OCS Atlas–2862 to 2970 cm^−1^
OCS Atlas–3065 to 3120 cm^−1^
N_2_O Atlas–523 to 659 cm^−1^
N_2_O Atlas–880 to 1087 cm^−1^
N_2_O Atlas–1105 to 1345 cm^−1^
N_2_O Atlas–1820 to 1925 cm^−1^
N_2_O Atlas–2140 to 2269 cm^−1^
N_2_O Atlas–2400 to 2607 cm^−1^
N_2_O Atlas–2725 to 2842 cm^−1^
CS_2_ Atlas–1460 to 1551 cm^−1^
NO Atlas–1741 to 1940 cm^−1^

#### 6.1.1 Description

Throughout the wavenumber tables the lines that are suitable for use as wavenumber standards are indicated by an asterisk (*) following the wavenumber and its uncertainty. In assigning the asterisks no consideration was given to problems related to overlapping with other transitions. The tables list nearby lines that may cause problems with overlapping. The user must exercise judgment in determining if such overlapping will impair the accuracy of the measurement. The asterisk only certifies the accuracy of the line position in the hypothetical absence of any other nearby lines. Obviously, the resolution of the instrumentation being used will determine if a nearby line might invalidate the accuracy of a calibration line.

The uncertainties of the wavenumbers are given in parentheses after the wavenumbers in those cases where there is reason to believe that a good estimate of the uncertainty can be made. Even so, the uncertainty in the lines not designated as calibration lines should be taken with some degree of skepticism.

The uncertainties given in the tables are twice the estimated standard error as calculated from the variance-covariance matrix given by the least-squares fit that determined the constants used to calculate the wavenumbers. The uncertainties refer to the accuracy of each individual transition. In general, the wavenumber separation of two nearby lines for the same vibrational transition of the same molecular species will be given more accurately than the uncertainty given in parentheses might lead one to believe. That is because the relative differences between the rotational energy levels are usually known more accurately than the differences in the vibrational energy levels.

On the other hand, the separation of two lines that are due to absorption from two different isotopic species is probably known no more accurately than the uncertainty (given in parentheses) would lead us to believe.

The wavenumbers given in the tables are calculated wavenumbers because they are more reliable than individual line measurements and the uncertainties in the calculated wavenumbers can be accurately estimated.

The wavenumber tables also contain a column for the intensity estimated for each transition at a temperature of 296 K. The format for the intensity values is the standard computer format consisting of a decimal value followed by the exponent (the power of ten multiplying the decimal value). The intensities given in the wavenumber tables are represented by *S* in Eq. (3.10) and are integrated line intensities rather than peak intensities.

In the sections giving discussions and equations on intensity calculations and on pressure broadening (Sec. 3), equations are given for estimating the appearance of the spectrum for different experimental conditions. In particular, Eq. (3.37) can be used to estimate the percent transmission at the center of a line under different conditions of pressure and pathlength. As an aid in calculating intensities at different temperatures the tables for OCS and N_2_O also contain a column giving the separation (in cm^−1^) of the lower state energy level from the ground state. The units given in the tables can be converted to the more common units of cm^−2^ atm^−1^ at 296 K by multiplying by 2.479 × 10^19^. To convert to intensities at some other temperature one should refer to Eq. (3.10). Smith et al. [5.2] give a table for converting to other units.

The intensity values given in this work are only given as an aid in estimating the appearance of the spectrum, they should not be treated as well determined values. The intensities given for weak lines and especially for the rarer isotopes may be in error by 50 percent or more.

The spectral illustrations were actually calculated spectra rather than reproductions of real measurements. This gave us more flexibility in choosing effective pressures and pathlengths that seemed most appropriate to illustrate even the weak lines. As with any digitized spectrum, regardless of whether it is calculated or measured, the peak intensity of sharp lines may show some irregularity depending on whether the true peak falls on a digitized point or slightly misses it. The spectra were plotted with a digitizing interval of about 0.0005 to 0.001 cm^−1^. Close doublets that should have the same intensity may show slight intensity differences because of this digitizing effect.

Comparison with real spectra measured in our own laboratory or illustrated in published works (such as Refs. [5.101, 5.102, 5.307]) showed that the spectra given in this work are adequate for identifying the calibration lines. Some weak transitions may be absent from the calculated spectrum even though they might be found in a real spectrum of comparable pressure and pathlength. Certainly, absorption due to common impurities such as H_2_O or CO_2_ will not be found in these spectra.

The spectra used in the illustrations for CO were calculated for infinite resolution but the lines were given widths dictated by the Doppler width of the line convolved with the pressure broadened width. For the CO spectra the shape of the weak lines is dominated by the Doppler width which is 0.0047 cm^−1^ (FWHH) at 2000 cm^−1^ and 0.0093 cm^−1^ at 4000 cm^−1^. For the conditions chosen for the atlas illustrations, the strong CO lines are much broader than the weak ones and show pronounced shoulders due to the effect of even a very small pressure broadening.

The figures used for illustrating the OCS and N_2_O spectra were calculated for spectrometer resolutions on the order of 0.003 cm^−1^. Again the expected Doppler and pressure-broadened lineshapes were used in calculating the spectra. The atlas for OCS and N_2_O is divided into sections according to the vibrational transitions involved. At the beginning of each section the parameters (slit width, dipole derivative, Herman-Wallis constants) used in calculating the spectra are given as well as a key to the abbreviations used for the vibrational transitions in the atlas.

In calculating the pressure-broadened width, a single value for the pressure broadening was used for all lines in a spectrum. It is well known that such an assumption is incorrect and therein lies one reason for the illustration to depart slightly from a true spectrum.

The spectra overlap slightly in order to show the relationship of the lines near the ends of each panel. At the top of each spectrum fiducial marks indicate which lines are given in the accompanying wavenumber tables. Every fifth line is indicated with a darker and longer mark. In the tables, every fifth line is set apart by a following blank line. Even though the panels overlap, each line is identified by a fiducial mark only once. The next section gives a key to symbols in the atlas, followed by a sample spectral map with facing table.

### 6.2 Key to Symbols and Sample Spectral Map with Facing Table

**Table:** 

Band	Isotopomer	Vibrational transition
A	^16^O^12^C^32^S	02^0^0–00^0^0
X		02^2e^0–00^0^0
B		03^1^0-01^1e^0
C		03^1^0–01^1f^0
D		02^0^1–00^0^1
E		04^2^0–02^2e^0
F		04^2^0–02^2f^0
G		04^0^0–02^0^0
H		03^1^1–01^1e^1
I		03^1^1–01^1f^1
J		05^3^0–03^3e^0
K		05^3^0–03^3f^0
L		05^1^0–03^1e^0
M		05^1^0–03^1f^0
P	^16^O^12^C^34^S	02^0^0–00^0^0
Q		03^1^0–01^1e^0
R		03^1^0–01^1f^0
T	^16^O^13^C^32^S	02^0^0–00^0^0

If the *e* or *f* designation is not specified (for *l* ≠ 0), then the transition is to either level, depending on the selection rules and the change in the rotational quantum number, *J*. Spectra are given for a slitwidth of 0.002 cm^−1^ and a temperature of 296 K. For the A, X, D, P, and T bands a transition moment of 0.0333 D was used and no Herman-Wallis constant was included in the intensity calculation. For the other bands a transition moment of 0.032 D was used with a Herman-Wallis constant of *C*_1_ = 0.0019.

**Figure:**
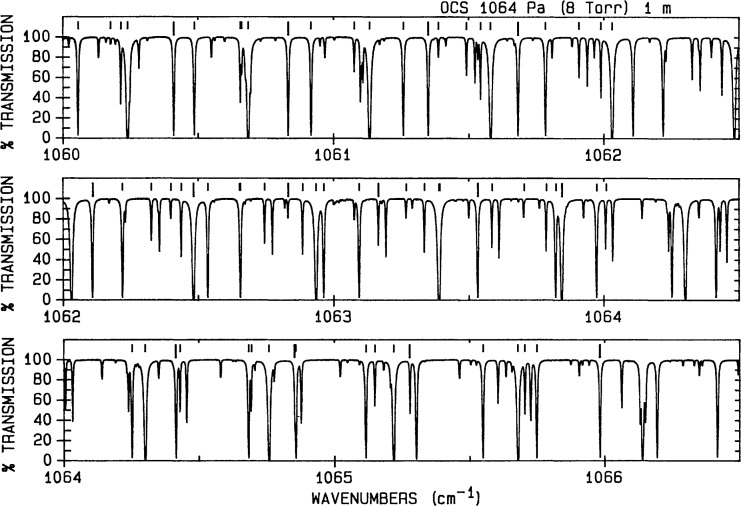


**Table:** 

LINE	WAVENUMBER(unc)	LOWER STATE	INTENSITY	ASSIGNMENT	
#	(cm^−1^)	(cm^−1^)	(cm/molecule)	ROTATION	BAND
1	1060.056 36(6)*	575.72	0.739E-21	R(16)	C
2	1060.176 77(10)*	1574.38	0.104E-22	R(59)	D
3	1060.214 07(24)	249.29	0.173E-21	R(35)	P
4	1060.239 490(13)*	188.62	0.464E-20	R(30)	A
5	1060.410 14(6)*	582.57	0.759E-21	R(17)	B
6	1060.48640(6)*	582.63	0.758E-21	R(17)	C
7	1060.656 05(24)	263.53	0.166E-21	R(36)	P
8	1060.661 66(9)*	1055.59	0.583E-22	R(6)	G
9	1060.685 510(13)*	201.19	0.451E-20	R(31)	A
10	1060.833 43(6)*	589.88	0.775E-21	R(18)	B
11	1060.917 75(6)*	589.95	0.775E-21	R(18)	C
12	1061.076 43(9)*	1058.44	0.659E-22	R(7)	G
13	1061.132 833(13)*	214.17	0.437E-20	R(32)	A
14	1061.257 62(6)*	597.60	0.788E-21	R(19)	B
15	1061.350 39(6)*	597.68	0.787E-21	R(19)	C
16	1061.388 43(16)	648.43	0.349E-22	R(25)	Q
17	1061.492 19(9)*	1061.69	0.731E-22	R(8)	G
18	1061.543 93(24)	293.19	0.151E-21	R(38)	P
19	1061.581 464(14)*	227.55	0.422E-20	R(33)	A
20	1061.682 71(6)*	605.72	0.797E-21	R(20)	B
21	1061.784 33(6)*	605.81	0.797E-21	R(20)	C
22	1061.908 92(9)*	1065.36	0.800E-22	R(9)	G
23	1061.989 85(24)	308.62	0.144E-21	R(39)	P
24	1062.031 406(14)*	241.34	0.406E-20	R(34)	A
25	1062.108 69(6)*	614.25	0.803E-21	R(21)	B
26	1062.219 56(6)*	614.35	0.803E-21	R(21)	C
27	1062.326 65(9)*	1069.42	0.864E-22	R(10)	G
28	1062.398 22(16)	669.58	0.341E-22	R(27)	R
29	1062.437 09(24)	324.44	0.137E-21	R(40)	P
30	1062.482 665(14)*	255.53	0.390E-20	R(35)	A
31	1062.535 58(6)*	623.18	0.806E-21	R(22)	B
32	1062.652 89(15)	680.52	0.336E-22	R(28)	Q
33	1062.656 10(6)*	623.29	0.805E-21	R(22)	C
34	1062.745 37(9)*	1073.90	0.925E-22	R(11)	G
35	1062.832 18(16)	680.68	0.336E-22	R(28)	R
36	1062.885 66(24)	340.66	0.130E-21	R(41)	P
37	1062.935 247(14)*	270.13	0.373E-20	R(36)	A
38	1062.963 36(6)*	632.52	0.805E-21	R(23)	B
39	1063.093 94(6)*	632.64	0.805E-21	R(23)	C
40	1063.165 09(9)*	1078.78	0.980E-22	R(12)	G
41	1063.267 42(15)	692.18	0.329E-22	R(29)	R
42	1063.335 56(24)	357.27	0.122E-21	R(42)	P
43	1063.389 156(14)*	285.13	0.356E-20	R(37)	A
44	1063.392 04(6)*	642.27	0.802E-21	R(24)	B
45	1063.533 07(6)*	642.40	0.801E-21	R(24)	C
46	1063.585 80(9)*	1084.07	0.103E-21	R(13)	G
47	1063.703 96(15)	704.08	0.322E-22	R(30)	R
48	1063.786 81(24)	374.27	0.115E-21	R(43)	P
49	1063.821 62(6)*	652.42	0.796E-21	R(25)	B
50	1063.844 398(14)*	300.54	0.339E-20	R(38)	A
51	1063.973 50(6)*	652.56	0.795E-21	R(25)	C
52	1064.007 52(9)*	1089.77	0.108E-21	R(14)	G
53	1064.252 10(6)*	662.98	0.787E-21	R(26)	B
54	1064.300 978(14)*	316.35	0.322E-20	R(39)	A
55	1064.415 24(6)*	663.13	0.786E-21	R(26)	C
56	1064.430 25(9)*	1095.88	0.112E-21	R(15)	G
57	1064.683 48(6)*	673.94	0.775E-21	R(27)	B
58	1064.693 35(24)	409.47	0.102E-21	R(45)	P
59	1064.758 903(14)*	332.57	0.305E-20	R(40)	A
60	1064.854 00(9)*	1102.39	0.115E-21	R(16)	G
61	1064.858 28(6)*	674.10	0.775E-21	R(27)	C
62	1065.115 76(6)*	685.31	0.761E-21	R(28)	B
63	1065.148 66(24)	427.66	0.949E-22	R(46)	P
64	1065.218 178(14)*	349.19	0.289E-20	R(41)	A
65	1065.278 77(9)*	1109.30	0.118E-21	R(17)	G
66	1065.548 94(6)*	697.09	0.746E-21	R(29)	B
67	1065.678 810(14)*	366.22	0.272E-20	R(42)	A
68	1065.704 57(9)*	1116.63	0.121E-21	R(18)	G
69	1065.748 25(6)*	697.27	0.745E-21	R(29)	C
70	1065.983 03(6)*	709.27	0.728E-21	R(30)	B

## Figures and Tables

**Table 1:** Frequencies (MHz) for the 626 carbon dioxide laser

Rot. trans.	Frequency (MHz)	Rot. trans.	Frequency (MHz)
P(50)	27 413 600.4112	P(50)	30 480 527.0432
P(48)	27 478 430.1483	P(48)	30 545 874.3287
P(46)	27 542 482.6299	P(46)	30 610 516.1385
P(44)	27 605 762.5803	P(44)	30 674 445.7649
P(42)	27 668 274.4491	P(42)	30 737 656.7009
P(40)	27 730 022.4165	P(40)	30 800 142.6462
P(38)	27 791 010.3989	P(38)	30 861 897.5131
P(36)	27 851 242.0547	P(36)	30 922 915.4319
P(34)	27 910 720.7882	P(34)	30 983 190.7566
P(32)	27 969 449.7554	P(32)	31 042 718.0701
P(30)	28 027 431.8676	P(30)	31 101 492.1893
P(28)	28 084 669.7958	P(28)	31 159 508.1695
P(26)	28 141 165.9746	P(26)	31 216 761.3094
P(24)	28 196 922.6060	P(24)	31 273 247.1550
P(22)	28 251 941.6621	P(22)	31 328 961.5037
P(20)	28 306 224.8891	P(20)	31 383 900.4083
P(18)	28 359 773.8096	P(18)	31 438 060.1801
P(16)	28 412 589.7252	P(16)	31 491 437.3923
P(14)	28 464 673.7190	P(14)	31 544 028.8828
P(12)	28 516 026.6578	P(12)	31 595 831.7569
P(10)	28 566 649.1936	P(10)	31 646 843.3897
P(8)	28 616 541.7658	P(8)	31 697 061.4282
P(6)	28 665 704.6019	P(6)	31 746 483.7926
P(4)	28 714 137.7193	P(4)	31 795 108.6785
P(2)	28 761 840.9258	P(2)	31 842 934.5572
R(0)	28 832 026.2179	R(0)	31 913 172.5750
R(2)	28 877 902.4362	R(2)	31 958 996.0676
R(4)	28 923 046.4283	R(4)	32 004 017.3874
R(6)	28 967 457.0638	R(6)	32 048 236.2545
R(8)	29 011 133.0037	R(8)	32 091 652.6661
R(10)	29 054 072.6995	R(10)	32 134 266.8957
R(12)	29 096 274.3924	R(12)	32 176 079.4916
R(14)	29 137 736.1122	R(14)	32 217 091.2759
R(16)	29 178 455.6756	R(16)	32 257 303.3427
R(18)	29 218 430.6853	R(18)	32 296 717.0558
R(20)	29 257 658.5273	R(20)	32 335 334.0465
R(22)	29 296 136.3697	R(22)	32 373 156.2114
R(24)	29 333 861.1596	R(24)	32 410 185.7086
R(26)	29 370 829.6209	R(26)	32 446 424.9556
R(28)	29 407 038.2514	R(28)	32 481 876.6251
R(30)	29 442 483.3197	R(30)	32 516 543.6414
R(32)	29 477 160.8619	R(32)	32 550 429.1766
R(34)	29 511 066.6779	R(34)	32 583 536.6463
R(36)	29 544 196.3277	R(36)	32 615 869.7049
R(38)	29 576 545.1272	R(38)	32 647 432.2414
R(40)	29 608 108.1437	R(40)	32 678 228.3735
R(42)	29 638 880.1914	R(42)	32 708 262.4432
R(44)	29 668 855.8266	R(44)	32 737 539.0112
R(46)	29 698 029.3421	R(46)	32 766 062.8508
R(48)	29 726 394.7621	R(48)	32 793 838.9426
R(50)	29 753 945.8362	R(50)	32 820 872.4682

**Table 2:** Frequencies (MHz) for the 636 carbon dioxide laser

Rot. trans.	Frequency (MHz)	Rot. trans.	Frequency (MHz)
P(50)	26 035 339.9907	P(50)	29 076 007.9206
P(48)	26 096 450.6641	P(48)	29 143 127.3150
P(46)	26 156 946.4184	P(46)	29 209 472.6217
P(44)	26 216 830.6112	P(44)	29 275 036.8793
P(42)	26 276 106.3711	P(42)	29 339 813.3299
P(40)	26 334 776.6055	P(40)	29 403 795.4269
P(38)	26 392 844.0080	P(38)	29 466 976.8408
P(36)	26 450 311.0648	P(36)	29 529 351.4663
P(34)	26 507 180.0614	P(34)	29 590 913.4283
P(32)	26 563 453.0887	P(32)	29 651 657.0881
P(30)	26 619 132.0481	P(30)	29 711 577.0488
P(28)	26 674 218.6570	P(28)	29 770 668.1612
P(26)	26 728 714.4536	P(26)	29 828 925.5288
P(24)	26 782 620.8011	P(24)	29 886 344.5126
P(22)	26 835 938.8920	P(22)	29 942 920.7360
P(20)	26 888 669.7515	P(20)	29 998 650.0890
P(18)	26 940 814.2413	P(18)	30 053 528.7321
P(16)	26 992 373.0624	P(16)	30 107 553.1003
P(14)	27 043 346.7579	P(14)	30 160 719.9062
P(12)	27 093 735.7156	P(12)	30 213 026.1432
P(10)	27 143 540.1699	P(10)	30 264 469.0880
P(8)	27 192 760.2040	P(8)	30 315 046.3033
P(6)	27 241 395.7512	P(6)	30 364 755.6398
P(4)	27 289 446.5964	P(4)	30 413 595.2373
P(2)	27 336 912.3769	P(2)	30 461 563.5271
R(0)	27 407 012.8973	R(0)	30 531 879.5457
R(2)	27 453 013.4681	R(2)	30 577 664.6182
R(4)	27 498 426.5523	R(4)	30 622 575.1932
R(6)	27 543 251.1292	R(6)	30 666 611.0177
R(8)	27 587 486.0315	R(8)	30 709 772.1308
R(10)	27 631 129.9443	R(10)	30 752 058.8623
R(12)	27 674 181.4045	R(12)	30 793 471.8321
R(14)	27 716 638.7993	R(14)	30 834 011.9476
R(16)	27 758 500.3646	R(16)	30 873 680.4025
R(18)	27 799 764.1833	R(18)	30 912 478.6740
R(20)	27 840 428.1829	R(20)	30 950 408.5203
R(22)	27 880 490.1333	R(22)	30 987 471.9773
R(24)	27 919 947.6441	R(24)	31 023 671.3556
R(26)	27 958 798.1612	R(26)	31 059 009.2364
R(28)	27 997 038.9638	R(28)	31 093 488.4680
R(30)	28 034 667.1602	R(30)	31 127 112.1610
R(32)	28 071 679.6844	R(32)	31 159 883.6838
R(34)	28 108 073.2910	R(34)	31 191 806.6579
R(36)	28 143 844.5509	R(36)	31 222 884.9524
R(38)	28 178 989.8459	R(38)	31 253 122.6787
R(40)	28 213 505.3631	R(40)	31 282 524.1845
R(42)	28 247 387.0891	R(42)	31 311 094.0480
R(44)	28 280 630.8034	R(44)	31 338 837.0715
R(46)	28 313 232.0718	R(46)	31 365 758.2751
R(48)	28 345 186.2387	R(48)	31 391 862.8896
R(50)	28 376 488.4197	R(50)	31 417 156.3497

**Table 3:** Frequencies (MHz) for the 828 carbon dioxide laser

Rot. trans.	Frequency (MHz)	Rot. trans.	Frequency (MHz)
P(50)	27 702 788.3340	P(50)	31 275 461.0484
P(48)	27 763 916.5271	P(48)	31 330 648.1933
P(46)	27 824 219.8818	P(46)	31 385 304.7512
P(44)	27 883 701.5085	P(44)	31 439 427.2029
P(42)	27 942 364.3439	P(42)	31 493 012.1188
P(40)	28 000 211.1532	P(40)	31 546 056.1633
P(38)	28 057 244.5316	P(38)	31 598 556.0988
P(36)	28 113 466.9070	P(36)	31 650 508.7885
P(34)	28 168 880.5416	P(34)	31 701 911.2012
P(32)	28 223 487.5337	P(32)	31 752 760.4139
P(30)	28 277 289.8196	P(30)	31 803 053.6153
P(28)	28 330 289.1753	P(28)	31 852 788.1092
P(26)	28 382 487.2180	P(26)	31 901 961.3173
P(24)	28 433 885.4075	P(24)	31 950 570.7820
P(22)	28 484 485.0477	P(22)	31 998 614.1692
P(20)	28 534 287.2879	P(20)	32 046 089.2707
P(18)	28 583 293.1240	P(18)	32 092 994.0069
P(16)	28 631 503.3995	P(16)	32 139 326.4282
P(14)	28 678 918.8066	P(14)	32 185 084.7176
P(12)	28 725 539.8870	P(12)	32 230 267.1924
P(10)	28 771 367.0327	P(10)	32 274 872.3053
P(8)	28 816 400.4870	P(8)	32 318 898.6464
P(6)	28 860 640.3445	P(6)	32 362 344.9440
P(4)	28 904 086.5522	P(4)	32 405 210.0657
P(2)	28 946 738.9095	P(2)	32 447 493.0190
R(0)	29 009 228.1753	R(0)	32 509 824.0588
R(2)	29 049 894.0639	R(2)	32 550 648.1734
R(4)	29 089 764.2422	R(4)	32 590 887.7557
R(6)	29 128 837.8481	R(6)	32 630 542.4476
R(8)	29 167 113.8723	R(8)	32 669 612.0318
R(10)	29 204 591.1583	R(10)	32 708 096.4309
R(12)	29 241 268.4016	R(12)	32 745 995.7070
R(14)	29 277 144.1494	R(14)	32 783 310.0604
R(16)	29 312 216.8002	R(16)	32 820 039.8289
R(18)	29 346 484.6028	R(18)	32 856 185.4857
R(20)	29 379 945.6557	R(20)	32 891 747.6385
R(22)	29 412 597.9061	R(22)	32 926 727.0276
R(24)	29 444 439.1492	R(24)	32 961 124.5238
R(26)	29 475 467.0268	R(26)	32 994 941.1261
R(28)	29 505 679.0261	R(28)	33 028 177.9601
R(30)	29 535 072.4788	R(30)	33 060 836.2746
R(32)	29 563 644.5594	R(32)	33 092 917.4396
R(34)	29 591 392.2837	R(34)	33 124 422.9433
R(36)	29 618 312.5075	R(36)	33 155 354.3890
R(38)	29 644 401.9248	R(38)	33 185 713.4920
R(40)	29 669 657.0662	R(40)	33 215 502.0763
R(42)	29 694 074.2966	R(42)	33 244 722.0715
R(44)	29 717 649.8141	R(44)	33 273 375.5085
R(46)	29 740 379.6471	R(46)	33 301 464.5166
R(48)	29 762 259.6531	R(48)	33 328 991.3192
R(50)	29 783 285.5157	R(50)	33 355 958.2301

**Table 4:** Frequencies and wavenumbers for the 01^1^1 –[11 ^1^0.03^1^0]_1_, band of ^13^CO_2_

Rot. trans.	Frequency (MHz)	Rot. trans.	Frequency (MHz)^a^
P(50)	25 110 914.276(1909)	R(1)	26 522 359.352(307)
P(49)	25 163 452.505(1658)	R(2)	26 545 384.349(356)
P(48)	25 173 184.219(1500)	R(3)	26 568 137.000(372)
P(47)	25 223 707.197(1300)	R(4)	26 590 844.300(435)
P(46)	25 234 801.135(1157)	R(5)	26 613 344.206(446)
P(45)	25 283 388.000(1001)	R(6)	26 635 672.514(520)
P(44)	25 295 767.670(874)	R(7)	26 657 980.003(528)
P(43)	25 342 496.069(755)	R(8)	26 679 867.807(608)
P(42)	25 356 086.324(643)	R(9)	26 702 043.339(622)
P(41)	25 401 032.476(555)	R(10)	26 723 428.851(700)
P(40)	25 415 759.456(459)	R(11)	26 745 533.080(728)
P(39)	25 458 998.211(395)	R(12)	26 766 354.173(792)
P(38)	25 474 789.277(315)	R(13)	26 788 448.007(850)
P(37)	25 516 394.177(271)	R(14)	26 808 642.157(885)
P(36)	25 533 177.855(206)	R(15)	26 830 786.820(992)
P(35)	25 573 221.199(177)	R(16)	26 850 291.041(979)
P(34)	25 590 927.113(127)	R(17)	26 872 548.133(1159)
P(33)	25 629 480.014(109)	R(18)	26 891 298.919(1072)
P(32)	25 648 038.830(72)	R(19)	26 913 730.480(1356)
P(31)	25 685 171.278(62)	R(20)	26 931 663.740(1167)
P(30)	25 704 514.640(39)	R(21)	26 954 332.308(1589)
P(29)	25 740 295.564(33)	R(22)	26 971 383.309(1267)
P(28)	25 760 356.034(24)	R(23)	26 994 351.983(1865)
P(27)	25 794 853.362(22)	R(24)	27 010 455.287(1377)
P(26)	25 815 564.357(21)	R(25)	27 033 787.788(2192)
P(25)	25 848 845.075(21)	R(26)	27 048 877.189(1503)
P(24)	25 870 140.808(21)	R(27)	27 072 637.922(2577)
P(23)	25 902 271.029(20)	R(28)	27 086 646.387(1656)
P(22)	25 924 086.446(20)	R(29)	27 110 900.501(3027)
P(21)	25 955 131.461(18)	R(30)	27 123 760.107(1847)
P(20)	25 977 402.180(20)	R(31)	27 148 573.557(3552)
P(19)	26 007 426.528(18)	R(32)	27 160 215.432(2089)
P(18)	26 030 088.780(21)	R(33)	27 185 655.039(4159)
P(17)	26 059 156.302(20)	R(34)	27 196 009.301(2394)
P(16)	26 082 146.866(21)	R(35)	27 222 142.814(4856)
P(15)	26 110 320.773(28)	R(36)	27 231 138.505(2775)
P(14)	26 133 576.918(20)	R(37)	27 258 034.663(5653)
P(13)	26 160 919.848(39)	R(38)	27 265 599.695(3242)
P(12)	26 184 379.270(22)	R(39)	27 293 328.288(6559)
P(11)	26 210 953.348(55)	R(40)	27 299 389.374(3806)
P(10)	26 234 554.110(33)	R(41)	27 328 021.302(7584)
P(9)	26 260 421.015(76)	R(42)	27 332 503.902(4475)
P(8)	26 284 101.484(57)	R(43)	27 362 111.241(8736)
P(7)	26 309 322.503(103)	R(44)	27 364 939.495(5260)
P(6)	26 333 021.291(91)	R(45)	27 395 595.552^(****^)
P(5)	26 357 657.387(136)	R(46)	27 396 692.223(6167)
P(4)	26 381 313.287(134)	R(47)	27 428 471.603(^****^)
P(3)	26 405 425.156(176)	R(48)	27 427 758.013(7208)
P(2)	26 428 977.084(187)	R(49)	27 460 736.676(^****^)
		R(50)	27 458 132.647(8390)

a The number in parentheses is the estimated 1 *σ* uncertainty in the last digits.

**Table 5:** Rovibrational constants (in cm^−1^) used for ^16^O^12^C^32^S

Vib. state	*v*_0_	*B*_v_	*D*_v_.× 10^11^	*H*_v_×10^14^	*L*_v_×10^17^
00^0^0	0.0	0.202 856 740 8(8)^a^	4.340 64(25)	−0.329(30)	
01^1^0	520.422 055(147)	0.203 209 834 8(21)	4.411 48(31)	−0.260(38)	
00^0^1	858.966 932(48)	0.202 251 831 6(60)	4.433 50(36)	0.045(45)	
02^2^0	1041.293 318(239)	0.203 559 482 1(89)	4.483 28(93)	−0.135(80)	
02^0^0	1047.042 048(11)	0.203 480 485(12)	4.419 64(63)	−0.712(73)	
01^1^1	1372.459 242(136)	0.202 657 042(22)	4.542 71(63)	[0.1]^b^	
03^3^0	[1562.611 159]	0.203 905 589 7(148)	4.550 22(80)	[−0.3]	
03^1^0	1573.366 413(158)	0.203 762 735 1(132)	4.453 60(113)	−0.801(206)	
00^0^2	1710.976 247(76)	0.201 635 352(32)	4.533 64(111)	0.396(102)	
02^2^1	1886.947 787(135)	0.203 048 230 7(117)	4.639 14(113)	[0.1]	
02^0^1	1892.230 557(91)	0.202 953 496 6(220)	4.554 09(123)	[−0.3]	
10^0^0	2062.200 841(121)	0.201 641 530 0(477)	4.409 80(260)	4.551(470)	−0.1155(267)
04^4^0	[2084.378 374]	0.204 248 031 9(608)	4.592 0(296)	[−0.3]	
04^2^0	2099.524 648(251)	0.204 051 914 6(232)	4.494 89(828)	[−0.6]	
04^0^0	2104.827 673(87)	0.203 968 086 4(171)	4.306 15(970)	−32.73(814)	
01^1^2	2218.028 446(175)	0.202 091 366 5(527)	4.682 71(123)	[0.6]	
03^3^1	[2402.340 630]	0.203 428 111(148)	4.734 93(580)	[0.1]	
03^1^1	2412.122 352(193)	0.203 259 077 6(203)	4399 03(43)	[−0.3]	
00^0^3	2555.991 217(126)	0.201 006 219(100)	4.647 60(514)	1.61(70)	
11^1^O	2575.307 586(181)	0.202 015 427(45)	4.473 84(210)	2.86(28)	
05^5^0	[2606.596 100]	[0.204 589 3]	[4.55]	[−0.3]	
05^3^0	2625.607 054(200)	0.204 345 249(246)	4.583 7(402)	[−0.7]	
05^1^0	2635.589 700(206)	0.204 198 969(125)	4.387 40(643)	[−0.9]	
02^2^2	[2726.564 984]	[0.202 523 2]	[4.804 7]	[0.1]	
02^0^2	2731.399 122(245)	0.202 414 701(227)	4.707(109)	[−0.3]	
10^0^1	2918.104 865(255)	0.201 102 979(109)	5.071 51(681)	54.42(147)	−1.988(95)
04^4^1	[2918.572 190]	[0.203 799 95]	[4.34]	[−0.3]	
04^2^1	2932.216 820(310)	0.203 569 074(81)	4.668 2(13)	[−0.3]	
04^0^1	2937.146 843(207)	0.203 436 472(357)	3.943 2(255)	−52.44(634)	1.71(50)
01^1^3	3057.093 032(518)	0.201 510 88(145)	4.824(81)	[2.2]	
12^2^0	[3088.908 46]	0202 382 585(146)	4.531 9(48)	[2.2]	
12^0^0	3095.554 42(9)	0.202 311 240(147)	4.500 9(56)	[2.2]	
03^3^2	[3236.415 907]	[0.202 936 096]	[4.776 6]	[−0.2416]	
03^1^2	3245.260 572(251)	0.202 744 75(42)	4.771 0(128)	[−0.2416]	
00^0^4	3393.969 128(594)	0.200 363 45(173)	4.792(96)	[2.5]	
11^1^1	3424.139 675(235)	0.201 515 951(235)	4.757 4(105)	8.78(125)	
02^2^3	[3560.106 489]	[0.201 981 318]	[4.983 28]	[2.397]	
02^0^3	3564.479 808(987)	0.201 863 71(335)	4.989(220)	[2.397]	
13^3^0	[3603.006 802]	[0.202 745 521]	[4.41]	[−1.948]	
13^1^0	3615.345 30(20)	0.202 617 950(637)	4.569(66)	6.7(189)	
04^0^2^c^	3762.825 61(83)	0.202 601 23(931)	15.06(323)	−5459.(4814)	160.(3450)
10^0^2^d^	3768.497 40(22)	0.200 840 86(229)	−14.315(842)	−3551.(1249)	9138.(864)
12^2^1	3931.301 568(234)	0.201 923 654(440)	4.782 5(153)	[6.00]	
12^0^1	3937.427 356(330)	0.201 835 302(539)	4.745 3(180)	[6.00]	
11^1^2	4266.325 10(53)	0.201 069 31(120)	6.539 9(551)	[240.0]	

a The uncertainty in the last digits (twice the estimated standard error) is given in parentheses.

b The values enclosed in square brackets were fixed during the analysis.

c Additional terms needed in the analysis were: *M* = −1.03(118) × 10^−17^ and *N* =4.26(153) × 10^−21^. See Rcf. [5.132] for discussion of analysis beyond *J* = 50.

d Additional terms needed in the analysis were: *M* = −3.21(28) × 10^−17^ and *N* =3.34(33) × 10^−21^.

**Table 6:** *l*-type resonance constants (in cm^−1^) for ^16^O^12^C^32^S

Vib. state *v*_1_ *v*_2_ *v*_3_	*q*_v_^0^×10^4^	*q*_v_*_J_×*10^10^
0 1 0	2.121 938 68(53)^a^	1.424 13(102)^b^
0 2 0	2.086 287(47)	0.659(35)
0 1 1	2.285 201(291)	3.593(114)
0 3 0	2.064 232(39)	0.252(22)
0 2 1	2.222 23(145)	1.898(64)
0 4 0	2.018 56(408)	−12.49(345)
0 1 2	2.447 445(564)	6.146(177)
0 3 1	2.183 365(144)	1.475 6(245)
1 1 0	2.155 327(274)	3.764(70)
0 5 0	2.019 577(583)	−1.318(375)
0 2 2	2.365(100)	2.76(137)
0 4 1	2.127 48(95)	−1.168(169)
0 1 3	2.596 1(162)	3.2(120)
1 2 0	2.130 1(102)	6.6(28)
0 3 2	2.302 77(231)	2.93(90)
1 1 1	2.373 76(132)	8.57(31)
0 2 3	[2.42]	[0.6587]^c^
1 3 0	2.100 35(154)	2.06(87)
1 2 1	[2.255 63]	[3.7]
1 1 2	2.709 0(144)	47.6(85)

a The uncertainty in the last digits (twice the estimated standard error) is given in parentheses.

b Also included in the fit was a higher order term *q*_v_*_JJ_* = 0.574(44) × 10^−15^.

c The values enclosed in square brackets were fixed during the analysis.

**Table 7:** Rovibrational constants (in cm^−1^) used for ^14^N^14^N^16^O

Vib. state	*v*_0_	*B*_v_	*D*_v_×10^7^	*H*_v_×10^14^	*L*_v_×10^17^
00^0^0	0.0	0.419 011 006(15)^a^	1.760 91(19)	−1.66(21)	
01^1^0	588.767 741(163)	0.419 573 590(23)	1.788 70(33)	−0.82(46)	
02^0^0	1168.132 418(198)	0.419 919 855(52)	1.871 21(85)	−8.18(368)	
02^2^0	1177.744 555(85)	0.420 124 817(41)	1.816 72(78)	5.25(504)	−2.14(69)
00^0^1	1284.903 289(124)	0.417 255 066(20)	1.725 67(24)	11.30(42)	0.444(27)
03^1^0	1749.064 972(166)	0.420 331 191(67)	1.911 42(80)	−11.89(205)	
03^3^0	1766.911 896(161)	0.420 664 523(346)	1.852 1(64)	17.4(405)	
01^1^1	1880.265 695(150)	0.417 918 446(50)	1.733 61(53)	14.15(126)	0.288(102)
10^0^0	2223.756 693(124)	0.415 559 512(18)	1.754 67(20)	−1.359(218)	
04^0^0	2322.572 934(211)	0.420 618 036(216)	1.943 02(155)	−384.4(164)	
04^2^0	2331.121 460(124)	0.420 768 166(253)	1.990 06(230)	321.2(158)	
04^4^0	[2356.251 397]^b^	[0.421 193 718]	[1.90]	[0.0]	
02^0^1	2461.996 447(242)	0.418 147 317(318)	1.892 76(175)	[0.0]	
02^2^1	2474.798 428(324)	0.418 530 238(655)	1.750 31(257)	[0.0]	
00^0^2	2563.339 334(169)	0.415 605 588(268)	1.639 20(152)	64.40(224)	
11^1^0	2798.292 466(200)	0.416 159 111(81)	1.782 02(46)	−0.47(58)	
03^1^1	3046.212 560(931)	0.418 567 39(164)	1.904 70(539)	[0.0]	
03^3^1	[3068.720 525]	[0.419 107]	[1.818]	[0.0]	
01^1^2	3165.853 959(214)	0.416 382 245(410)	1.632 92(294)	48.70(571)	
13^1^0	3931.248 302(341)	0.416 994 90(118)	1.909 53(549)	[0.0]	
13^3^0	3948.285 330(370)	0.417 327 81(195)	1.835 5(123)	[0.0]	

a The uncertainty in the last digits (twice the estimated standard error) is given in parentheses.

b The values enclosed in square brackets were fixed during the analysis.

**Table 8:** *l*-type resonance constants (in cm^−1^) for ^14^N^14^N^16^O

Vib. state *v*_1_ *v*_2_ *v*_3_	*q*_v_^0^×10^4^	*q*_v_*_J_*×10^9^
0 1 0	7.920 055 2(83)^a^	1.002 0(155)
0 2 0	7.607 30(98)	2.766(108)^b^
0 3 0	7.472 506(257)	2.889(49)^c^
0 1 1	9.083 842(493)	−2.877(32)^d^
0 4 0	7.481 95(567)	12.379(424)
0 2 1	8.206 5(118)	1.405(305)
1 1 0	7.771 651(471)	1.199 1(185)
0 3 1	8.086 4(103)	2.292(416)
0 1 2	10.722 70(197)	−10.713(76)
1 3 0	7.326 0(111)	3.062(567)

a The uncertainty in the last digits (twice the estimated standard error) is given in parentheses.

b Also included in the fit was a higher order term *q*_v_*_JJ_* = 0.123(315) × 10^−13^.

c Also included in the fit was a higher order term *q*_v_*_JI_* = −0.224(102) × 10^−13^.

d Also included in the fit was a higher order term *q*_v_*_IJ_* = 1.109(32) −10^−13^.

**Table 9:** Constants used to calculate the *v* = 1←0, *v* = 2←0, and *v* = 2←1 transition wavenumbers for ^12^C^6^O

Constant^a^	Wavenumber^b^ (cm^−1^)
Y_10_	2169.812 615(26)^c^
Y_20_	−13.287 812 0(87)
Y_30_	0.010 383 46(980)
Y_40_×10^4^	0.740 03(1300)
Y_50_×10^6^	−0.137 37(5913)
Y_01_	1.931 280 858 2(555)
Y_11_	−0.017 504 036 7(1302)
Y_21_ × 10^6^	0.486 50(4041)
Y_31_ × 10^7^	0.333 87(2754)
Y_02_×10^5^	−0.612 159 11(230)
Y_12_×10^8^	0.100 669(3765)
Y_22_×10^9^	−0.177 14(2551)
Y_03_×10^11^	0.589 265(709)
Y_13_×10^12^	−0.145 467(300)
Y_04_×10^16^	−0.360 976(20)
Y_14_×10^18^	−0.684 5(47)
Y_05_×10^22^	−0.471 36(167)

a Dunham coefficients defined by Eq. (3.2).

b To convert to frequency units multiply by 29 979.2458 MHz/cm^−1^.

c The uncertainty (twice the estimated standard error) in the last digits is given in parentheses. Excess digits are given to avoid round-off errors.

**Table 10:** Percent isotopic abundances

^12^C	98.90
^13^C	1.10
^14^N	99.634
^15^N	0.366
^16^O	99.762
^17^O	0.038
^18^O	0.200
^32^S	95.02
^33^S	0.75
^34^S	4.21
^36^S	0.02

**Table 11:** Vibrational partition function and natural abundance for the various isotopic species found in the calibration atlas

Molecule	*Q*_v_(296 K)	*N_i_*
^16^O^12^C^32^S	1.199	0.937 5
^16^O^12^C^34^S	1.203	0.041 63
^16^O^13^C^32^S	1.216	0.010 50
^16^O^12^C^33^S	1.201	0.007 40
^18^O^12^C^32^S	1.207	0.001 92
^14^N^14^N^16^O	1.1273	0.990 3
^14^N^15^N^16^O	1.1364	0.003 64
^15^N^14^N^16^O	1.1298	0.003 64
^14^N^14^N^18^O	1.1308	0.001 99
^14^N^16^O	1.000	0.994 0
^15^N^16^O	1.000	0.003 7
^14^N^18^O	1.000	0.002 0
^14^N^17^O	1.000	0.000 38
^12^C^16^O	1.000	0.986 6
^13^C^16^O	1.000	0.010 97
^12^C^18^O	1.000	0.001 98
^12^C^17^O	1.000	0.000 38
^13^C^18^O	1.000	0.000 022
^13^C^17^O	1.000	0.000 004
^12^C^32^S_2_	1.424	0.892 95
^13^C^32^S_2_	1.452	0.009 93
^12^C^34^S^32^S	1.430	0.079 13
^12^C^33^S^32^S	1.428	0.014 10

**Table 12:** Integrated intensities and transition moments for OCS

Band	Frequency interval (cm^−1^)	Measured integrated intensity (cm^−2^ atm^−1^)^e^ at 296 K	*Used in Atlas Calculation*			
			Ref.	Integrated intensity		Transition moment^a^ ${\left|R_{v^{\prime \prime }l^{\prime \prime }}^{v^{\prime }l^{\prime }}\right|}^{b}$ (D)^d^
				(cm^−2^ atm^1^)	(cm/molecule) × 10^19^	
				at 296 K		
*v*_2_	494–567	11.9(8)^c^	[5.28]	11.85	4.78	0.047
		12.0(8)	[5.89]			
*v*_3_	812–890	41.(4)	[5.49]	36.3	14.64	0.064
		35.4(16)	[5.85]			
		35.5(23)	[5.89]			
		36.3(10)	[5.103]			
		36.3(1)	[5.127]			
2*v*_2_	1000–1095	14.9(15)	[5.49]	13.8^f^	5.57	0.0333
		13.3(9)	[5.89]			
		12.9^f^	[5.118]			
		12.7^f^	[5.134]			
2*v*_3_	1650–1739	7.6(16)	[5.49]	6.9	2.78	0.0195
		6.71(47)	[5.89]			
2*v*_2_ *+ v*_3_	1832–1934	12.4(25)	[5.49]	11.2	4.52	0.022
		10.9(8)	[5.89]			
*v*_1_	1970–2091	2520.(250)	[5.49]	2533.	1021.7	0.345
		2980.(220)	[5.89]			
4*v*_2_	2080–2141	19.5(49)	[5.49]	10.4^f^	4.19	0.024
		9.6^f^	[5.101]			
3*v*_3_	2510–2574		[5.132]	0.35	0.14	0.00367
*v*_1_ + *v*_2_	2550–2600	0.147(6)^f^	[5.104]	0.149^f^	0.060	0.00257
2*v*_2_ + 2*v*_3_	2693–2763	0.52(8)	[5.49]	0.52	0.21	0.004
*v*_1_ + *v*_3_	2862–2941	37.6(40)	[5.49]	35.5	14.3	0.034
		33.6(22)	[5.89]			
4*v*_2_ + *v*_3_	2910–2970	0.32^f^	[5.132]	0.32^f^	0.13	0.0036
*v*_1_ + 2*v*_2_	3065–3120	3.01(30)	[5.49]	2.43	0.98	0.008
		2.25(16)	[5.89]			

a See the key page for each band region for transition moments of hot bands that are not the same as for the ground state transitions.

b See Eq. (3.24) for definition of $\left|R_{v^{\prime \prime }l^{\prime \prime }}^{v^{\prime }l^{\prime }}\right|.$.

c 1 cm^−2^ atm^−1^ at 296 K = 4.0335 × 10^−20^ cm/molecule.

d 1D = 3.336 × 10^−30^ C m.

e The uncertainties in the last digits are given in parentheses.

f Hot band intensities were not included.

**Table 13:** Integrated intensities and transition moments for N_2_O

				*Used in Atlas Calculation*		
Band	Frequency interval	Measured integrated intensity	Ref.	Integrated intensity		Transition moment^a^
	(cm^−1^)	(cm^−2^ atm^−1^)^c^		(cm^−2^ atm^1^)	(cm/molecule) ×10^19^	${\left|R_{v^{\prime \prime }l^{\prime \prime }}^{v^{\prime }l^{\prime }}\right|}^{b}$ (D)^d^
		at 296 K		at 296 K		
*v*_2_	520–660	28.9(15)^e^,^f^	[5.214]	24.5	9.88	0.0692
		30.4(40)^f^	[5.151]	29.2^f^		
		31.4(40)^f^	[5.157]			
*v*_1_ – *v*_3_	880–990	0.0524	[5.209]	0.052	0.021	0.056
		0.055	[5.172]			
*v*_1_ – 2*v*_2_	990–1090	**–**	**–**	0.01	0.004	0.018
2*v*_2_	1110–1225	7.00(3)	[5.218]	6.66	2.69	0.0247
		7.36(7)	[5.216]	8.30^f^		
		6.98	[5.237]			
		8.27(43)^f^	[5.214]			
		8.49(9)^f^	[5.234]			
*v*_3_	1200–1340	207.(1)	[5.218]	206.	83.1	0.1326
		225.(12)^f^	[5.214]	232.5^f^		
		226.(2)^f^	[5.234]			
		229.8(45)	[5.216]			
*v*_2_ + *v*_3_	1835–1925	0.52(3)	[5.212]	0.52	0.21	0.00548
		0.58(10)^f^	[5.214]	0.60^f^		
		0.437(20)	[5.185]			
*v*_1_	2140–2269	1207.(22)	[5.226]	1206.	486.4	0.244
		1421.(76)^f^	[5.214]	1358.^f^		
		1189.(30)	[5.187]			
		1302.(50)	[5.171]			
		1301.(54)^f^	[5.198]			
2*v*_2_ + *v*_3_	2410–2510	6.79(4)	[5.216]	6.7	27	0.017
		7.35(38)^f^	[5.214]	8.2^f^		
2*v*_3_	2490–2605	30.86(14)	[5.216]	32.1	12.9	0.037
		32.1(17)^f^	[5.214]	36.3^f^		
*v*_1_ + *v*_2_	2725–2840	2.24(13)^f^	[5.214]	1.895	0.764	0.0086
		2.11	[5.235]	2.26^f^		

a Unless indicated otherwise (see footnote f), the integrated intensities are given only for the vibrational transition from the ground state.

b See Eq. (3.24) for definition of $\left|R_{v^{\prime \prime }l^{\prime \prime }}^{v^{\prime }l^{\prime }}\right|$.

c 1 cm^−2^ atm^−1^ at 296 K = 4.0335 × 10^−20^ cm/molecule.

d 1D = 3.336 × 10^−30^ C m.

e The uncertainties in the last digits are given in parentheses.

f All hot bands and isotopes are included.

**Figure:**
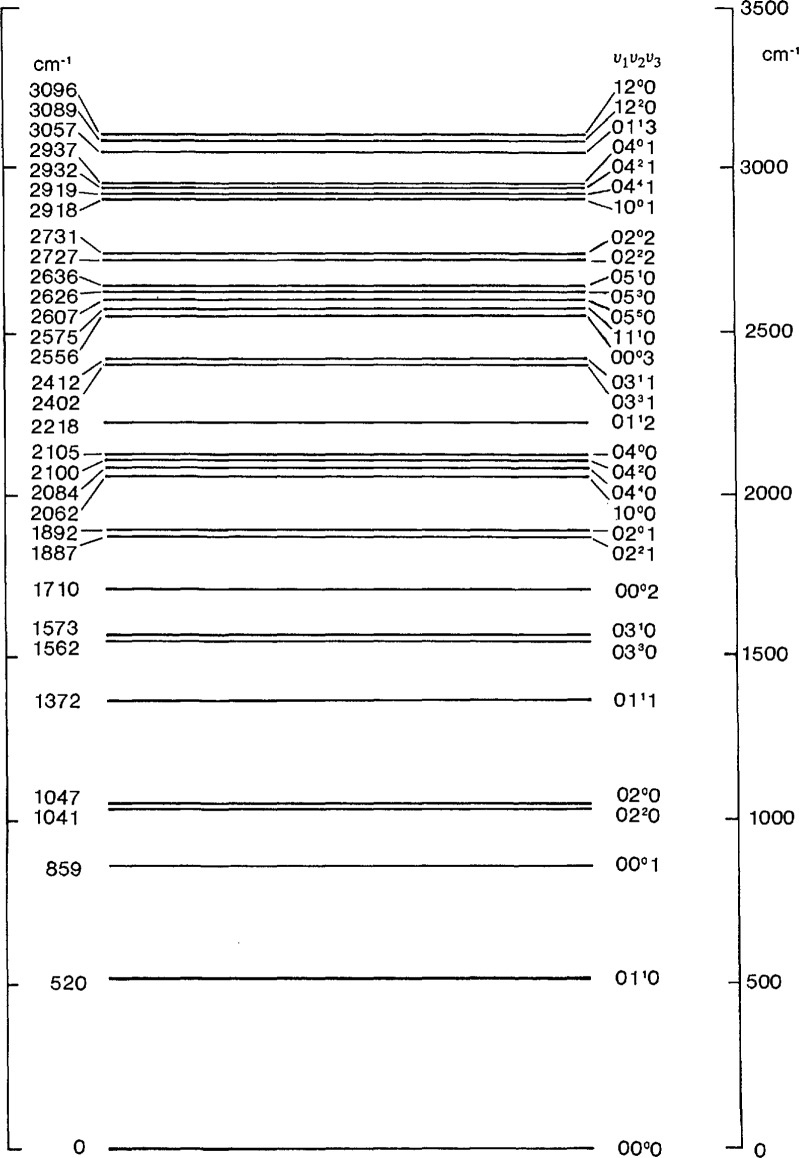
Energy levels for OCS

**Figure:**
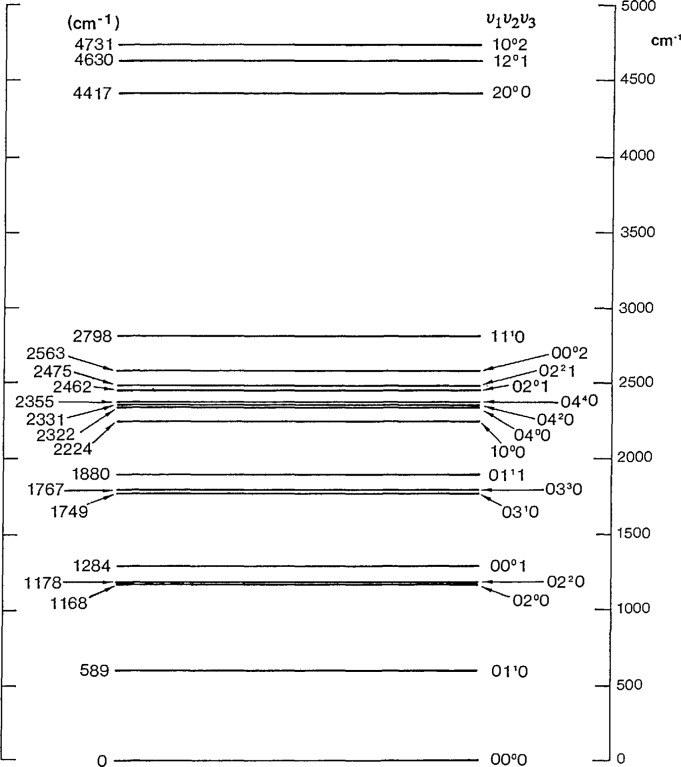
Energy levels for N_2_O

## Footnotes

3 The references in Sec. 1 are listed at the end of the section as [1.1], [1.2], etc.

4 Rather than repeat references given in the bibliography (Sec. 5), they are referenced as [5.XX].

## 7. Appendix A: Useful Equations and Formulas

Conversion factors

dipole moment or transition moment:
  $$\;1D=3.335\;64\times 10^{-30}\;\mathrm{Cm}$$
wavenumber:
  $$\;1\;{\mathrm{cm}}^{-1}=29\;979.2458\;\mathrm{MHz}$$
pressure:
  $$\begin{array}{l} 1\;\mathrm{atm}=101\;325.\;\mathrm{Pa}\left(\text{pascal}\right)\\ 1\;\mathrm{Torr}=133.322\;\mathrm{Pa} \end{array}$$
absorption intensity:
  $$1\;{\mathrm{cm}}^{-2}\;{\mathrm{atm}}^{-1}\;\mathrm{at}\;296\;K=4.033\times 10^{-20}\mathrm{cm}/\text{molecule}$$

Useful equations

Boltzmann factor:
  $$\;N_{i}=N_{j}\exp\left(-1.439E_{ij}/T\right),$$
  where *N_i_* is the population of the *i* th level and *N_j_* is the population of the *j* th level with energy difference in cm^−1^ of *E_ij_* at a temperature of *T* kelvins.
Doppler half-width at half intensity, *γ*_D_:
  $$\;\gamma_{D}=3.581\times 10^{-7}v{\left(T/M\right)}^{1/2},$$
  where *v* is the frequency in the same units as *γ*_D_, *T* is the temperature in kelvins, and *M* is the mass of the molecule in atomic mass units.
Peak intensity of a Doppler shaped line:
  $$\%\;\text{transmission}=100\exp\left(-1.1494\times 10^{14}\;Slp/\gamma_{D}\right),$$
  where *S* is the intensity given in the tables, *l* is the pathlength in cm, and *p* is the pressure in Pa.
Peak intensity of a Lorentzian line:
  $$\;\%\;\text{transmission}=100\exp\left(-0.7789\times 10^{14}\;Slp/\gamma_{L}\right),$$
  where *S* is the intensity given in the tables, *γ*_L_ is the Lorentzian half-width at half height, *l* is the path-length in cm, and *p* is the pressure in Pa.

## 8. Appendix B. Energy Level Diagrams for OCS and N_2_O

### Information on NIST SP821 and Standard Reference Database 39

NIST Special Publication 821 Wavenumber Calibration Tables from Heterodyne Frequency Measurements, by Arthur G. Maki and Joseph S. Wells, contains not only the spectral maps with facing tables, but also includes the text duplicated in this journal. A limited number of hard bound copies are still available from the authors, or NIST SP 821 may be ordered from:

Superintendent of Documents,
U.S. Government Printing Office,
Washington, DC 20402

Some users are not interested in spectral maps and prefer to have only the tabular information, (for example to load files into a computer). For these users we offer an alternative of having only diskettes of the tables. We have included energy level diagrams of both OCS and N_2_O in Appendix B for the convenience of these users.

In preparing the tables for this atlas, far more transitions were calculated than could possibly be published. A complete list of transitions calculated for this work is available as NIST Standard Reference Database 39, Wavelength Calibration Tables, in the form of four diskettes for a personal computer. These diskettes, written in ASCII, contain the following information: wavenumber (cm^−1^), uncertainty, lower state energy (cm^−1^), intensity (cm/molecule), vibrational assignment, rotational assignment, date of data entry, isotopic species, and band designation according to the keys given in this book. The authors intend to update the database periodically. Persons interested in obtaining the most up-to-date calibration data should contact:

Standard Reference Data,
National Institute of Standards and Technology
Bldg. 221/Room A320,
Gaithersburg, Maryland 20899
(301) 975-2208
Fax (301) 926-0416

